# Antipsychotic dose and efficacy for acute schizophrenia spectrum disorders: an updated systematic review and dose-response meta-analysis

**DOI:** 10.1016/j.eclinm.2026.103847

**Published:** 2026-03-26

**Authors:** Yuki Furukawa, Xiao Lin, Alessandro Rodolico, Jing Tian, Hui Wu, Johannes Schneider-Thoma, Josef Priller, John M. Davis, Stefan Leucht, Spyridon Siafis

**Affiliations:** a Technical University of Munich, TUM School of Medicine and Health, Department of Psychiatry and Psychotherapy, Munich, Germany; b German Center for Mental Health (DZPG), partner site Munich/Augsburg, Munich, Germany; c Department of Neuropsychiatry, University of Tokyo, Tokyo, Japan; d Neuropsychiatry and Laboratory of Molecular Psychiatry, Charité - Universitätsmedizin Berlin and Deutsches Zentrum für Neurodegenerative Störungen, Berlin, Germany; e University of Edinburgh and UK DRI, Edinburgh, UK; f Psychiatric Institute, University of Illinois Chicago, Chicago, USA; g Department of Psychiatry, Johns Hopkins University, Baltimore, USA

**Keywords:** Schizophrenia, Antipsychotics, Meta-analysis, Dose-response meta-analysis

## Abstract

**Background:**

The optimal antipsychotic doses for achieving efficacy in acute schizophrenia spectrum disorders have been investigated but uncertainties remained. We conducted one-stage dose-response meta-analyses to elucidate the dose-response relationships of antipsychotics in acute schizophrenia by utilizing a larger amount of information than two-stage approach.

**Methods:**

We searched Cochrane Schizophrenia Group's register until 13.01.2025 and PubMed until 19.01.2026 for fixed-dose studies investigating 20 antipsychotics regardless of their formulations in adults and in children/adolescents with acute schizophrenia. The primary outcome was overall schizophrenia symptoms, summarized using standardized mean difference (SMD). We conducted one-stage random-effects dose-response meta-analyses with restricted cubic splines, analyzing each drug separately and all drugs combined. We evaluated confidence in the evidence using GRADE. People with lived experience were not involved. The protocol was registered with PROSPERO (CRD42020181467).

**Findings:**

We included 131 studies with 40,715 participants analyzed (for adults, mean age 39.24 years, 31.8% females; for children/adolescents, 15.68 years, 45.1% females; ethnicity data not available). Dose-response curves of most antipsychotics on overall symptoms in people with acute exacerbations of schizophrenia were hyperbolic, reaching plateau within the lower-to-medium approved dose ranges, around 3–5 mg risperidone dose equivalents. There was low to very low certainty of evidence regarding the dose-response relationships of amisulpride, blonanserin, cariprazine, clozapine, haloperidol, lumateperone and ziprasidone. We did not find enough studies to conduct dose-response meta-analysis for olanzapine/samidorphan, xanomeline/trospium, and zotepine. Dose-response curves did not clearly differ in children and adolescents, but data were sparse and with low or very low certainty.

**Interpretation:**

Antipsychotics typically exert the majority of their therapeutic effects at doses within the lower to middle range of their recommended doses. While this finding provides a general reference for clinical use, individual variability is expected in practice. Further investigation is required to elucidate the influence of potential effect modifiers on the dose-response relationships.

**Funding:**

This meta-analysis was conducted within the framework of a larger project funded by the German Research Foundation (Deutsche Forschungsgemeinschaft, DFG; grant #468853597) and within activities of the German Center for Mental Health (Deutsches Zentrum für Psychische Gesundheit, DZPG), Munich-Augsburg partner site, funded by the Federal Ministry of Research, Technology and Space (BMFTR, grant #01EE2303B). This study was also partly funded by the SENSHIN Medical Research Foundation grant given to YF.

**Research in context**

**Evidence before this study**

Antipsychotics are the mainstay pharmacological treatment for schizophrenia spectrum disorders. Determining the optimal dosing for achieving efficacy is important, yet evidence-based information beyond the approved and expert consensus-recommended dose ranges remains sparse. We searched PubMed for meta-analyses on dose-response relationships for antipsychotic efficacy using the search terms “schizophrenia”, “antipsychotic”, “dose-response” and restricting to meta-analysis article type, up to 20th August 2025. From 88 references, we identified 14 meta-analyses: eight were on a single antipsychotic, three focused on adverse events, and three dose-response meta-analyses analyzed multiple antipsychotics. Previous dose-response meta-analyses on adults with schizophrenia conducted by our group found that both acute and long-term efficacy tended to plateau at risperidone-equivalent doses of 3–5 mg/day. However, the number of studies included for several widely used antipsychotics was small, e.g., one study for haloperidol, two for olanzapine, and three for risperidone in analyses of acute efficacy, both in our work and in another meta-analysis focusing on positive and negative symptoms. This limited evidence leaves uncertainty about the precise dose at which efficacy plateaus, or whether efficacy might continue to increase at higher doses for certain antipsychotics, such as olanzapine.

**Added value of this study**

We expanded on previous meta-analyses by applying a one-stage dose-response meta-analysis, which allows the inclusion of studies comparing a single dose with placebo unlike the previously used two-stage method, and analyzed the most comprehensive, up-to-date dataset of 131 blinded and open-label randomised controlled trials involving 40,715 participants with acute schizophrenia treated with fixed doses of antipsychotics or placebo. This approach increased the number of studies available for analysis of individual antipsychotics (e.g., 22 for haloperidol, 22 for olanzapine, and 28 for risperidone), allowing for more precise and robust estimates of dose-response relationships. All individual antipsychotics demonstrated, on average, a plateau in efficacy beyond a certain dose, which generally corresponded to the defined daily doses and the lower-to-middle range of expert consensus-recommended dosage ranges. When combining all antipsychotics, we replicated a plateau in efficacy at approximately 3–5 mg/day of risperidone equivalents, and this pattern was broadly similar between children/adolescent and adult populations.

**Implications of all the available evidence**

On average, antipsychotics achieve most of their efficacy within the lower-to-middle range of their recommended dosages, with little to no additional benefit at higher doses. These findings can inform treatment decisions by considering potential dose-related side effects, and individual differences. Further investigation of the effects of participant characteristics, such as age and sex, using individual-participant-data is warranted.

## Introduction

Choosing the appropriate antipsychotic dose in the treatment of schizophrenia is crucial to ensure efficacy and tolerability. Clinical practice guidelines recommend starting with a low dose and gradually titrating upwards, considering both the efficacy and side effects. They present initial dose, empirically typical dose range, and approved maximum dose, but not the optimal target dose range.^1^^,^^2^ Although consensus guidelines on antipsychotic dosing have been published,^3^ robust, evidence-based guidance remains lacking. This gap is critical because adverse events increase with higher doses.^4^, ^5^, ^6^, ^7^ Therefore, it is of paramount importance that clinicians know the dose-response relationships when they prescribe antipsychotics.

Attempts have been made to clarify the dose-response relationship of antipsychotics by synthesizing data from randomized trials. Davis and Chen manually plotted dose-response curves of antipsychotic efficacy in 2004,^8^ and more recently, dose-response meta-analyses have used statistical modelling to estimate such curves.^9^^,^^10^ These earlier analyses applied a two-stage dose-response meta-analysis, in which dose-response relationships are first estimated within each study and then synthesized together in a second stage. However, this approach typically excludes studies that examined fewer than three dose levels of the same antipsychotic or placebo. As a result, dose-response curves for some widely used drugs, including haloperidol, olanzapine, and risperidone, were based on a small number of studies, making their findings inconclusive.

A more advanced method, called one-stage dose-response meta-analysis, synthesizes all available data in a single stage to estimate the dose-response relationship, and allows also the inclusion of two-arm studies (e.g., comparing a single dose versus placebo),^11^ thereby expanding the pool of eligible studies and enabling more robust estimations. Our group has recently applied this method to investigate dose-response relationships in relapse prevention,^12^ tolerability,^13^ and side effects.^4^, ^5^, ^6^, ^7^

We aimed to update our previous systematic review^9^ and apply a one-stage dose-response meta-analysis^11^ to provide more robust estimates of the dose-response relationship for each antipsychotic on the overall efficacy in adults and children/adolescents with acute schizophrenia.

## Methods

We followed the PRISMA statement (eAppendix-1).^14^ The protocol was registered in PROSPERO (CRD42020181467) (details and amendments in eAppendix-2).

### Eligibility criteria and search strategies

#### Study design

We included open and blinded randomized controlled trials (RCTs) comparing fixed doses of antipsychotics or placebo in people with acute exacerbation of schizophrenia with a treatment duration of at least 3 weeks. We excluded trials that compared different antipsychotics head-to-head only, or trials on relapse prevention.

#### Participants

We included people with acute exacerbations of schizophrenia spectrum disorders, i.e., schizophrenia, schizoaffective and schizophreniform disorder, as defined by any criteria. We planned to analyze separately studies on children/adolescents (<18 years old) and on individuals of advanced age (>65 years old), because they may require different doses.^15^ We excluded studies focusing on people with predominant negative symptoms, whose dose-response curve clearly differed.^9^ We plan to report the update for those with predominant negative symptoms in another study examining positive and negative symptoms separately.

#### Interventions

We included trials that compared either at least two different doses of the same antipsychotic (any second-generation antipsychotic or haloperidol) with each other and/or placebo, or a single antipsychotic dose with placebo. We included doses within and outside the licensed dose. We included fixed-dose regimens, as well as flexible-dose regimens with narrow dose ranges. We lumped different formulations using daily oral equivalents (eAppendix-3) and examined them separately in sensitivity analysis.

#### Outcome

The outcome was overall efficacy measured with the Positive and Negative Syndrome Scale (PANSS) total score^16^ or the Brief Psychiatric Rating Scale,^17^ or any other validated rating scale. We prioritized change over endpoint scores, and results accounting for missing outcome data over completer or per-protocol. We used the standardized mean difference (SMD) as the summary measure.

#### Search strategies

We searched the Cochrane Schizophrenia Group Register interventions up to 13.01.2025^18^ and PubMed from 01.01.2025 to 19.01.2026 (details in eAppendix-2).

### Data extraction and risk of bias assessment

Two reviewers (YF, XL, JT, HW, JST, SL, SS) independently selected trials, extracted data, and evaluated the risk of bias of individual studies using Cochrane Risk of Bias tool 1,^19^ resolving disagreements through discussion.

### Data analysis

We estimated dose-response curves for each antipsychotic using one-stage random-effects dose-response meta-analyses in a frequentist framework, applying restricted cubic splines with three knots.^11^ Knot locations were set at the 25th, 50th and 75th percentiles of the available doses,^11^ and alternative locations were examined in *post-hoc* sensitivity analyses (eAppendix-11).

We examined the shape of the dose-response relationships visually, supplemented by Wald statistical tests for the model coefficients, and by estimating the doses achieving 50% and 95% of maximal efficacy (ED50, ED95).

Between-study heterogeneity was investigated with the variance partition coefficient (VPC).^11^ Heterogeneity was further explored by examining the distribution of potential effect modifiers (age, sex, baseline severity, treatment duration, risk of bias) across the examined doses for each drug. We examined small-study effects with funnel plots and a dose-response meta-regression with the squared root of the sample size.

We analyzed patient subgroups based on age separately (see “Participants”) and evaluated the robustness of the results in multiple predefined and *post hoc* sensitivity analyses (eAppendix-2/11).

As most antipsychotics share a common mechanism of action as dopamine D2 receptor antagonists or partial agonists, and show no clearly distinct differences in efficacy,^20^ we also conducted pooled analyses after converting individual doses to risperidone equivalents, based on the estimated ED95 values, with alternative methods examined in sensitivity analyses.

We evaluated the confidence in the evidence by adapting the Grading of Recommendations Assessment, Development and Evaluation (GRADE) framework to dose-response meta-analysis^6^^,^^21^^,^^22^ (see detailed descriptions in eAppendix-12).

Data analyses were performed with R v4.5.0,^23^ using meta package v8.2.0^24^ and dosresmeta package v2.2.0.^25^

### Role of funding source

The funders of the study had no role in study design, data collection, data analysis, data interpretation, or writing of the report.

## Results

**Fig. 1:**
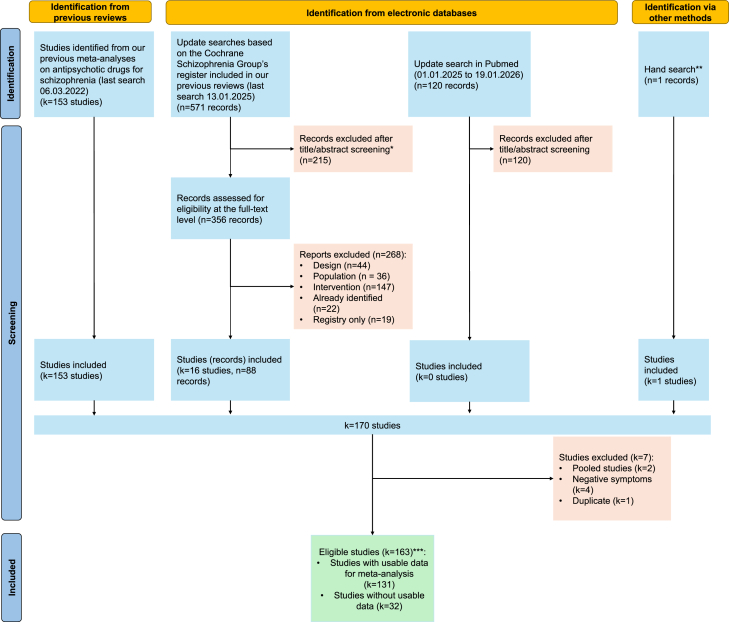
**Screening flow diagram**. Please see Appendix-4 for a more detailed description of the study selection. ∗Broad selection criteria of our group's database: We excluded studies that are not RCTs (randomized controlled trial) studies with a duration less than 3 weeks, population less than 80% schizophrenia, studies conducted and published in China only, no useable comparison (studies investigating combinations of antipsychotics, studies which compared an included antipsychotic drug to another intervention which is not among the list of included antipsychotics or placebo, studies which compare antipsychotics grouped to drug classes such as “any FGA” (first generation antipsychotics)). ∗∗McEvoy 1991 was added. ∗∗∗12 studies found in the update search were already included in the previous dose-response meta-analyses of our group. In the update searches additional references to these studies were found.

**Fig. 2:**
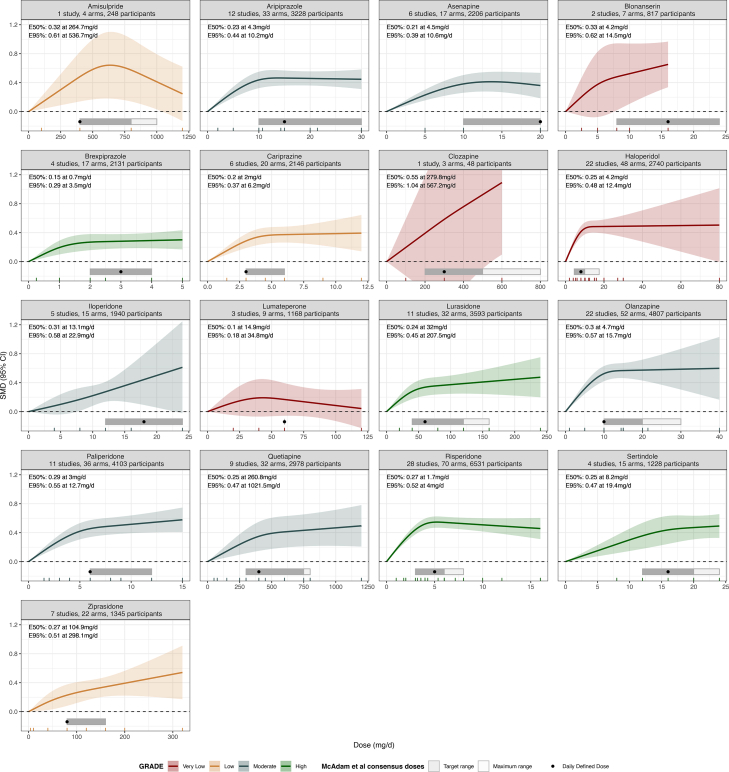
**Dose-response curves for each antipsychotic in adults with acute schizophrenia**. The figure shows the dose-response relationship between antipsychotic doses and efficacy compared to placebo (0 mg/day) for overall symptoms in adults with acute schizophrenia. The solid line represents the mean dose-response relationship, and the shaded area shows the 95% confidence intervals. The colour key indicates confidence in the evidence (red for very low, yellow for low, blue for moderate, and green for high confidence in the evidence). The rug plot displays the dose levels available in the dose-response meta-analysis. The ribbons above the x-axis represent the target (grey) and maximum (white) dose ranges recommended by the consensus of MacAdam et al., 2023,^3^^,^^26^ while the black dots indicate the Defined Daily Dose (DDD). For blonanserin no consensus-based target dose range or DDD was available, so we used the licensed dose range as the target dose range, and the mean dose delivered (∼16 mg/day) in flexible-dose arms of clinical trials for presentation. E50% and E95% represent the dose at which an antipsychotic achieves 50% and 95% of the maximum effect size (vs placebo), as observed in the dose-response meta-analysis. mg/day = milligrams per day (for long-acting injectables and transdermal formulations, equivalent oral dose was calculated first).

**Fig. 3:**
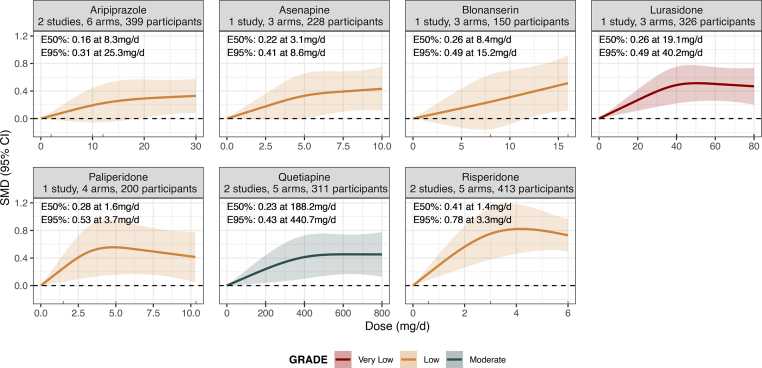
**Dose-response curves for each antipsychotic in children/adolescents with acute schizophrenia**. The figure shows the dose-response relationship between antipsychotic doses and efficacy compared to placebo (0 mg/day) for overall symptoms in children/adolescents with acute schizophrenia. The solid line represents the mean dose-response relationship, and the shaded area shows the 95% confidence intervals. The colour key indicates confidence in the evidence (red for very low, yellow for low, blue for moderate, and green for high confidence in the evidence). The rug plot displays the dose levels available in the dose-response meta-analysis. E50% and E95% represent the dose at which an antipsychotic achieves 50% and 95% of the maximum effect size (vs placebo), as observed in the dose-response meta-analysis. mg/day = milligrams per day (for long-acting injectables and transdermal formulations, equivalent oral dose was calculated first).

We identified 131 randomized trials with 437 arms with 40,715 participants analyzed. Fig. 1 shows the screening process and eAppendices-5 and-6 show the characteristics of studies without useable data and studies included the analyses. Of the 20 antipsychotics, dose-response meta-analysis was possible for 17 antipsychotics in adult populations (Fig. 2) and 7 in children/adolescents (Fig. 3). There were no eligible fixed-dosing studies for xanomeline/trospium,^27^, ^28^, ^29^ and no useable data for older populations.

The mean age in adult studies was 39.24 years, and one-third of the participants were females (31.8% [12,561/39,549 reported sex data]). In children/adolescent studies, the mean age was 15.68 years and about half of the participants were female (45.1% [980/2172]). The mean baseline PANSS total score was approximately 95 in both age groups. Most studies were conducted in inpatient settings (n = 93), with 22 in mixed inpatient/outpatient settings, 3 in outpatient-only settings, and 13 not reporting clearly the setting. The median study duration was 6 weeks (range, 3–26 weeks). The overall risk of bias of included studies was low for 77 studies, moderate for 47, and high for 7 (eAppendix-7). We did not observe clear imbalances in these characteristics across doses (eAppendix-13).

### Dose-response meta-analyses

#### Amisulpride

A bell-shaped dose-response curve was observed, with maximum efficacy achieved between 500 and 600 mg/day and low confidence in the evidence (ED50 = 264.7 mg/d, SMD = 0.32 compared to placebo; ED95 = 536.7 mg/d, SMD = 0.61; number of studies n = 1; number of arms k = 4; number of participants N = 248; VPC not estimable; Wald-test for the overall dose-response relationship p = 0.02). The single study included was a dose-finding study with 100 mg, 400 mg, 800 mg and 1200 mg arms.

#### Aripiprazole

A hyperbolic relationship was observed reaching plateau between 8 and 12 mg/day and moderate confidence (ED50 = 4.3 mg/d, SMD = 0.23; ED95 = 10.2 mg/d, SMD = 0.44; n = 12, k = 33, N = 3228; median VPC = 23.3%; Wald-test p < 0.001). The findings were similar for oral and long-acting injection formulations (eAppendix-11). Alternative knot locations did not change the overall shape of the curve, but the plateau dose ranged from 10 to 20 mg/d (eAppendix-11).

In children/adolescents, the dose-response curve showed wide confidence intervals across the examined dose range, with low confidence in the evidence (ED50 = 8.3 mg/d, SMD = 0.16; ED95 = 25.3 mg/d, SMD = 0.31; n = 2; k = 6; N = 399; median VPC = 0%; Wald-test p-value = 0.03).

#### Asenapine

A hyperbolic relationship was observed reaching plateau between 8 and 12 mg/d with moderate confidence (ED50 = 4.5 mg/d, SMD = 0.21; ED95 = 10.6 mg/d, SMD = 0.39; n = 6; k = 17; N = 2206; median VPC = 36.4%; Wald-test p-value <0.001). The findings were similar for oral and transdermal formulation (eAppendix-11).

In children/adolescents, the dose-response curve was hyperbolic reaching plateau between 5 and 7 mg/d with low confidence in the evidence (ED50 = 3.1 mg/d, SMD = 0.22; ED95 = 8.6 mg/d, SMD = 0.41; n = 1; k = 3; N = 228; VPC not estimable; Wald test p = 0.02).

#### Blonanserin

There was a monotonic increase of the efficacy with higher doses, yet with very low confidence in the evidence (examined dose range, 0–16 mg oral equivalent; ED50 = 4.2 mg/d, SMD = 0.33; ED95 = 14.5 mg/d, SMD = 0.62; n = 2; k = 7; N = 817; median VPC = 71.3%; Wald test p < 0.001). Both the oral formulation and transdermal formulation had a monotonically increasing trend among the dose range examined (oral, 0 mg–10 mg; transdermal, 0 mg–80 mg), and the results of sensitivity analyses using different knot positions varied (eAppendix-11).

In children/adolescents, there was a monotonic increase of the efficacy with higher doses with low confidence in the evidence, yet with wide confidence interval (dose range examined, 0 mg–16 mg; ED50 = 8.4 mg/d, SMD = 0.26; ED95 = 15.2 mg/d, SMD = 0.49; n = 1; k = 3; N = 150; VPC not applicable; Wald test p = 0.04).

#### Brexpiprazole

The dose-response curve was hyperbolic reaching plateau between 1 and 2 mg/d with high confidence in the evidence (ED50 = 0.7 mg/d, SMD = 0.15; ED95 = 3.5 mg/d, SMD = 0.29; n = 4; k = 17; N = 2131; median VPC = 0%; Wald test p < 0.001).

#### Cariprazine

The dose-response curve was hyperbolic reaching plateau between 3 and 5 mg/d with low confidence in the evidence (ED50 = 2.0 mg/d, SMD = 0.20; ED95 = 6.2 mg/d, SMD = 0.37; n = 6; k = 20; N = 2146; median VPC = 3.6%; Wald test p < 0.001).

#### Clozapine

There was a monotonic increase of the efficacy with higher doses, yet with very wide confidence interval and very low confidence in the evidence (examined dose range, 100–600 mg; ED50 = 279.8 mg/d, SMD = 0.55; ED95 = 567.2 mg/d, SMD = 1.04; n = 1; k = 3; N = 48; median VPC not applicable; Wald test p = 0.05). The single study included was a dose-finding study with 100 mg, 300 mg and 600 mg arms.

#### Haloperidol

The dose-response curve was hyperbolic reaching plateau between 8 and 12 mg/d with very low confidence in the evidence (ED50 = 4.2 mg/d, SMD = 0.25; ED95 = 12.4 mg/d, SMD = 0.48; n = 22; k = 48 N = 2740; median VPC = 0%; Wald test p < 0.001). All the included studies used the oral formulation. We downgraded for the indirectness domain because less than 25% of the participants were allocated to the arms with doses lower than 10 mg/d (i.e., the upper limit of the recommended target dose range; eAppendix-12). It is unlikely that haloperidol higher than 10 mg would provide additional benefit, but the dose-response relationship below 10 mg/d remain therefore uncertain.

#### Iloperidone

There was a monotonic increase of the efficacy with higher doses with moderate confidence in the evidence, yet with wide confidence interval (ED50 = 13.1 mg/d, SMD = 0.31; ED95 = 22.9 mg/d, SMD = 0.58; n = 5; k = 15; N = 1940; median VPC = 32.6%; Wald test p < 0.001). The shape of the curve depended on the locations of knots, and sensitivity analyses with different knot locations showed hyperbolic shapes, reaching plateau between 15 and 20 mg/d (eAppendix-11).

#### Lumateperone

The dose-response curve was hyperbolic reaching plateau between 30 and 40 mg/d with very low confidence in the evidence (ED50 = 14.9, SMD = 0.10; ED95 = 34.8 mg/d, SMD = 0.18; n = 3; k = 9; N = 1168; median VPC = 59.7%; Wald test p = 0.34).

#### Lurasidone

The dose-response curve bended at doses around 50–70 mg/d, after which further increase in efficacy became smaller, gradually approaching a plateau, with high confidence in the evidence (ED50 = 32 mg/d, SMD = 0.24; ED95 = 207.5 mg/d, SMD = 0.45; n = 11; k = 32; N = 3593; median VPC = 43.3%; Wald test p < 0.001).

In children/adolescents, the dose-response curve was hyperbolic reaching plateau between 40 and 50 mg/d with very low confidence in the evidence (ED50 = 19.1 mg/d, SMD = 0.26; ED95 = 40.2 mg/d, SMD = 0.49; n = 1; k = 3; N = 326; VPC not applicable; Wald test p < 0.001).

#### Olanzapine

The dose-response curve was hyperbolic reaching plateau between 8 and 12 mg/d with moderate confidence in the evidence (ED50 = 4.7 mg/d, SMD = 0.30; ED95 = 15.7 mg/d, SMD = 0.57; n = 22; k = 52; N = 4807; median VPC = 51.1%; Wald test p < 0.001). Most of the studies used the oral formulations (n = 21 out of 22; k = 48 out of 52; N = 4405 out of 4807). The long-acting injection formulation showed a monotonical increase among the dose range examined (range, 0 mg–300 mg/2 weeks), but it was in line with the overall finding, because 300 mg/2 weeks is around 20 mg/d oral equivalent (eAppendix-11). Sensitivity analyses using different knot locations did not change the overall shape of the curve, but the plateau dose ranged from 10 to 15 mg/d (eAppendix-11).

#### Olanzapine/samidorphan

We only found one two-arm study of a placebo-controlled trial, with which we could not conduct dose-response meta-analysis. The examined dose was olanzapine 20 mg/d and samidorphan 10 mg/d with SMD 0.31 (95% CI, 0.06–0.57; n = 1; k = 2; N = 236).

#### Paliperidone

The dose-response curve bended at doses around 5–8 mg/d, after which further increase in efficacy became smaller, gradually approaching a plateau, with moderate confidence in the evidence (ED50 = 3.0 mg/d, SMD = 0.29; ED95 = 12.7 mg/d, SMD = 0.55; n = 11; k = 36; N = 4103; median VPC = 55.4%; Wald test p < 0.001). Both the oral and long-acting injection formulation were in line with the main finding (eAppendix-11). Sensitivity analyses using different knot positions did not change the overall shape of the curve, but the plateau dose ranged from 5 to 10 mg/d (eAppendix-11).

In children/adolescents, the dose-response curve was hyperbolic reaching plateau between 3 and 5 mg/d with low confidence in the evidence (ED50 = 1.6 mg/d, SMD = 0.28; ED95 = 3.7 mg/d, SMD = 0.53; n = 1; k = 4; N = 200; VPC not applicable; Wald test p = 0.016).

#### Quetiapine

The dose-response curve bended at doses around 400–600 mg/d, after which further increase in efficacy became smaller, gradually approaching a plateau, with moderate confidence in the evidence (ED50 = 260.8 mg/d, SMD = 0.25; ED95 = 1021.5 mg/d, SMD = 0.47; n = 9; k = 32; N = 2978; median VPC = 70.4%; Wald test p < 0.001). The immediate-release formulation was bell-shaped peaking around 300–400 mg/d, and the extended-release formulation showed a monotonic increase in the dose range examined (range, 0–800 mg/d) (eAppendix-11).

In children/adolescents, the dose-response curve was hyperbolic reaching plateau between 400 and 500 mg/d with moderate confidence in the evidence (ED50 = 188.2 mg/d, SMD = 0.23; ED95 = 440.7 mg/d, SMD = 0.43; n = 2; k = 5; N = 311; median VPC = 0.2%; Wald test p = 0.008).

#### Risperidone

The dose-response curve was hyperbolic reaching plateau between 3 and 5 mg/d with high confidence in the evidence (ED50 = 1.7 mg/d, SMD = 0.27; ED95 = 4.0 mg/d, SMD = 0.52; n = 28; k = 70; N = 6531; median VPC = 30.7%; Wald test p < 0.001). Both the oral formulation and long-acting injection formulations showed a similar curve, although only the relatively lower dose ranges have been examined for some long-acting injection formulations (eAppendix-11).

In children/adolescents, the dose-response curve was hyperbolic reaching plateau between 3 and 5 mg/d with low confidence in the evidence (ED50 = 1.4 mg/d, SMD = 0.41; ED95 = 3.3 mg/d, SMD = 0.78; n = 2; k = 5; N = 413; median VPC = 0%; Wald test p < 0.001).

#### Sertindole

The dose-response curve was hyperbolic reaching plateau between 15 and 20 mg/d with high confidence in the evidence (ED50 = 8.2 mg/d, SMD = 0.25; ED95 = 19.4 mg/d, SMD = 0.47; n = 4; k = 15; N = 1228; median VPC = 0%; Wald test p < 0.001).

#### Xanomeline/trospium

We found no eligible studies.

#### Ziprasidone

The dose-response curve showed monotonic increase among the dose range examined with low confidence in the evidence (range, 0 mg–320 mg; ED50 = 104.9 mg/d, SMD = 0.27; ED95 = 298.1 mg/d, SMD = 0.53; n = 7; k = 22; N = 1345; median VPC = 0.9%; Wald test p < 0.001).

#### Zotepine

We only found one two-arm study of a placebo-controlled trial. The dose examined was 300 mg/d with SMD of 0.88 (95% CI, 0.48–1.28. n = 1; k = 2; N = 106).

#### Pooling all the anti-dopaminergic antipsychotics

**Fig. 4:**
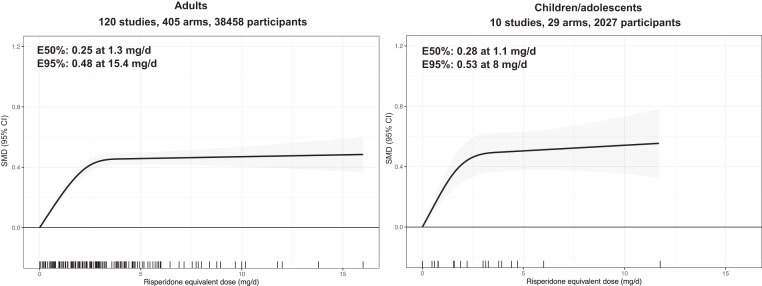
**Dose-response curve across antipsychotic drugs in adults with acute schizophrenia, with doses converted to risperidone equivalents**. The figure shows the dose-response relationship between antipsychotic doses, pooled across different antipsychotics using risperidone dose equivalents, and efficacy compared to placebo (0 mg/day) for overall symptoms in adults and children/adolescents with acute schizophrenia. Findings up to 16 mg/d risperidone dose equivalents are presented. Antipsychotic doses were converted to risperidone dose equivalents (mg/day) using the 95% effective maximum doses (ED95) from the dose-response meta-analysis of individual drugs. Due to sparser data in children/adolescents, risperidone dose equivalents were calculated using the ED95 values derived from adult data. The solid line represents the mean dose-response relationship, and the shaded area shows the 95% confidence intervals. The rug plot displays the dose levels available in the dose-response meta-analysis. E50% and E95% indicate the doses at which an antipsychotic achieves 50% and 95% of the maximum effect size (vs placebo), respectively, as observed in the dose–response meta-analysis. mg/day = milligrams per day (for long-acting injectables, the equivalent oral dose was calculated first).

Fig. 4 shows the pooled dose-response curve after converting doses of individual antipsychotics to risperidone equivalents using the estimated ED95 (Table 1). Although the estimated ED95 of the pooled dose-response curve in adults was 15.4 mg/d, the dose-response relationship plateaued between 3 and 5 mg/d risperidone equivalent and showed no meaningful increases beyond this range (ED50 = 1.3 mg/d, SMD = 0.25; ED95 = 15.4 mg/d, SMD = 0.48; n = 120; k = 405; N = 38,458; median VPC = 40.9%; Wald test p < 0.001) (Fig. 4). The pooled dose-response curve in children/adolescents showed a similar pattern (ED50 = 1.1 mg/d, SMD = 0.28; ED95 = 8.0 mg/d, SMD = 0.53; n = 10; k = 29; N = 2027; median VPC = 13.3%; Wald test p < 0.001) (Fig. 4). Sensitivity analyses using alternative dose equivalency methods did not show materially different results (eAppendix-11).

**Table 1:** Dose ranges (licensed dose range, evidence-based dose, DDD and consensus target dose).

Antipsychotic	Licensed dose range^a^ (mg/day)	Current study		DDD (mg/day)	Consensus target and maximum dose^b^ (mg/day)
		ED50 (mg/day)	ED95 (mg/day)		
Amisulpride	400–1200	264.7	536.7	400	400–800/1000
Aripiprazole	10–30	4.3	10.2	15	10–30/30
Asenapine	10–20	4.5	10.6	20	10–20/20
Blonanserin^c^	8–24	4.2	14.5	NA	NA
Brexpiprazole	2–4	0.7	3.5	3	2–4/4
Cariprazine	1.5–6	2.0	6.2	3	3–6/6
Clozapine	300–900	279.8	567.2	300	200–500/800
Haloperidol	1–100 (USA); 2–20 (EU, UK)	4.2	12.4	8	4.5–10/17.5
Iloperidone	12–24	13.1	22.9	18	12–24/24
Lumateperone	60	14.9	34.8	60	60–60/60
Lurasidone	40–160	32.0	207.5	60	40–120/160
Olanzapine	5–20	4.7	15.7	10	10–20/30
Olanzapine + samidorphan	5–20 (+10)	NA	NA	NA	NA
Paliperidone	3–12	3.0	12.7	6	6–12/12
Quetiapine	150–800	260.8	1021.5	400	300–750/800
Risperidone	4–16	1.7	4.0	5	3–6/8
Sertindole	12–20	8.2	19.4	16	12–20/24
Xanomeline + trospium chloride	200–250	NA	NA	NA	NA
Ziprasidone	20–160	104.9	298.1	80	80–160/160
Zotepine	75–450	NA	NA	200	100–300/400

The findings in adult populations are presented in this table.

DDD = Defined Daily Dose according to the World Health Organization; ED50 = effective dose 50%; ED95 = effective dose 95%; NA = not available.

a Licensed dose range according to Food and Drug Administration (FDA, USA), and if not available to European Medicines Agency (EMA, EU), Medicines and Healthcare products Regulatory Agency (UK), or Pharmaceuticals and Medical Devices Agency (Japan). Due to a discrepancy in the maximum approved dose of haloperidol between the US and EU/UK, both dose ranges are presented.

b Consensus target dose according to the Second International Consensus Study of Antipsychotic Dosing.^3^^,^^26^

c No consensus-based target dose range or DDD was available for blonanserin. We used the licensed dose range for the target dose range and the mean dose delivered (16 mg/d) in flexible-dose arms in clinical trials for presentation in Fig. 2.

## Discussion

To our knowledge, this is the most comprehensive and up-to-date dose-response meta-analyses on the efficacy of antipsychotics in people with acute exacerbations of schizophrenia spectrum disorders. By analyzing a substantially larger sample size, wider dose ranges, and data across age groups, it addresses uncertainties left by previous work^9^ and provides more robust information to support clinical decision-making regarding antipsychotic dosing.

Overall, we found that the dose-response relationships of antipsychotic efficacy are, on average, hyperbolic, reaching a plateau at 3–5 mg/d of risperidone dose equivalents, above which no further advantage in efficacy can generally be expected. This pattern was consistent with earlier analyses,^9^ and appeared broadly similar in children/adolescents and adults. The larger sample size in the current analysis allowed more precise estimates with moderate to high confidence for most individual drugs, showing also hyperbolic dose-response curves that plateaued within the low-to-medium portion of the recommended dose ranges when contextualized against licensed doses and expert consensus recommendations (Table 1, Fig. 2).^3^ Therefore, these findings suggest that maximum recommended doses may still be high, in some cases reaching up to three times the doses associated with near-maximal efficacy, such as for aripiprazole (ED95: 10.2 mg/d; maximum dose, 30 mg/d^3^), indicating that clinical practice guidelines should incorporate such evidence-based information.

Notably, sample sizes increased approximately ten-fold increases for haloperidol (from 1 to 22 studies; 199 to 2740 participants), olanzapine (from 2 to 22 studies; 401 to 4807 participants) and risperidone (from 3 to 28 studies; 664 to 6531 participants) compared to the previous analysis.^9^ With such expanded data, several antipsychotics that previously showed potential monotonic increases at higher doses (e.g., olanzapine, lurasidone, paliperidone, sertindole)^9^ no longer demonstrated such patterns but instead hyperbolic shapes. For example, the earlier meta-analysis indicating increases in efficacy of olanzapine up to 15 mg/d (the maximum dose examined),^9^ while our current analysis examined doses up to 40 mg/d and identified the plateau at doses 10–15 mg/d. The shape of the dose-response curve above 20 mg/d was primarily driven by a dose-finding trial comparing 10 mg, 20 mg and 40 mg of olanzapine,^30^ which found on average similar efficacy between 10, 20 and 40 mg/d.

The findings for haloperidol require interpretation with extreme caution. The previous meta-analysis suggested a plateau at 6.3 mg/d,^9^ whereas the current analysis indicated a plateau between 8 and 12 mg/d. This relatively higher dose range generally aligns with the daily defined dose of haloperidol of 8 mg/d and doses recommended by expert consensus (4.5–10 mg/d).^3^ However, only 22% of participants allocated to haloperidol in the included trials received doses bellow 10 mg/d. As most data derived from higher doses, evidence for the doses below 10 mg/day remains limited, and we downrated the confidence in the evidence to very low (eAppendix-12). This finding also contrasts with molecular imaging studies indicating that 4–5 mg/d of haloperidol typically corresponds to approximately 80% off dopamine D2 receptor occupancy (the upper end of the suggested therapeutic window),^31^^,^^32^ above which greater efficacy is not expected, but the risk of extrapyramidal symptom increases.^6^^,^^31^ We also did not include plasma-level-based clinical trials, which were, generally old and examined higher dose ranges (>10–20 mg/d).^33^, ^34^, ^35^ Considering the above, haloperidol may be initiated at a relatively low dose of around 4 mg/day, with the option of increasing up to approximately 12 mg/day if the clinical response is insufficient, while taking into account the increased risk of extrapyramidal symptoms. Routine use of high doses (≥10 mg/day) is not supported.

Our study has certain limitations that should be considered when interpretating the results.

First, we used a one-stage dose-response meta-analytic model, which has been shown to be more efficient and powerful,^11^ and enabled the inclusion of data from two-arm trials, increasing the sample size for each antipsychotic and yielding more precise estimates of non-linear relationships than the traditional two-stage models previously used.^9^^,^^11^ However, this approach might potentially introduce heterogeneity due to potential differences between trials comparing a single dose with placebo and those comparing multiple doses; excluding such single-dose studies did not materially change the results (eAppendix-11). The findings were also robust to other decisions made to provide a comprehensive analysis, such as combining different formulations and trial durations (eAppendix-11). There was no clear indication of important clinical and methodological heterogeneity, further supported by our strict inclusion criteria and the lack of clear imbalances in potential effect modifiers (eAppendix-13); however, some between-study heterogeneity was observed (eAppendix-9), which led us to downgrade the certainty of the evidence for some drugs (e.g., blonanserin, lumateperone, olanzapine, paliperidone, quetiapine; eAppendix-12).

Second, dose-response relationships for some antipsychotics were uncertain, with low or very low confidence in the evidence, mainly due to risk of bias and imprecise estimates (i.e., amisulpride, blonanserin, cariprazine, clozapine, lumateperone and ziprasidone in adults, and most individual antipsychotics in children/adolescents). For haloperidol, confidence was also very low due to indirectness, as it was examined primarily in higher dose ranges (>10 mg/d). There were also not enough trials to conduct dose-response meta-analyses for olanzapine/samidorphan, xanomeline/trospium and zotepine. More trials are needed to further clarify the dose-response relationship of these antipsychotics.

Third, our analysis provides information on the dose-response relationships for efficacy of antipsychotics for the “average” patients included in the trials, i.e., typically males in their 30–40 s with an acute episode of schizophrenia, chronic illness, and living in the Western countries. Substantial interindividual variability is expected, and some groups, e.g., women and younger/older individuals may require lower doses.^2^^,^^15^^,^^36^ Data were too sparse to conduct separate dose-response meta-analyses for key subgroups, but results were generally similar between children/adolescents and adults, and findings did not materially change in sensitivity analyses excluding subgroups such as first-episode patients or those with treatment resistance, restricting analyses to inpatients, or adjusting for mean baseline severity (eAppendix-11). Individual-participant-data are needed to examine subgroup differences based on age, sex, body composition and baseline severity.^37^ Moreover, several factors influence pharmacokinetics and pharmacodynamics, and subsequently the relationships between dose, plasma levels, and drug effects, including comorbidities, concomitant medications, and CYP450 genotypes.^38^ Such factors are also common reasons for exclusion from trials, meaning that the average participants in the included studies may not fully represent the broader patient population seen in everyday practice, limiting generalizability.^39^ Therefore, dose-response relationships presented here should be supplemented by recommendations on dose adjustments for special subgroups, as outlined in treatment guidelines, e.g., lower starting doses by 25%–50% are recommended in older individuals, especially those with comorbidities.^2^

Fourth, this analysis focused on efficacy, but treatment decisions should balance potential benefits against dose-related side-effects, such as weight-gain,^4^ extrapyramidal side-effects,^5^^,^^6^ and prolactin.^7^ Digital tools that integrate and graphically present efficacy, acceptability, and tolerability data from dose-response meta-analyses may aid in interpreting trade-offs involved in choosing an antipsychotic dose.^40^ People with lived experience were not involved in this study, but their perspectives and preferences should be actively incorporated in future work aimed at evaluating the risk–benefit profile of antipsychotic doses, and should also be integrated into a shared-decision making when selecting an antipsychotic dose.

Last, we updated the estimates of the doses achieving 95% of the maximal efficacy (ED95) that can be used to estimate equivalent doses across antipsychotics (Table 1), within the known limitations of such equivalences as previously discussed in the earlier meta-analysis.^9^ However, ED95s may be overestimated when wide dose ranges are examined due to restricted cubic splines being constrained to be linear beyond the last knot point, as seen in the pooled analysis where the plateau appeared at 3–5 mg/day of risperidone equivalents but the estimated ED95 was 15.4 mg/day (Fig. 4). Therefore, we also presented the doses achieving 50% of the maximal efficacy (ED50), which are typical in the characterization of dose-response relationships.^41^ As the trials included were not primarily designed to evaluate antipsychotic switching between equivalent doses or dose escalation in cases of non-response, for which evidence remains limited,^42^^,^^43^ our findings do not provide direct evidence on these clinical scenarios.

In conclusion, dose-response curves for antipsychotic efficacy in people with an acute exacerbation of schizophrenia spectrum disorders were hyperbolic, with a plateau reached within the lower-to-middle portion of the recommended dose range, and with moderate to high confidence in the evidence. This indicates that, for the average patient, increasing doses beyond this plateau, is unlikely to provide additional efficacy. Our analysis provides estimates of the plateau doses, which can serve as general guidance in clinical practice and should be considered alongside the side-effect profiles to help balance the trade-offs involved in treatment decisions. Although dose-response relationships were broadly similar between children/adolescents and adults, considerable interindividual variation can be expected in clinical practice, and multiple participant characteristics may influence the required dose. These factors should be carefully integrated within a shared decision-making process to support individualized selection of antipsychotic doses.

## Contributors

YF: Conceptualization, Data curation, Investigation, Methodology, Project administration, Validation (access and verification of the data), Writing—original draft, Writing—review & editing.

XL: Data curation, Writing—review & editing.

AR: Conceptualization, Writing—review & editing.

JT: Data curation, Writing—review & editing.

HW: Data curation, Writing—review & editing.

JST: Data curation, Funding acquisition, Resources, Writing—review & editing.

JP: Project administration, Writing—review & editing.

JMD: Conceptualization, Supervision, Writing—review & editing.

SL: Conceptualization, Data curation, Funding acquisition, Methodology, Supervision, Writing—review & editing.

SS: Conceptualization, Data curation, Formal Analysis, Funding acquisition, Investigation, Methodology, Project administration, Software, Validation (access and verification of the data), Visualization, Writing—original draft, Writing—review & editing.

## Data sharing statement

Codes and data are available on reasonable request from the corresponding author.

## Declaration of interests

YF: YF received grant by the SENSHIN Medical Research Foundation.

XL: None.

AR: None.

JT: None.

HW: is currently an employee of Elsevier in the role of senior editor for The Lancet Public Health. Her contribution happened before she was employed by Elsevier and she had no role in the editorial evaluation, peer-review process or decision to accept the article.

JST: None.

JP: In the last three years JP has received grants/contracts from DFG (Deutsche Forschungsgemeinschaft), DZPG (Deutsches Zentrum für Psychische Gesundheit), DZNE (Deutsches Zentrum für Neurodegenerative Erkrankungen), UK DRI (UK Dementia Research Institute), MRC (Medical Research Council); received payment from FOMF, received support for attending meetings from CHDI foundation; patents related to EPO variants; participation on a Data Safety Board or Advisory Board in SINAPPS2; Leadership role in DGPPN (Deutsche Gesellschaft für Psychiatrie und Psychotherapie, Psychosomatik und Nervenheilkunde), DGBP (Deutsche Gesellschaft für Bipolare Störungen), DZPG.

JMD: None.

SL: In the last three years SL has received honoraria for advising/consulting and/or for lectures and/or for educational material from Angelini, Apsen, Bristol-Myers-Squibb, Boehringer-Ingelheim, Gedeon-Richter, Johnson and Johnson, Karuna, Kynexis, Mitsubishi, Neurotorium, NovoNordisk, Orionpharma, Otsuka, ROVI, SunPharma, TEVA.

SS: None.

## Appendix A Supplementary data

eAppendix
