# Supplementary material for: Antipsychotic dose and efficacy for acute schizophrenia spectrum disorders: an updated systematic review and dose-response meta-analysis
Source: eClinicalMedicine. 2026 Mar 26;94:103847. doi: 10.1016/j.eclinm.2026.103847 (PMC13054534; doi:10.1016/j.eclinm.2026.103847)
Supplement: eAppendix [file mmc1.pdf]

SUPPLEMENTARY APPENDIX

Antipsychotic dose and efficacy for acute schizophrenia spectrum disorders: an updated systematic review and dose-response meta-analysis

Yuki Furukawa, Xiao Lin, Alessandro Rodolico, Jing Tian, Hui Wu, Johannes Schneider-Thoma, Josef Priller, John M Davis, Stefan Leucht, Spyridon Siafis

Table of Contents

eAppendix 1: PRISMA 2020 checklist ***3***

eAppendix 2: PROSPERO registration and protocol ***8***

Protocol **8**

Differences between protocol and review **16**

Search strategy **20**

eAppendix 3: Oral dose conversion scheme ***21***

eAppendix 4: PRISMA flow chart ***22***

eAppendix 5: Description of studies without usable data ***24***

eAppendix 6: Description of studies included in analyses ***28***

eAppendix 7: Risk of bias assessment ***56***

eAppendix 8: Summary of statistical results ***62***

Adults **62**

Children/adolescents **64**

eAppendix 9: Heterogeneity assessments ***65***

eAppendix 10: Small-study effects and publication bias ***81***

eAppendix 11: Sensitivity analyses ***84***

Different formulations **84**

Different knot locations **85**

Different subgroups **86**

Other sensitivity analyses **87**

Using different pooling methods **88**

eAppendix 12: GRADE ***89***

Confidence in the evidence for adults **92**

Confidence in the evidence for children/adolescents **94**

eAppendix 13: Distribution plots to examine heterogeneity ***95***

Mean age **95**

Trial duration 96
Proportion of males 97
Mean baseline PANSS score..98
Risk of Bias...99

eAppendix 1: PRISMA 2020 checklist

Section and Topic	Item #	Checklist item	Location where item is reported
**TITLE**			
Title	1	Identify the report as a systematic review.	TITLE
**ABSTRACT**			
Abstract	2	See the PRISMA 2020 for Abstracts checklist.	ABSTRACT
**INTRODUCTION**			
Rationale	3	Describe the rationale for the review in the context of existing knowledge.	INTRODUCTION
Objectives	4	Provide an explicit statement of the objective(s) or question(s) the review addresses.	INTRODUCTION
**METHODS**			
Eligibility criteria	5	Specify the inclusion and exclusion criteria for the review and how studies were grouped for the syntheses.	METHODS/ Eligibility criteria and search strategies
Information sources	6	Specify all databases, registers, websites, organisations, reference lists and other sources searched or consulted to identify studies. Specify the date when each source was last searched or consulted.	METHODS/ Eligibility criteria and search strategies/Search Strategies
Search strategy	7	Present the full search strategies for all databases, registers and websites, including any filters and limits used.	eAppendix2
Selection process	8	Specify the methods used to decide whether a study met the inclusion criteria of the review, including how many reviewers screened each record and each report retrieved, whether they worked independently, and if applicable, details of automation tools used in the process.	METHODS/ Data extraction and risk of bias assessment
Data collection process	9	Specify the methods used to collect data from reports, including how many reviewers collected data from each report, whether they worked independently, any processes for obtaining or confirming data from study investigators, and if applicable, details of automation tools used in the process.	METHODS/ Data extraction and risk of bias assessment
Data items	10a	List and define all outcomes for which data were sought. Specify whether all results that were compatible with each outcome domain in each study were sought (e.g. for all measures, time points, analyses), and if not, the methods used to decide which results to collect.	METHODS/ Eligibility criteria and search strategies/Outcome
	10b	List and define all other variables for which data were sought (e.g. participant and intervention characteristics,	

Section and Topic	Item #	Checklist item	Location where item is reported
		funding sources). Describe any assumptions made about any missing or unclear information.	
Study risk of bias assessment	11	Specify the methods used to assess risk of bias in the included studies, including details of the tool(s) used, how many reviewers assessed each study and whether they worked independently, and if applicable, details of automation tools used in the process.	METHODS/ Data extraction and risk of bias assessment
Effect measures	12	Specify for each outcome the effect measure(s) (e.g. risk ratio, mean difference) used in the synthesis or presentation of results.	METHODS/ Eligibility criteria and search strategies/Outcome
Synthesis methods	13a	Describe the processes used to decide which studies were eligible for each synthesis (e.g. tabulating the study intervention characteristics and comparing against the planned groups for each synthesis (item #5)).	eAppendix6
	13b	Describe any methods required to prepare the data for presentation or synthesis, such as handling of missing summary statistics, or data conversions.	METHODS/ Eligibility criteria and search strategies/Outcome
	13c	Describe any methods used to tabulate or visually display results of individual studies and syntheses.	NA
	13d	Describe any methods used to synthesize results and provide a rationale for the choice(s). If meta-analysis was performed, describe the model(s), method(s) to identify the presence and extent of statistical heterogeneity, and software package(s) used.	METHODS/Data Analysis
	13e	Describe any methods used to explore possible causes of heterogeneity among study results (e.g. subgroup analysis, meta-regression).	METHODS/Data Analysis
	13f	Describe any sensitivity analyses conducted to assess robustness of the synthesized results.	METHODS/Data Analysis
Reporting bias assessment	14	Describe any methods used to assess risk of bias due to missing results in a synthesis (arising from reporting biases).	METHODS/Data Analysis
Certainty assessment	15	Describe any methods used to assess certainty (or confidence) in the body of evidence for an outcome.	METHODS/Data Analysis
**RESULTS**			
Study selection	16a	Describe the results of the search and selection process, from the number of records identified in the search to the number of studies included in the review, ideally using a flow diagram.	RESULTS
	16b	Cite studies that might appear to meet the inclusion criteria, but which were excluded, and explain why they were excluded.	NA
Study characteristics	17	Cite each included study and present its characteristics.	eAppendix6

Section and Topic	Item #	Checklist item	Location where item is reported
Risk of bias in studies	18	Present assessments of risk of bias for each included study.	eAppendix7
Results of individual studies	19	For all outcomes, present, for each study: (a) summary statistics for each group (where appropriate) and (b) an effect estimate and its precision (e.g. confidence/credible interval), ideally using structured tables or plots.	eAppendix6
Results of syntheses	20a	For each synthesis, briefly summarise the characteristics and risk of bias among contributing studies.	NA
	20b	Present results of all statistical syntheses conducted. If meta-analysis was done, present for each the summary estimate and its precision (e.g. confidence/credible interval) and measures of statistical heterogeneity. If comparing groups, describe the direction of the effect.	eAppendix9
	20c	Present results of all investigations of possible causes of heterogeneity among study results.	NA
	20d	Present results of all sensitivity analyses conducted to assess the robustness of the synthesized results.	Figure2
Reporting biases	21	Present assessments of risk of bias due to missing results (arising from reporting biases) for each synthesis assessed.	eAppendix10
Certainty of evidence	22	Present assessments of certainty (or confidence) in the body of evidence for each outcome assessed.	Figure1
**DISCUSSION**			
Discussion	23a	Provide a general interpretation of the results in the context of other evidence.	DISCUSSION
	23b	Discuss any limitations of the evidence included in the review.	DISCUSSION
	23c	Discuss any limitations of the review processes used.	NA
	23d	Discuss implications of the results for practice, policy, and future research.	DISCUSSION
**OTHER INFORMATION**			
Registration and protocol	24a	Provide registration information for the review, including register name and registration number, or state that the review was not registered.	METHODS
	24b	Indicate where the review protocol can be accessed, or state that a protocol was not prepared.	eAppendix2
	24c	Describe and explain any amendments to information provided at registration or in the protocol.	eAppendix2
Support	25	Describe sources of financial or non-financial support for the review, and the role of the funders or sponsors in the review.	Funding
Competing interests	26	Declare any competing interests of review authors.	Competing Interests
Availability of data, code and	27	Report which of the following are publicly available and where they can be found: template data collection forms; data extracted from included studies; data used for all analyses; analytic code; any other materials used in the	Data availability

Section and Topic	Item #	Checklist item	Location where item is reported
other materials		review.	

From: Page MJ, McKenzie JE, Bossuyt PM, Boutron I, Hoffmann TC, Mulrow CD, et al. The PRISMA 2020 statement: an updated guideline for reporting systematic reviews. BMJ 2021;372:n71. doi: 10.1136/bmj.n71
For more information, visit: www.prisma-statement.org.

Section and Topic	Item #	Checklist item	Reported (Yes/No)
**TITLE**			
Title	1	Identify the report as a systematic review.	Yes
**BACKGROUND**			
Objectives	2	Provide an explicit statement of the main objective(s) or question(s) the review addresses.	Yes
**METHODS**			
Eligibility criteria	3	Specify the inclusion and exclusion criteria for the review.	Yes
Information sources	4	Specify the information sources (e.g. databases, registers) used to identify studies and the date when each was last searched.	Yes
Risk of bias	5	Specify the methods used to assess risk of bias in the included studies.	
Synthesis of results	6	Specify the methods used to present and synthesise results.	Yes
**RESULTS**			
Included studies	7	Give the total number of included studies and participants and summarise relevant characteristics of studies.	Yes
Synthesis of results	8	Present results for main outcomes, preferably indicating the number of included studies and participants for each. If meta-analysis was done, report the summary estimate and confidence/credible interval. If comparing groups, indicate the direction of the effect (i.e. which group is favoured).	Yes
**DISCUSSION**			
Limitations of evidence	9	Provide a brief summary of the limitations of the evidence included in the review (e.g. study risk of bias, inconsistency and imprecision).	Yes
Interpretation	10	Provide a general interpretation of the results and important implications.	Yes
**OTHER**			

Section and Topic	Item #	Checklist item	Reported (Yes/No)
Funding	11	Specify the primary source of funding for the review.	Yes
Registration	12	Provide the register name and registration number.	Yes

From: Page MJ, McKenzie JE, Bossuyt PM, Boutron I, Hoffmann TC, Mulrow CD, et al. The PRISMA 2020 statement: an updated guideline for reporting systematic reviews. BMJ 2021;372:n71. doi: 10.1136/bmj.n71. This work is licensed under CC BY 4.0. To view a copy of this license, visit https://creativecommons.org/licenses/by/4.0/

eAppendix 2: PROSPERO registration and protocol

Registration number: CRD42020181467

Available from: https://www.crd.york.ac.uk/prospero/display_record.php?ID=CRD42020181467

As stated below, the protocol pertains to dose-response meta-analyses for the efficacy and side effects of antipsychotics, planned to be published in separate publications. The data extraction for the dose-response meta-analysis of overall efficacy had already started, as also mentioned in the protocol, before submission to PROSPERO, given that it was based on the previously published two-stage dose-response meta-analysis of acute efficacy from our group [Leucht et al., 2020].

Protocol

Citation

Stefan Leucht, Tasnim Hamza, Spyridon Siafis, Hui Wu, Johannes Schneider-Thoma, John Davis. Dose- response meta-analysis of the efficacy and side-effects of antipsychotic drugs in schizophrenia. PROSPERO 2020 CRD42020181467 Available from: https://www.crd.york.ac.uk/prospero/display_record.php?ID=CRD42020181467

Review question

Antipsychotic drugs are efficacious for the treatment of schizophrenia, but they are associated with many side-effects. It is, however, unclear what is the maximally effective dose of each antipsychotic. It is also unclear which side-effects are associated with antipsychotic dose and how these dose versus side-effect relationships look like for each drug. We aim to fill this gap by dose-response meta-analysis of randomized controlled trials. Seven separate publications on overall efficacy and six important side-effects are planned, one for each primary outcome, i.e. 1) overall efficacy, 2) weight gain, 3) extrapyramidal side-effects, 4) prolactin increase, 5) QTc prolongation, 6) sedation, 7) dropouts due to any reason.

This PROSPERO protocol and its registration ID number pertain to the last four planned publications and not to the publications on overall efficacy, weight gain and extrapyramidal side-effects, since data extraction for these outcomes had been started before submission of the protocol. These outcomes are also presented in this protocol, because similar methodology will be followed.

Searches

1. Electronic databases: We will search the Cochrane Schizophrenia Group's Study-Based Register of Trials with the following strategy: (*Amisulpride Dosage* OR *Aripiprazole Dosage* OR *Asenapine Dosage* OR *Brexpiprazole Dosage* OR *Cariprazine Dosage* OR *Clozapine Dosage* OR *Haloperidol Decanoate Dosage* OR *Haloperidol Dosage* OR *Iloperidone Dosage* OR *Lumateperone Dosage* OR

Lurasidone Dosage OR *Olanzapine Dosage* OR *Paliperidone Dosage* OR *Paliperidone Palmitate Dosage* OR *Quetiapine Dosage* OR *Risperidone Dosage* OR *Sertindole Dosage* OR *Ziprasidone Dosage* OR *Zotepine Dosage*) in Pairwise Comparison Field of Study Records.

2. Previous reviews: Our search will be mainly based on our previous dose-response meta-analysis on the efficacy of antipsychotic drugs (Leucht et al. Am J Psychiatry 2020;177:342-53) and on our network meta-analysis about the acute effects of antipsychotics in general (Huhn et al. Lancet 2019;394:939-51). For both reviews exhaustive searches had been undertaken.

3. Reference searching: Reference lists of newly included records will be hand-searched for potentially relevant studies.

4. We will contact authors or pharmaceutical companies for missing data of studies published from 1990 onward. We expect most of the studies have been conducted by pharmaceutical companies who hold the data.

5. There will be no language restriction for the search. Studies from mainland China will be excluded due to quality issues in many of these studies (Tong et al. BMC Med Res Methodol 2018;18:96). Studies conducted in China by international companies will be accepted.

There will be no date/time, language, document type, and publication status limitations. All publications will be selected independently by at least two reviewers. In case of doubt, a third reviewer will be involved. If this procedure does not lead to resolution of the issue, the study authors will be contacted.

Types of study to be included

- Open and blinded RCTs which compared at least two fixed doses of an antipsychotic. Studies which compared one fixed-dose of an antipsychotic with placebo will also be included.

- Only the first phase of cross-over studies will be used to avoid carry-over effects (Elbourne et al. Int J Epidemiol 2002;31:140-9).

- Cluster-randomized-trials will be excluded due to their unit-of-analysis-problems (Whiting-O'Keefe et al. Med Care 1984;22:1101-14).

- Studies with a high risk of bias in terms of randomization according the Cochrane risk of bias tool will be excluded.

- The minimum study duration will be three weeks. There will be no a priori defined maximum duration as long as the patients were acutely ill at the start. The rationale is that special populations, in particular people with predominant negative symptoms, or studies on long-acting injectable medication (LAI) need longer trials.

- Studies in stable patients (study defined) on relapse prevention will be excluded.

Condition or domain being studied

Schizophrenia and schizophrenia-related disorders

Participants/population

- Participants with a diagnosis of schizophrenia or schizophrenia-related disorders, e.g. schizophreniform or schizoaffective disorders, as defined by any criteria, Studies with a maximum of 20% of participants with other diagnoses are allowed.

- Studies in participants with predominant negative symptoms (Krause et al. Eur Arch Psychiatry Clin Neurosci 2018;268:625-39), first-episode of schizophrenia (Oosthuizen et al. Int J Neuropsychopharmacol 2004;7:125-31), in children/adolescents (Krause et al. Eur Neuropsychopharmacol 2018;28:659-74) and in elderly patients (Krause et al. Eur Neuropsychopharmacol 2019;29:1003-22) will be analysed separately, because there is evidence that such patients need lower doses and are more vulnerable for side-effects (Schneider-Thoma et al. Lancet Psychiatry 2019;6:753-65). Studies in participants in treatment-resistant illness (study-defined) will be analysed together with the main group "general people with schizophrenia", because there is no clear evidence that the dose effects of antipsychotics differ based on these characteristics (Samara et al. Cochrane Database Syst Rev 2018;5:CD011883), but they will be excluded in a sensitivity analysis.

- Studies in stable patients (relapse prevention studies) will be excluded. Their inclusion would lead to methodological and clinical heterogeneity (e.g. duration is longer and patients are pretreated).

- No other restriction in terms of setting, gender, nationality and ethnicity.

Intervention(s), exposure(s)

- The following antipsychotics in monotherapy: amisulpride, aripiprazole (oral and depot), asenapine (oral and transdermal), brexpiprazole, cariprazine, clozapine, haloperidol (oral and depot), iloperidone, lumateperone, lurasidone, olanzapine (oral and depot), quetiapine, paliperidone (oral and depot), risperidone (oral and depot), sertindole, ziprasidone, zotepine. This selection comprises all so-called second-generation antipsychotics available in Europe and/or the US. Haloperidol was added as a "gold standard" antipsychotic.

- There will be no restriction in terms of route of administration (except for short-acting injections that are used for acute agitation). Antipsychotic compounds given via different route of administration will be considered as separate compounds. For example, oral aripiprazole, aripiprazole maintena and aripiprazole lauroxil will be considered as three separate antipsychotic interventions. In a similar vein for oral asenapine/transdermal asenapine, oral paliperidone/paliperidone depot once monthly, risperidone/risperidone consta/risperidone RBP-7000, olanzapine/olanzapine depot.

- Immediate and extended release formulations of the same antipsychotic (for example quetiapine) will be considered as the same intervention in the main analysis, but they will be analyzed separately in a sensitivity analysis.

- Fixed-dose schedules, and studies in which patients are randomised to different, narrow fixed dose range, for example olanzapine 5mg/day +/- 2.5 mg/day versus olanzapine 10mg/day +/-2.5mg/day. Flexible-dosing schedules will not be eligible.

Comparator(s)/control

Placebo will be used as the reference in the analyses.

Main outcome(s)

We plan seven separate publications one on overall efficacy and six which focus on different side-effects.

Primary outcomes of each planned review:

1. Overall efficacy:* PANSS total score (Kay and Fiszbein Schizophr Bull 1987;13:261-75) or BPRS (Overall and Gorham Psychol Rep 1962;10:790-812) or if not available any other rating scale on the overall symptoms of schizophrenia.

2. Weight gain:* Weight change (in kg) from baseline to endpoint (continuous outcome). If not available, the mean maximum change from baseline to endpoint will be accepted.

3. Extrapyramidal side effects (EPS):* Mean scores of validated scales to measure EPS. The SAS (Simpson and Angus Acta Psychiatr Scand Suppl 1970;212:11-9) will be preferred to the ESRS (Chouinard Can J Neurol Sci 1980;7:233-44), if available.

4. Mean prolactin increase (in ng/ml)

5. Mean QTc prolongation (in msec)

6. Sedation

7. Drop out due to any reason (all-cause discontinuation). This outcome is a measure of effectiveness, because it comprises dropouts due to adverse events, inefficacy and others.

Outcomes will be measured at study endpoint.

*The PROSPERO protocol does not pertain to the publication of these outcomes, since their data extraction has started before submission of the protocol.

*** Measures of effect**

Weight, prolactin and QTc will be analysed with the mean difference (MD), Efficacy and extrapyramidal sideeffects will be analysed with the standardized mean difference (SMD). The other outcomes are dichotomous outcomes which will be analysed with odds ratios. Also see section ‘strategy for data synthesis’.

For continuous rating scales we will prefer the mean change from baseline to endpoint of these scales over the at endpoint values, if available. Moreover, we will always prefer the broadest scores of rating scales. E.g. for the ESRS scale, we prefer the ESRS total score to the score composed of the subscores of parkinsonism, dystonia and dyskinesia. If those are not available, we will accept the parkinsonism subscore of the ESRS.

Additional outcome(s)

The following secondary outcomes will be analysed in the six separate reviews.

1. Overall efficacy: none.

2. Weight gain:*

- Number of participants with weight (>=7% change from baseline will be preferred, but any study-definition will be eligible, dichotomous outcome).

3. Extrapyramidal side effects (EPS):*

- The number of participants with at least one extrapyramidal side-effect. If not available, the number of participants with 'extrapyramidal disorder' (study defined) will be extracted. If neither of these outcomes is available, the number of participants with increased SAS/ESRS score will be extracted.

- The number of participants who received antiparkinson medication at least once.

- The mean change of the total scores of validated scales to measure akathisia from baseline to endpoint or, if not available, the mean endpoint values of these scales. The Barnes Akathisia Scale (BAS, Barnes Br J Psychiatry 1989;154:672-6) will be preferred to other scales, if available. The BAS total score will be preferred to the BAS global score, because the latter is a more subjective score.

- The number of participants with akathisia. If this outcome is not reported as an adverse event, the number of participants with increased BAS score will be extracted.

4. Prolactin:

- The number of participants with prolactin increase (study defined)

5. QTc prolongation

- The number of participants with QTc prolongation (study defined, but if available > 470msec for women and > 450msec for men)

6. We do not plan secondary outcomes for the reviews on sedation and on drop-out due to any reason.

Outcomes will be measured at study endpoint.

*The PROSPERO protocol does not pertain to the publication of these outcomes, since their data extraction has started before submission of the protocol.

*** Measures of effect**

Except the akathisia scores which will be analyzed using standardized mean differences (SMD), all other outcomes are dichotomous for which odds ratios will be use as measures of effect. Also see section 'strategy for data synthesis'.

Data extraction (selection and coding)

1. Selection of trials: At least two reviewers will independently inspect the titles and abstracts of nonduplicated references identified through the search and will exclude those not pertinent. Discrepancies between the two reviewers will be resolved by discussion reaching consensus. If doubts still remain, the full text will be obtained and eligibility will be assessed. Full texts of included references will be obtained and independently assessed by two reviewers for eligibility. Again, disagreements will be resolved by discussion and, if needed, a third author will be involved. When required, further information will be requested from study authors.

2. Data extraction: Two authors will independently extract data from all selected trials in a Microsoft Access database. When disagreement arises, we will resolve it by discussion and, if needed, involving a third senior author. Where this is not sufficient, we will contact the study authors.

- For continuous outcomes, we will prefer change scores to follow-up data, but we will also accept the latter when the former are not available.

- When authors of original studies used imputation methods to handle missing data, we will prefer them to completers' data. Furthermore, data based on mixed-models of repeated measurement (MMRM), multiple imputation will be preferred over last-observation carried forward (LOCF), if available.

- For dichotomous outcomes, if only completer analyses are presented, we will assume that participants lost to follow-up did not have the outcome. We think that another assumption would overestimate the risk.

- Missing SDs will be calculated from 1) standard error (SE), 2) other measures of variability (95%

confidence intervals, ranges etc), 3) test statistics 4) imputed from the SDs of the other studies using a validated method (Furukawa et al. J Clin Epidemiol 2006;59:7-10) according to the Cochrane Handbook (Higgins and Green 2011).

Risk of bias (quality) assessment

Two independent review authors will assess the risk of bias in the selected studies using the 'Cochrane Collaboration risk of bias' tool. When disagreement arises we will resolve it by discussion and, if needed, involving a third senior author.

Strategy for data synthesis

- The effect sizes for continuous outcomes will be the mean difference (MD), if possible, because this measure can be interpreted more easily by clinicians. To use MD will be possible for weight (in kg), prolactin increase (ng/ml) and QTc prolongation (msec). For other outcomes such as rating scales for overall efficacy or EPS we will use the standardized mean difference (SMD as Hedges' g), because we expect that various EPS scales have been used in the studies. The effect sizes for dichotomous outcomes will be the odds ratio (OR). All effect sizes will be accompanied by their 95% confidence intervals.

- We will conduct a one-stage dose response meta-analysis in a frequentist framework using restricted-cubic splines with the R package 'dosresmeta' developed by (Crippa and Orsini BMC Med Res Methodol 2016;16:91, Crippa et al. Stat Methods Med Res 2019;28:1579-96). We will use knot points at the 25th, 50th and 75th percentile.

- For the outcome overall efficacy we will try to identify the 95% effective doses (ED95) as we did in our previous dose-response meta-analysis (Leucht et al. Am J Psychiatry 2020;177:342-53).

- We will produce absolute dose-response curves: we will synthesize the effects in the placebo arms and we will transform the relative dose-response curves estimated in previous steps to absolute curves.

- For drugs with enough data we will use the Wald statistic to explore whether there is evidence of an overall dose-response relationship and we will report the p-values.

- Small study effects and the possibility of publication bias will be assessed with funnel plots and Egger's test for each antipsychotic, when there are at least 10 studies available.

Analysis of subgroups or subsets

Overall efficacy: We will primarily analyse each antipsychotic separately. As there are no major efficacy differences between antipsychotics, we will also pool all antipsychotics after converting their doses to risperidone equivalents based on two criteria: a) a "scientific" criterion using dose-equivalence based on 95% Effective Doses (Leucht et al. Am J Psychiatry 2020;177:342-53), if a dose-equivalence is not available for a drug the Minimum-Effective-Dose-Method (Leucht et al. Schizophr-Bull 2014;40:314-26, Rothe et al. Schizophr-Res 2018;193:23-8), then Classical-Mean-Dose-Method (Leucht et al. Schizophr-Bull 2015;41:1397-402, Davis Arch-Gen-Psychiatry 1976;33:858-61)), Daily-Defined-Dose-Method (Leucht et al. Schizophr Bull 2016;42 Suppl 1:S90-4) and finally the Delphi conference of the International-Consensus-Study-of-Antipsychotic-Dosing (Gardner et al. Am-J-Psychiatry 2010;167:686-93). b) In a secondary analysis we will convert doses based on "clinical" judgment of experts involved in the International-Consensus-Study-of-Antipsychotic-Dosing (Gardner et al. Am-J-Psychiatry 2010;167:686-93) supplemented by similar judgements by the reviewer team for drugs that were not reported in the consensus statement.

- Side-effects: Antipsychotics differ clearly in their side-effects profiles. Therefore, dose-response analyses will be conducted separately for each antipsychotic drug.

Predefined sensitivity analyses of the primary outcomes will be:

- Exclusion of studies that compared only a single dose of an antipsychotic with placebo. Such studies are no true dose-finding studies. It can be expected that their inclusion will lead to more heterogeneity, because they are different in design.

- Immediate (IR) and extended release (XR) formulations will be analyzed separately (i.e. for quetiapine).

- We will exclude studies in treatment resistant patients (study defined).

- We will exclude open RCTs for subjective outcomes.

Contact details for further information

Stefan Leucht

Stefan.Leucht@tum.de

Organisational affiliation of the review

Department of Psychiatry and Psychotherapy, Technical University Munich, School of Medicine

Review team members and their organisational affiliations

Assistant/Associate Professor Stefan Leucht. Department of Psychiatry and Psychotherapy, Technical

University of Munich, School of Medicine

Ms Tasnim Hamza. Institute of Social and Preventive Medicine, University of Bern

Mr Spyridon Siafis. Department of Psychiatry and Psychotherapy, Technical University, School of Medicine

Dr Hui Wu. Shanghai Jiao Tong University

Dr Johannes Schneider-Thoma. Department of Psychiatry and Psychotherapy, Technical University of

Munich, School of Medicine

Professor John Davis. University of Chicago at Illinois

Type and method of review

Meta-analysis, Systematic review

Anticipated or actual start date

01 March 2020

Anticipated completion date

31 December 2021

Funding sources/sponsors

TH and GS are funded by the European Union's Horizon 2020 research and innovation programme under grant agreement No 825162. HW is funded by the Shanghai General Hospital Excellent Young Medical Talents Project B.

Conflicts of interest

In the last 3 years, Stefan Leucht has received honoraria as a consultant/advisor and/or for lectures from LB Pharma, Otsuka, Lundbeck, Boehringer Ingelheim, LTS Lohmann, Janssen, Johnson&Johnson, TEVA, MSD, Sandoz, SanofiAventis, Angelini, Recordati, Sunovion, Geodon Richter.

Yes

Language

English

Country

China, Germany, Switzerland, United States of America

Stage of review

Review Ongoing

Subject index terms status

Subject indexing assigned by CRD

Subject index terms

MeSH headings have not been applied to this record

Date of registration in PROSPERO

05 July 2020

Date of first submission

22 April 2020

Stage of review at time of this submission

Stage	Started	Completed
Preliminary searches	Yes	No
Piloting of the study selection process	Yes	No
Formal screening of search results against eligibility criteria	Yes	No
Data extraction	No	No
Risk of bias (quality) assessment	No	No
Data analysis	No	No

Differences between protocol and review

- Patients with predominant or prominent negative symptoms: We decided to leave the subgroup of participants with predominantly negative symptoms to another dose-response meta-analysis project on positive and negative symptoms.
- Dose equivalents used to pool antipsychotics: We prespecified in the protocol that we would use a hierarchy of conversion strategies for pooling antipsychotics, but we decided to use only the ED95 conversion strategy. The prespecified strategy was adopted from another dose-response meta-analysis with fewer eligible trials [Leucht et al. 2021]. This change resulted only in dropping a single trial on zotepine with 106 participants and did not change the result. Instead, we conducted sensitivity analyses using alternative methods to estimate dose equivalents, specifically: the 50% effective doses (ED50), minimum effective doses [Leucht et al., 2014], defined daily doses (DDD) [Leucht et al., 2016], consensus-based antipsychotic dosing recommendations [McAdam et al., 2023], as well as the ED50 and ED95 derived from our previously published dose-response meta-analysis [Leucht et al., 2020].
- Estimation of ED50 and ED95: In the protocol, we had stated that we would estimate the 95% effective doses (ED95), but we also estimated and presented the 50% effective doses (ED50) to allow a clearer presentation of the dose-response curve

characteristics, as in the previous dose-response meta-analysis of acute efficacy by our group [Leucht et al., 2020].

- Knot points of the restricted cubic spline: Knot points of the restricted cubic splines to model dose-response relationships were selected using the 25th, 50th, and 75th quantiles of the available doses for the analysis. As the location of the knot points can have an impact on the dose-response curves, we conducted *post-hoc* sensitivity analyses using alternative locations for the knot points, i.e., i) using the 10th, 50th, and 90th quantiles [Harrell. 2015]; ii) setting the knot points according to the expert consensus of antipsychotic dosing at the minimum and maximum of the target doses (see also Table 1) and the median point of the range [McAdam et al., 2023]; iii) 25th, 50th, and 75th quantiles but without considering the placebo (0 mg) dose arms. It should be noted that if the primary selection of the 25th, 50th, and 75th quantiles could not form unique knot points, then the 10th, 50th, and 90th quantiles were used, as in previous analyses, and this was used for asenapine.
- *Post-hoc* sensitivity analysis: We conducted additional *post-hoc* sensitivity analyses to further examine the robustness of the findings by: using the mean dose instead of the target dose, as the latter was preferred in the main analysis but could differ from the actual dose given in the trial; excluding dose arms exceeding the licensed doses, given that these higher doses could have influenced the shape of the dose-response curves; and excluding imputed standard deviations. We also conducted *post-hoc* sensitivity analyses following recommendations by the reviewers during peer review of the manuscript, by restricting the analysis to studies with an overall low risk of bias; excluding studies conducted in outpatient, mixed inpatient/outpatient, or unclear settings; excluding studies with a duration of more than 8 weeks; and performing a sensitivity analysis with a dose-response meta-regression of baseline severity (see below). In addition, as described below, we conducted sensitivity analyses by analyzing separately different drug formulations and excluding first-episode patients.
- Sensitivity analysis for subgroups of patients: Sensitivity analyses were conducted for the studies focusing on adult participants, as data for children/adolescents and other patient subgroups were sparse.
- Dose-response meta-regression with mean baseline severity: For the dose-response meta-regression with baseline severity (*post hoc*), we transformed all BPRS values to PANSS equivalents and excluded outcomes from other symptom scales. Several assumptions were required. First, we assumed that all reported BPRS scores were based on the 18-item version, as this was rarely not explicitly stated, if not otherwise indicated. When studies used a 0-6 response format instead of the standard 1-7 format, we added a constant of 18 for BPRS and 30 for PANSS to the baseline scores. BPRS values were rounded to the nearest integer to allow equipercentile linking to PANSS [Leucht et al, 2013]. We performed drug-specific meta-regressions when ≥10 studies were available (excluding studies with missing baseline severity), estimated dose-response curves at PANSS=90, and tested the effect of baseline severity on the dose-response relationship using Wald tests. No consistent patterns were observed. We repeated the analysis pooling all antipsychotics; two additional studies were excluded because at least one arm lacked baseline severity data. The joint Wald test for baseline-severity effects showed no clear influence on the overall dose-response curve, and the estimated curves were largely comparable to those in the main analysis.

- Dose-response meta-regression with sample size: In addition to funnel plots, we conducted a dose-response meta-regression using the squared root of the sample size to examine small-study effects, as previously conducted [Salanti et al., 2022; Siafis et al., 2023].
- Combining different formulations: We pooled *post-hoc* different formulations of the same antipsychotic in the main analysis but analyzed them separately in a sensitivity analysis.
- Including first-episode patients in the main analysis: We included *post-hoc* first-episode-psychosis studies in the primary analysis [Leucht et al., 2022], but excluded them in a sensitivity analysis.
- Converting dichotomous data on response to continuous data: In order to allow for a more comprehensive synthesis of data, when continuous outcomes were not reported (as in a few studies of haloperidol), we imputed them from dichotomous results by calculating odds ratios and converting them to standardized mean differences using Chinn's approximation, SMD = ln(OR)/1.81, and then to mean differences using the pooled standard deviation from other studies, in order to allow their inclusion with dosresmeta. These data were excluded in a sensitivity analysis of imputed data.
- Adapting and applying the GRADE approach: We evaluated confidence in the evidence by adapting the Grading of Recommendations Assessment, Development and Evaluation (GRADE) framework to dose-response meta-analysis [Guyatt et al., 2008; Siafis et al., 2023; Wu et al., 2023] as in our previous analyses [Siafis et al., 2023], inspired by a prior dose-response meta-analysis [Salanti et al., 2022].
- Distribution of potential effect modifiers across doses: In order to further explore heterogeneity, we examined the distribution of potential effect modifiers across the doses of each drug, in particular mean age (in years), the proportion of male participants, treatment duration (in weeks), mean baseline PANSS total score (estimated as described above for the meta-regression), and overall risk of bias assessment. We conducted this analysis only for studies in adult participants, given the sparser data for children and adolescents. (eAppendix 13)

References

Guyatt GH, Oxman AD, Vist GE, et al. GRADE: an emerging consensus on rating quality of evidence and strength of recommendations. *BMJ.* 2008;336(7650):924-926. doi:10.1136/bmj.39489.470347.AD

Harrell FE. Regression Modeling Strategies. 2015. Springer Cham. https://doi.org/10.1007/978-3-319-19425-7

Leucht S, Rothe P, Davis JM, Engel RR. Equipercentile linking of the BPRS and the PANSS. Eur Neuropsychopharmacol. 2013;23(8):956-959. doi:10.1016/j.euroneuro.2012.11.004

Leucht S, Samara M, Heres S, Patel MX, Woods SW, Davis JM. Dose equivalents for second-generation antipsychotics: the minimum effective dose method. *Schizophr Bull*. 2014;40(2):314-326. doi:10.1093/schbul/sbu001

Leucht S, Samara M, Heres S, Davis JM. Dose Equivalents for Antipsychotic Drugs: The DDD Method. *Schizophr Bull*. 2016;42 Suppl 1(Suppl 1):S90-S94. doi:10.1093/schbul/sbv167

Leucht S, Crippa A, Siafis S, Patel MX, Orsini N, Davis JM. Dose-Response Meta-Analysis of Antipsychotic Drugs for Acute Schizophrenia. *Am J Psychiatry*. 2020;177(4):342-353. doi:10.1176/appi.ajp.2019.19010034

Leucht S, Bauer S, Siafis S, et al. Examination of Dosing of Antipsychotic Drugs for Relapse Prevention in Patients With Stable Schizophrenia: A Meta-analysis. *JAMA Psychiatry*. 2021;78(11):1238-1248. doi:10.1001/jamapsychiatry.2021.2130

Leucht S, Chaimani A, Krause M, et al. The response of subgroups of patients with schizophrenia to different antipsychotic drugs: a systematic review and meta-analysis. *Lancet Psychiatry*. 2022;9(11):884-893. doi:10.1016/S2215-0366(22)00304-2

McAdam MK, Baldessarini RJ, Murphy AL, Gardner DM. Second International Consensus Study of Antipsychotic Dosing (ICSAD-2). *J Psychopharmacol.* 2023;37(10):982-991. doi:10.1177/02698811231205688

Salanti G, Peter N, Tonia T, et al. The Impact of the COVID-19 Pandemic and Associated Control Measures on the Mental Health of the General Population : A Systematic Review and Dose-Response Meta-analysis. *Ann Intern Med*. 2022;175(11):1560-1571. doi:10.7326/M22-1507

Siafis S, Wu H, Wang D, et al. Antipsychotic dose, dopamine D2 receptor occupancy and extrapyramidal side-effects: a systematic review and dose-response meta-analysis. *Mol Psychiatry.* 2023;28(8):3267-3277. doi:10.1038/s41380-023-02203-y

Wu H, Siafis S, Wang D, et al. Antipsychotic-induced akathisia in adults with acute schizophrenia: A systematic review and dose-response meta-analysis. *Eur Neuropsychopharmacol.* 2023;72:40-49. doi:10.1016/j.euroneuro.2023.03.015

Search strategy

We searched the Cochrane Schizophrenia Group's registry until 13.01.2025. Detailed search strategies can be found in the Open Science Framework (https://doi.org/10.17605/OSF.IO/U9R8Z).

In addition, we also conducted a supplementary search in PubMed from 01.01.2025 to 19.01.2026 using the following search strategy:

Search string used for PubMed

("Schizophrenia"[mh] OR schizo*[tiab]) AND ("Aripiprazole"[mh] OR aripiprazole[tiab] OR asenapine[tiab] OR blonanserin[tiab] OR brexpiprazole[tiab] OR cariprazine[tiab] OR "Clozapine"[mh] OR clozapine[tiab] OR "Haloperidol"[mh] OR haloperidol[tiab] OR iloperidone[tiab] OR lumateperone[tiab] OR lurasidone[tiab] OR "Olanzapine"[mh] OR olanzapine[tiab] OR paliperidone[tiab] OR quetiapine[tiab] OR "Risperidone"[mh] OR risperidone[tiab] OR sertindole[tiab] OR ziprasidone[tiab] OR zotepine[tiab] OR xanomeline[tiab] OR KarXT[tiab]) AND (randomized controlled trial[pt] OR controlled clinical trial[pt] OR randomized[tiab] OR placebo[tiab] OR clinical trials as topic[mesh:noexp] OR randomly[tiab] OR trial[ti] NOT (animals[mh] NOT humans [mh])) AND ("2025/01/01"[EDAT] : "3000/01/01"[EDAT])

References

1. Shokraneh, Farhad; Adams, Clive E. Cochrane Schizophrenia Group's Study-Based Register of Randomized Controlled Trials: Development and Content Analysis. Schizophrenia Bulletin Open 2020. https://doi.org/10.1093/schizbullopen/sgaa061.

2. Shokraneh, F., & Adams, C. E. (2024, November 25). Cochrane Schizophrenia Group's Study-Based Register of Randomized Controlled Trials: Development and Content Analysis. https://doi.org/10.17605/OSF.IO/U9R8Z

eAppendix 3: Oral dose conversion scheme

Table 1: Oral dose conversion scheme

Drug formulation	Dose	Oral equivalence
Aripiprazole lauroxil (1)	441 mg/4 weeks	10.71429 mg/d[b]
	882 mg/4 weeks	21.42857 mg/d[b]
Aripiprazole maintena	396.4 mg/4 weeks	14.15714 mg/d[a]
Asenapine HP3070 (2)	3.8 mg/d	10mg/d[b]
	7.6 mg/d	20mg/d[b]
Olanzapine LAI	210 mg/2 weeks	15 mg/d[a]
	300 mg/2 weeks	21.42857 mg/d[a]
	405 mg/4 weeks	14.44643 mg/d[a]
Paliperidone LAI (3)	25 mg/4 weeks	2 mg/d[b]
	50 mg/4 weeks	4 mg/d[b]
	100 mg/4 weeks	8 mg/d[b]
	150 mg/4 weeks	12 mg/d[b]
Risperidone consta	25 mg/2 weeks	1.785714 mg/d[a]
	50 mg/2 weeks	3.571429 mg/d[a]
	75 mg/2 weeks	5.357143 mg/d[a]
Risperidone RBP-7000	90 mg/4 weeks	3.214286 mg/d[a]
	120 mg/4 weeks	4.285714 mg/d[a]
Risperidone ISM	75mg/4 weeks	3mg/d[c]
	100mg/4 weeks	4mg/d[c]

We converted long injectable doses to daily oral equivalences in these ways: [a] dividing the LAI doses by the number of days; [b] according to the published dose equivalences; [c] dose equivalence recommendations from Laboratorios Farmacéuticos ROVI (personal contact).

References

1. Rothe PH, Heres S, Leucht S. Dose equivalents for second generation long-acting injectable antipsychotics: The minimum effective dose method. Schizophrenia research. 2018;193:23-8.
2. Secuado (asenapine) [prescribing information]. Miami, FL: Noven Therapeutics, LLC; 2019.
3. Gopal S, Gassmann-Mayer C, Palumbo J, Samtani M, Shiwach R, Alphs L Practical guidance for dosing and switching paliperidone palmitate treatment in patients with schizophrenia. Current medical research and opinion. 2010;26(2):377-87.

eAppendix 4: PRISMA flow chart

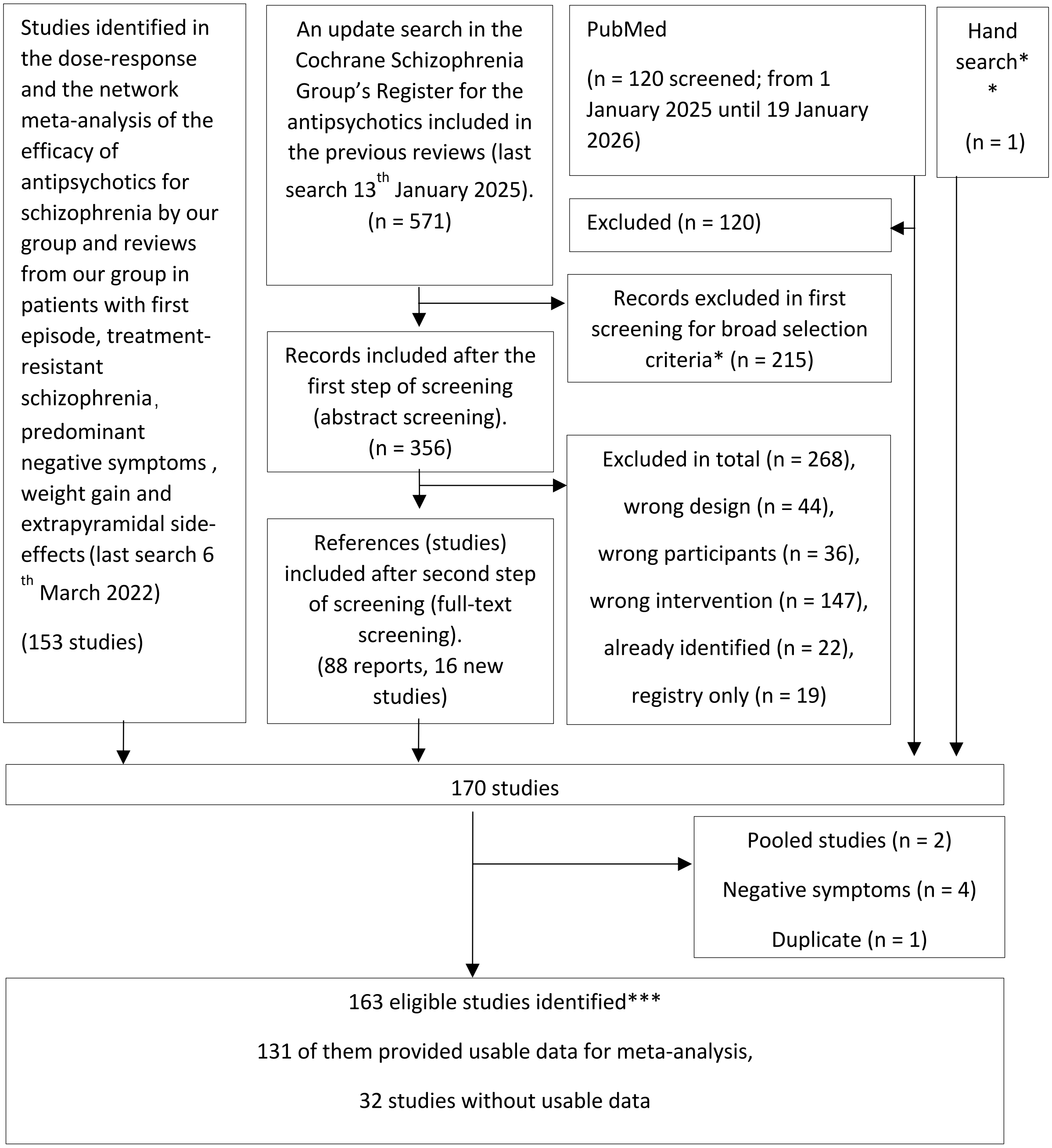

**Broad selection criteria of our group's database: We exclude No RCT (randomized controlled trial) studies, duration less than 3 weeks, population less than 80% schizophrenia, studies conducted and published in China only, no usable comparison (studies investigating combinations of antipsychotics, studies which compared an included antipsychotic drug to another intervention which is not among the list of included antipsychotics or placebo, studies which compare an included antipsychotic to grouped drug groups such as "any FGA"(first generation antipsychotics)).*

*** McEvoy 1991 was added.*

**** 12 studies found in the update search were already included in the previous dose-response meta-analyses of our group. In the update searches additional references to these studies were found.*

References

1. Leucht S, Crippa A, Siafis S, Patel MX, Orsini N, Davis JM. Dose-response meta-analysis of antipsychotic drugs for acute schizophrenia. American Journal of Psychiatry. 2020;177:342-353.

2. Huhn M, Nikolakopoulou A, Schneider-Thoma J, Krause M, Samara M, Peter N, Arndt T, Bäckers L, Rothe P, Cipriani A, Davis J, Salanti G, Leucht S. Comparative efficacy and tolerability of 32 oral antipsychotics for the acute treatment of adults with multi-episode schizophrenia: a systematic review and network meta-analysis. Lancet. 2019;394:939-951.

3. Zhu Y, Li C, Huhn M, Rothe P, Krause M, Bighelli I, et al. How well do patients with a first episode of schizophrenia respond to antipsychotics: A systematic review and meta-analysis. Eur Neuropsychopharmacol. 2017;27(9):835-44.

4. Samara MT, Dold M, Gianatsi M, Nikolakopoulou A, Helfer B, Salanti G, et al. Efficacy, Acceptability, and Tolerability of Antipsychotics in Treatment-Resistant Schizophrenia: A Network Meta-analysis. JAMA Psychiatry. 2016;73(3):199-210.

5. Krause M, Zhu Y, Huhn M, et al. Antipsychotic drugs for patients with schizophrenia and predominant or prominent negative symptoms: a systematic review and meta-analysis. *Eur Arch Psychiatry Clin Neurosci*. 2018;268(7):625-639.

6. Wu H, Siafis S, Wang D, et al. Antipsychotic-induced akathisia in adults with acute schizophrenia: A systematic review and dose-response meta-analysis. *Eur Neuropsychopharmacol*. 2023;72:40-49.

7. Siafis S, Wu H, Wang D, et al. Antipsychotic dose, dopamine D2 receptor occupancy and extrapyramidal side-effects: a systematic review and dose-response meta-analysis. *Mol Psychiatry*. 2023;28(8):3267-3277.

eAppendix 5: Description of studies without usable data

Study	Drug	Dose	Number of participants
Ahmed 2007	Placebo	0.00	150
	Olanzapine	15.00	150
Barnas 2001	Zotepine	150.00	6
	Zotepine	300.00	6
CTRI-2014-04-004521	Lurasidone	40.00	
	Lurasidone	80.00	
	Quetiapine	200.00	
Cetin 1999	Risperidone	2.00	10
	Risperidone	4.00	10
	Risperidone	6.00	10
	Risperidone	8.00	10
	Risperidone	10.00	10
	Haloperidol	20.00	20
Garry 1962b	Placebo	0.00	26
	Haloperidol	3.75	26
ILPB303	Placebo	0.00	
	Iloperidone	8.00	
Ibrahim 2007	Quetiapine		5
	Quetiapine		5
Kato 2012	Lurasidone	20.00	71
	Lurasidone	40.00	72
	Lurasidone	80.00	65
Marinkovic 2006	Risperidone	3.00	15
	Risperidone	4.50	15
	Risperidone	6.00	15

Study	Drug	Dose	Number of particip ants
McGorry 1997	Risperidone	2.00	8
	Risperidone	4.00	9
NCT00468533	Aripiprazole	15.00	16
	Aripiprazole	30.00	16
NCT00653406	Risperidone	25.00	
	Risperidone	37.50	
	Risperidone	50.00	
NCT01625897	Risperidone	12.00	42
	Cariprazine		
	Cariprazine		
NCT03817502	Placebo	0.00	
	Cariprazine	1.50	
	Cariprazine	4.50	
NCT04624243	Placebo	0.00	89
	Risperidone	6.00	45
NCT05168007	Placebo	0.00	
	Cariprazine	3.00	
	Cariprazine	6.00	
NCT05368558	Placebo	0.00	
	Cariprazine		
Palao 1994	Haloperidol	10.00	6
	Haloperidol	20.00	8
	Haloperidol	30.00	8
Pfizer 2008	Placebo	0.00	
	Aripiprazole	15.00	
Potkin 1985	Haloperidol		
	Haloperidol		

Study	Drug	Dose	Number of participants
Potkin 1993	Clozapine	400.00	30
	Clozapine	800.00	28
Potkin 2001	Placebo	0.00	110
	Haloperidol	10.00	56
Santos 1989	Haloperidol	10.00	10
	Haloperidol	15.00	10
	Haloperidol	30.00	10
Smith 1984	Haloperidol	7.50	
	Haloperidol	10.00	
	Haloperidol	25.00	
	Haloperidol	40.00	
Smith 1987	Haloperidol	8.00	12
	Haloperidol	40.00	12
Study 041-002	Placebo	0.00	61
	Asenapine	0.40	60
	Asenapine	0.80	59
	Asenapine	1.60	61
	Risperidone	6.00	61
Study 041-013	Placebo	0.00	64
	Asenapine	3.20	58
	Asenapine	4.80	61
Study 128-301 1997	Haloperidol	10.00	120
	Haloperidol	20.00	118
	Ziprasidone	40.00	116
	Ziprasidone	120.00	115
	Ziprasidone	200.00	128
Swanson 2005	Placebo	0.00	

Study	Drug	Dose	Number of particip ants
	Olanzapine	15.00	
Uzun 2002	Ziprasidone	40.00	
	Ziprasidone	60.00	
	Ziprasidone	80.00	
	Risperidone		
Vichaya 1971	Placebo	0.00	15
	Haloperidol	4.50	15
Winter 1984	Haloperidol	16.00	20
	Haloperidol	80.00	20

eAppendix 6: Description of studies included in analyses

Study name	Diagnostic criteria	Duration (weeks)	In/outpatient	Risk of Bias	Drug	Dose	Dose unit	Number of participants	Mean age (years)	Female participants (%)
Arvanitis 1997	DSM-III-R	6	inpatient	Low	Placebo	0	mg/d	51	36	20
					Haloperidol	12	mg/d	50	37	19
					Quetiapine_IR	75	mg/d	52	37	26
					Quetiapine_IR	150	mg/d	48	38	19
					Quetiapine_IR	300	mg/d	51	38	29
					Quetiapine_IR	600	mg/d	51	39	25
					Quetiapine_IR	750	mg/d	53	35	30
Barbato 2007a	DSM-IV	6	NA	Unclear	Placebo	0	mg/d	145	39	
					Olanzapine	15	mg/d	148	39	24
Barbato 2007b	DSM-IV	6	NA	Unclear	Placebo	0	mg/d	51	34.5	
					Haloperidol	10	mg/d	50	34.5	42
Beasley 1996a	DSM-III-R	6	inpatient	Low	Placebo	0	mg/d	49	36	34
					Olanzapine	1	mg/d	51	38	23
					Olanzapine	10	mg/d	49	39	26
Beasley 1996b	DSM-III-R	6	inpatient	Low	Placebo	0	mg/d	62	35	9
					Olanzapine	5	mg/d	63	35.7	8
					Olanzapine	10	mg/d	62	37.3	12
					Haloperidol	15	mg/d	68	36	10
					Olanzapine	15	mg/d	65	35.9	22
Beasley 1997	DSM-III-R	6	inpatient	Low	Olanzapine	1	mg/d	83	34	34
					Olanzapine	5	mg/d	85	34	34
					Olanzapine	10	mg/d	83	36	36

Study name	Diagnostic criteria	Duration (weeks)	In/outpatient	Risk of Bias	Drug	Dose	Dose unit	Number of participants	Mean age (years)	Female participants (%)
					Haloperidol	15	mg/d	79	36	41
					Olanzapine	15	mg/d	85	37	36
Berger 2008	DSM-IV	4	inpatient	Low	Quetiapine_IR	200	mg/d	46	19.7	29
					Quetiapine_IR	400	mg/d	45	19	36
Bugarski-Kirola 2014	DSM-IV	4	inpatient	Unclear	Placebo	0	mg/d	79	37.8	28
					Olanzapine	15	mg/d	61	40.3	26
Cantillon 2014	DSM-IV-TR	4	NA	Unclear	Placebo	0	mg/d	37	36	29
					Aripiprazole	15	mg/d	19	35	10
Canuso 2010	DSM-IV	6	inpatient	Low	Placebo	0	mg/d	107	37.1	37
					Paliperidone	6	mg/d	105	38.1	33
					Paliperidone	12	mg/d	98	36.5	35
Casey 2008	DSM-IV-TR	6	inpatient	Unclear	Placebo	0	mg/d	114	40.8	23
					Risperidone	6	mg/d	116	41.1	19
Chouinard 1993	DSM-III-R	8	inpatient	Low	Placebo	0	mg/d	22		
					Risperidone	2	mg/d	24		
					Risperidone	6	mg/d	22		
					Risperidone	10	mg/d	22		
					Risperidone	16	mg/d	24		
					Haloperidol	20	mg/d	21		
Citrome 2021	DSM-5	6	inpatient	Unclear	Placebo	0	mg/d	203	42.1	36
					Asenapine_transdermal	4	mg/d	201	41.5	36
					Asenapine_transdermal	8	mg/d	203	42.3	46
Cooper 2000	DSM-III-R	8	mixed	Low	Placebo	0	mg/d	53	36.3	30

Study name	Diagnostic criteria	Duration (weeks)	In/outpatient	Risk of Bias	Drug	Dose	Dose unit	Number of participants	Mean age (years)	Female participants (%)
					Zotepine	300	mg/d	53	39.6	30
Coppola 2011	DSM-IV	6	inpatient	Low	Placebo	0	mg/d	63	36.5	28
					Paliperidone	2	mg/d	66	41.3	24
					Paliperidone	6	mg/d	70	40.3	33
Correll 2015	DSM-IV-TR	6	inpatient	Unclear	Placebo	0	mg/d	178	39.7	36
					Brexpiprazole	0.25	mg/d	87	40.5	32
					Brexpiprazole	2	mg/d	180	39.6	39
					Brexpiprazole	4	mg/d	178	40.8	38
Correll 2020a	DSM-5	4	inpatient	Low	Placebo	0	mg/d	141	41.4	17
					Lumateperone	40	mg/d	146	43.5	25
					Lumateperone	60	mg/d	148	42.4	27
Correll 2020b	DSM-5	12	mixed	Low	Placebo	0	mg/d	132	40.5	33
					Risperidone_ism	75	mg/d	129	42.5	32
					Risperidone_ism	100	mg/d	129	42.9	34
Corrigan 2004	DSM-IV	6	inpatient	Unclear	Placebo	0	mg/d	85	37.2	28
					Olanzapine	15	mg/d	93	36.8	37
Cutler 2006	DSM-IV	6	inpatient	Unclear	Placebo	0	mg/d	86	42.9	18
					Aripiprazole	2	mg/d	92	40.7	20
					Aripiprazole	5	mg/d	90	40.9	24
					Aripiprazole	10	mg/d	94	40	23
Cutler 2008a	DSM-IV	4	mixed	Unclear	Placebo	0	mg/d	140	40.7	23
					Iloperidone	24	mg/d	283	39.5	17
					Ziprasidone	160	mg/d	144	40	24
Cutler 2008b	DSM-IV	6	inpatient	Low	Placebo	0	mg/d	111	42.5	31
					Quetiapine_XR	400	mg/d	113	42.1	30

Study name	Diagnostic criteria	Duration (weeks)	In/outpatient	Risk of Bias	Drug	Dose	Dose unit	Number of participants	Mean age (years)	Female participants (%)
					Quetiapine_XR	600	mg/d	101	41.2	19
					Quetiapine_IR	800	mg/d	109	40.8	37
					Quetiapine_XR	800	mg/d	110	40.2	25
Daniel 1999	DSM-III-R	6	inpatient	Unclear	Placebo	0	mg/d	91	37.2	32
					Ziprasidone	80	mg/d	104	36.8	29
					Ziprasidone	160	mg/d	103	35.8	26
Davidson 2007	DSM-IV	6	inpatient	Low	Placebo	0	mg/d	120	37.3	31
					Paliperidone	3	mg/d	123	36.3	37
					Paliperidone	9	mg/d	123	36.2	36
					Olanzapine	10	mg/d	126	36.5	24
					Paliperidone	15	mg/d	113	37.6	35
Downing 2014	DSM-IV	6	inpatient	Low	Placebo	0	mg/d	253	39.8	39
					Risperidone	4	mg/d	124	40.3	39
Durgam 2014	DSM-IV-TR	6	inpatient	Unclear	Placebo	0	mg/d	148	36	33
					Cariprazine	2	mg/d	140	36.8	36
					Cariprazine	3	mg/d	140	37.1	27
					Risperidone	4	mg/d	138	36.5	30
					Cariprazine	4	mg/d	145	35.8	30
Durgam 2015	DSM-IV-TR	6	inpatient	Unclear	Placebo	0	mg/d	149	38.2	37
					Cariprazine	3	mg/d	151	37.9	36
					Cariprazine	6	mg/d	154	38.6	36
					Aripiprazole	10	mg/d	150	39.3	38
Durgam 2016	DSM-IV-TR	6	inpatient	Unclear	Placebo	0	mg/d	126	41.1	21
					Cariprazine	4	mg/d	122	40.3	18

Study name	Diagnostic criteria	Duration (weeks)	In/outpatient	Risk of Bias	Drug	Dose	Dose unit	Number of participants	Mean age (years)	Female participants (%)
					Cariprazine	12	mg/d	129	42.4	25
Egan 2013	DSM-IV-TR	4	inpatient	Low	Placebo	0	mg/d	78	36.4	36
					Olanzapine	15	mg/d	45	36.1	49
Fabre 1995	DSM-III-R	3	inpatient	Unclear	Placebo	0	mg/d	4	35	0
					Quetiapine_IR	250	mg/d	8	34	0
Findling 2008	DSM-IV	6	mixed	Unclear	Placebo	0	mg/d	98	15.4	39
					Aripiprazole	10	mg/d	99	15.6	55
					Aripiprazole	30	mg/d	97	15.4	36
Findling 2012	DSM-IV-TR	6	mixed	Low	Placebo	0	mg/d	73	15.3	42
					Quetiapine_IR	400	mg/d	73	15.4	41
					Quetiapine_IR	800	mg/d	74	15.4	41
Findling 2015	DSM-IV-TR	8	NA	Low	Placebo	0	mg/d	77	15.4	39
					Asenapine	5	mg/d	72	15.2	37
					Asenapine	10	mg/d	79	15.4	37
Garcia 2009	DSM-IV-TR	6	inpatient	Unclear	Placebo	0	mg/d	61	38.6	38
					Blonanserin	2	mg/d	60	38.3	46
					Blonanserin	5	mg/d	57	37	31
					Blonanserin	10	mg/d	62	38.4	55
					Haloperidol	10	mg/d	58	38.1	42
Goff 1998	DSM-III-R	4	inpatient	Unclear	Ziprasidone	4	mg/d	19	41.7	11
					Ziprasidone	10	mg/d	17	39.2	6
					Haloperidol	15	mg/d	17	35.5	6
					Ziprasidone	40	mg/d	17	38.1	6
					Ziprasidone	160	mg/d	20	41.7	5
Goff 2013	DSM-IV	8	mixed	Unclear	Ziprasidone	160	mg/d	21	41	32

Study name	Diagnostic criteria	Duration (weeks)	In/outpatient	Risk of Bias	Drug	Dose	Dose unit	Number of participants	Mean age (years)	Female participants (%)
					Ziprasidone	320	mg/d	21	39.2	29
Goldman 2017	DSM-IV-TR	6	inpatient	Low	Placebo	0	mg/d	112	15.3	63
					Lurasidone	40	mg/d	108	15.5	61
					Lurasidone	80	mg/d	106	15.3	67
Gopal 2010	DSM-IV	13	inpatient	Low	Placebo	0	mg/4w	132	41	29
					Paliperidone_LAI1M	50	mg/4w	93	39	30
					Paliperidone_LAI1M	100	mg/4w	94	39	35
					Paliperidone_LAI1M	150	mg/4w	30	41	27
Haas 2009a	DSM-IV	6	inpatient	Low	Placebo	0	mg/d	54	15.5	35
					Risperidone	3	mg/d	54	15.7	45
					Risperidone	6	mg/d	50	15.7	27
Haas 2009b	DSM-IV	8	inpatient	Unclear	Risperidone	0.6	mg/d	131	15.6	39
					Risperidone	6	mg/d	124	15.6	48
Hale 2000	DSM-III-R	8	inpatient	Low	Sertindole	8	mg/d	116	34.2	28
					Haloperidol	10	mg/d	123	36.5	32
					Sertindole	16	mg/d	120	34.3	30
					Sertindole	20	mg/d	121	35.2	40
					Sertindole	24	mg/d	115	35	33
Heinrich 1994	ICD-9	4	inpatient	Unclear	Risperidone	4	mg/d	20	32.5	55
					Risperidone	8	mg/d	20	29	45
					Clozapine	400	mg/d	20	38	40
Hera 041-021	DSM-IV	6	inpatient	High	Placebo	0	mg/d	106	39.5	42
					Asenapine	10	mg/d	106	40.4	26

Study name	Diagnostic criteria	Duration (weeks)	In/outpatient	Risk of Bias	Drug	Dose	Dose unit	Number of participants	Mean age (years)	Female participants (%)
					Olanzapine	15	mg/d	103	39.7	22
					Asenapine	20	mg/d	102	41.2	29
Higuchi 2019a	DSM-IV-TR	6	inpatient	Low	Placebo	0	mg/d	148	42.6	41
					Lurasidone	40	mg/d	148	42.1	45
					Lurasidone	80	mg/d	154	43.6	47
Higuchi 2019b	DSM-IV	6	inpatient	High	Placebo	0	mg/d	129	46	44
					Risperidone	4	mg/d	64	44.8	45
					Lurasidone	40	mg/d	125	45.6	40
					Lurasidone	80	mg/d	129	45.7	36
Hirayasu 2010	DSM-IV	6	mixed	Unclear	Placebo	0	mg/d	138	46.2	48
					Paliperidone	6	mg/d	134	44	48
					Olanzapine	10	mg/d	46	46.2	59
Honer 2010	DSM-IV	8	mixed	Low	Quetiapine_IR	800	mg/d	43	37.9	26
					Quetiapine_IR	1,200.00	mg/d	88	40.6	34
ILPB202	DSM-III-R	4.14	NA	Unclear	Placebo	0	mg/d	31		
					Iloperidone	4	mg/d	32		
					Iloperidone	8	mg/d	28		
Ishigooka 2018	DSM-IV-TR	6	inpatient	Low	Placebo	0	mg/d	113	45	56
					Brexpiprazole	1	mg/d	112	44.7	56
					Brexpiprazole	2	mg/d	113	43.3	47
					Brexpiprazole	4	mg/d	109	44.1	51
Iwata 2019	DSM-V	6	inpatient	Low	Placebo	0	mg/d	190	41.5	41
					Blonanserin_transdermal	40	mg/d	194	40.7	41

Study name	Diagnostic criteria	Duration (weeks)	In/outpatient	Risk of Bias	Drug	Dose	Dose unit	Number of participants	Mean age (years)	Female participants (%)
					Blonanserin_transdermal	80	mg/d	193	40.6	41
Iyo 2021	DSM-IV-TR	6	mixed	Low	Placebo	0	mg/d	233	39.3	49
					Lurasidone	40	mg/d	245	41	51
Kahn 2007	DSM-IV	6	mixed	Low	Placebo	0	mg/d	115	34.1	42
					Quetiapine_IR	400	mg/d	119	34.4	42
					Quetiapine_XR	400	mg/d	111	34.1	30
					Quetiapine_XR	600	mg/d	111	34.2	45
					Quetiapine_XR	800	mg/d	117	34.4	40
Kane 2002	DSM-IV	4	inpatient	Unclear	Placebo	0	mg/d	106	38.5	30
					Haloperidol	10	mg/d	104	38.9	35
					Aripiprazole	15	mg/d	102	37.8	25
					Aripiprazole	30	mg/d	102	39.3	31
Kane 2003	DSM-IV	12	mixed	Low	Placebo	0	mg/2w	92	37.7	18
					Risperidone_consta	25	mg/2w	93	38.9	31
					Risperidone_consta	50	mg/2w	98	36.2	18
					Risperidone_consta	75	mg/2w	87	38.1	32
Kane 2007	DSM-IV	6	mixed	Low	Placebo	0	mg/d	126	37.9	48
					Paliperidone	6	mg/d	123	37	50
					Paliperidone	9	mg/d	122	38.5	41
					Olanzapine	10	mg/d	128	36.3	53
					Paliperidone	12	mg/d	129	36	47
Kane 2010	DSM-IV-TR	6	inpatient	Unclear	Placebo	0	mg/d	122	38.5	48
					Haloperidol	8	mg/d	112	38.5	46

Study name	Diagnostic criteria	Duration (weeks)	In/outpatient	Risk of Bias	Drug	Dose	Dose unit	Number of participants	Mean age (years)	Female participants (%)
					Asenapine	10	mg/d	109	38	32
					Asenapine	20	mg/d	105	37.1	37
Kane 2014	DSM-IV-TR	12	inpatient	Low	Placebo	0	mg/4w	167	42.7	19
					Aripiprazole_maintena	400	mg/4w	162	42.1	23
Kane 2015a	DSM-IV-TR	6	inpatient	Low	Placebo	0	mg/d	180	39.3	40
					Brexpiprazole	1	mg/d	117	39.1	36
					Brexpiprazole	2	mg/d	179	36.9	34
					Brexpiprazole	4	mg/d	181	38.6	39
Kane 2015b	DSM-IV-TR	6	inpatient	Unclear	Placebo	0	mg/d	145	36.7	25
					Cariprazine	6	mg/d	147	36.6	22
					Cariprazine	9	mg/d	147	35.5	24
Keck 1998	DSM-III-R	4	inpatient	Unclear	Placebo	0	mg/d	47	39	15
					Ziprasidone	40	mg/d	43	40.5	32
					Ziprasidone	120	mg/d	41	38.8	17
King 1998	DSM-III-R	6	inpatient	Low	Quetiapine_IR	50	mg/d	209	37	32
					Quetiapine_IR	450	mg/d	409	35	34
Kinon 2006	DSM-IV	8	mixed	Low	Olanzapine	10	mg/d	199	41.2	33
					Olanzapine	20	mg/d	200	40.9	32
					Olanzapine	40	mg/d	200	41.6	30
Kinon 2011	DSM-IV	4	inpatient	Unclear	Placebo	0	mg/d	122	38.9	43
					Olanzapine	15	mg/d	62	41.7	45
Kinoshita 2016	DSM-IV-TR	6	inpatient	Low	Placebo	0	mg/d	174	41.1	53
					Asenapine	10	mg/d	173	41.4	57
					Asenapine	20	mg/d	178	41.7	45

Study name	Diagnostic criteria	Duration (weeks)	In/outpatient	Risk of Bias	Drug	Dose	Dose unit	Number of participants	Mean age (years)	Female participants (%)
Klieser 1989	DSM-III	3	inpatient	Unclear	Placebo	0	mg/d	16	42.6	
					Haloperidol	20	mg/d	20	42.6	
Kramer 2010	DSM-IV	9	inpatient	High	Placebo	0	mg/4w	66	40	41
					Paliperidone_LAI1M	50	mg/4w	63	40	35
					Paliperidone_LAI1M	100	mg/4w	68	37	38
Landbloom 2016	DSM-IV-TR	6	mixed	Low	Placebo	0	mg/d	99	41.4	47
					Asenapine	5	mg/d	96	41.5	40
					Asenapine	10	mg/d	111	39.1	39
					Olanzapine	15	mg/d	45	40.8	39
Lane 2001	DSM-IV	6	inpatient	Unclear	Risperidone	3	mg/d	11	30.8	
					Risperidone	6	mg/d	12	30.8	
Lauriello 2008	DSM-IV or DSM-IV-TR	8	inpatient	Low	Placebo	0	mg/2w	98	42.6	38
					Olanzapine_LAI	202	mg/2w	100	39.5	27
					Olanzapine_LAI	210	mg/2w	106	39.8	25
					Olanzapine_LAI	300	mg/2w	98	41.5	28
Liebermann 2015	DSM-IV-TR	4	inpatient	Low	Placebo	0	mg/d	80	40.5	24
					Risperidone	4	mg/d	75	40.7	11
					Lumateperone	60	mg/d	76	38.3	21
					Lumateperone	120	mg/d	80	41.1	13
Lindenmayer 2008	DSM-IV	6	inpatient	Low	Placebo	0	mg/d	78	38.4	23
					Quetiapine_IR	300	mg/d	85	39.8	24
					Quetiapine_XR	300	mg/d	83	39.1	26

Study name	Diagnostic criteria	Duration (weeks)	In/outpatient	Risk of Bias	Drug	Dose	Dose unit	Number of participants	Mean age (years)	Female participants (%)
					Quetiapine_IR	600	mg/d	80	40.6	27
					Quetiapine_XR	600	mg/d	87	38.9	28
					Quetiapine_XR	800	mg/d	85	37.8	19
Lindenmayer 2011	DSM-IV-R	8	inpatient	Unclear	Quetiapine_IR	600	mg/d	31	41	13
					Quetiapine_IR	1,200.00	mg/d	29	39.3	3
Litman 2016	DSM-IV	4	mixed	Low	Placebo	0	mg/d	53	36.9	20
					Risperidone	4	mg/d	30	39	16
Litman 2014	DSM-IV	4	mixed	Low	Placebo	0	mg/d	41	40.2	5
					Olanzapine	15	mg/d	22	35.3	0
Loebel 2013	DSM-IV-TR	6	inpatient	Low	Placebo	0	mg/d	120	37.4	36
					Lurasidone	80	mg/d	125	36.2	23
					Lurasidone	160	mg/d	121	37.9	32
					Quetiapine_XR	600	mg/d	116	37.4	35
Loebel 2015	DSM-IV-TR	6	inpatient	Low	Placebo	0	mg/d	112	40.7	30
					Lurasidone	20	mg/d	101	41.5	36
					Lurasidone	160	mg/d	198	40.5	40
Louza 1988	RDC and DSM-III	6	inpatient	High	Haloperidol	10	mg/d	7	32.6	
					Haloperidol	27	mg/d	6	32.6	
Marder 1994	DSM-III-R	8	inpatient	Low	Placebo	0	mg/d	64	37.1	14
					Risperidone	2	mg/d	63	39.3	14
					Risperidone	6	mg/d	63	37.5	14
					Risperidone	10	mg/d	63	36.2	6
					Risperidone	16	mg/d	61	36.5	17
					Haloperidol	20	mg/d	64	38	9

Study name	Diagnostic criteria	Duration (weeks)	In/outpatient	Risk of Bias	Drug	Dose	Dose unit	Number of participants	Mean age (years)	Female participants (%)
Marder 2007c	DSM-IV	6	inpatient	Low	Placebo	0	mg/d	105	42.3	22
					Paliperidone	6	mg/d	110	42.1	32
					Olanzapine	10	mg/d	105	40.5	20
					Paliperidone	12	mg/d	111	41.4	31
Matsumoto 2018	DSM-IV-TR	6	mixed	Low	Aripiprazole	2	mg/d	34	15.1	60
					Aripiprazole	12	mg/d	30	15.4	37
					Aripiprazole	30	mg/d	41	14.7	56
McEvoy 1991	Reseaech Diagnostic Criteria	5	inpatient	Unclear	Haloperidol	5	mg/d	48	31.5	46
					Haloperidol	12	mg/d	47	31.5	
McEvoy 2007	DSM-IV	6	inpatient	Low	Placebo	0	mg/d	107	41.2	23
					Aripiprazole	10	mg/d	103	40	23
					Aripiprazole	15	mg/d	103	40	25
					Aripiprazole	20	mg/d	97	40.4	18
Meltzer 2004	DSM-IV	6	inpatient	Unclear	Placebo	0	mg/d	96	37.4	24
					Haloperidol	10	mg/d	95	36	30
Meltzer 2007	DSM-IV-TR	6	inpatient	Unclear	Placebo	0	mg/d	144	38.2	26
					Risperidone	6	mg/d	151	38.3	27
Meltzer 2011	DSM-IV	6	inpatient	Low	Placebo	0	mg/d	114	37	23
					Olanzapine	15	mg/d	121	38.3	22
					Lurasidone	40	mg/d	118	37.7	22
					Lurasidone	120	mg/d	118	37.9	21
Meltzer 2012	DSM-IV	6	mixed	Unclear	Haloperidol	2	mg/d	77	40.7	30
					Risperidone	2	mg/d	77	39.3	32

Study name	Diagnostic criteria	Duration (weeks)	In/outpatient	Risk of Bias	Drug	Dose	Dose unit	Number of participants	Mean age (years)	Female participants (%)
					Risperidone	6	mg/d	76	39.4	32
Meltzer 2014	DSM-IV-TR	24	mixed	Unclear	Risperidone_consta	50	mg/2w	82	39.2	27
					Risperidone_consta	100	mg/2w	78	41	28
Meltzer 2015	DSM-IV-TR	12	inpatient	Low	Placebo	0	mg/4w	196	39.5	33
					Aripiprazole_lauroxil	441	mg/4w	196	39.9	32
					Aripiprazole_lauroxil	882	mg/4w	204	39.7	31
Meltzer 2020	DSM-IV-TR	24	outpatient	Low	Lurasidone	80	mg/d	34	47	59
					Lurasidone	240	mg/d	33	45.2	55
Merlo 2000	DSM-IV	8	NA	Unclear	Risperidone	2	mg/d	23	23.2	39
					Risperidone	4	mg/d	26	26	50
NCT00563706	DSM-IV-TR	4	inpatient	Low	Placebo	0	mg/d	21	42	22
					Risperidone	4	mg/d	32	42.7	23
NCT00905307	DSM-IV-TR	6	inpatient	Low	Placebo	0	mg/d	93	38.8	39
					Brexpiprazole	0.25	mg/d	41	40.4	36
					Brexpiprazole	1	mg/d	88	39.2	40
					Brexpiprazole	2.5	mg/d	90	37.4	33
					Brexpiprazole	5	mg/d	92	39.5	41
					Aripiprazole	15	mg/d	50	40.8	32
NCT01625000	DSM-IV-TR	6	NA	Unclear	Placebo	0	mg/d	71	40.1	52
					Cariprazine	3	mg/d	78	40.6	48
					Risperidone	4	mg/d	36	39.5	45
					Cariprazine	6	mg/d	80	40.2	45
					Cariprazine	9	mg/d	36	43	51

Study name	Diagnostic criteria	Duration (weeks)	In/outpatient	Risk of Bias	Drug	Dose	Dose unit	Number of participants	Mean age (years)	Female participants (%)
NCT02469155	DSM-5	6	inpatient	Unclear	Placebo	0	mg/d	169	43.5	26
					Risperidone	4	mg/d	157	42.4	24
					Lumateperone	20	mg/d	166	42.7	27
					Lumateperone	60	mg/d	162	41.9	28
NCT04512066	DSM-5	4	inpatient	Unclear	Placebo	0	mg/d	54	33.2	26
					Risperidone	4	mg/d	56	32.9	25
Nakamura 2009	DSM-IV	6	inpatient	Low	Placebo	0	mg/d	90	41.9	22
					Lurasidone	80	mg/d	90	39.7	24
Nakamura 2016	DSM-IV-TR	12	NA	High	Cariprazine	3	mg/d	11	43.2	45
					Cariprazine	6	mg/d	16	43.1	56
					Cariprazine	9	mg/d	11	43.2	55
Nasrallah 2010	DSM-IV-TR	13	inpatient	Low	Placebo	0	mg/4w	125	41.1	38
					Paliperidone_LAI1M	25	mg/4w	129	40.8	35
					Paliperidone_LAI1M	50	mg/4w	128	39	28
					Paliperidone_LAI1M	100	mg/4w	131	42.3	35
Nasrallah 2013	DSM-IV	6	inpatient	Low	Placebo	0	mg/d	124	38.2	27
					Lurasidone	40	mg/d	121	40.7	33
					Lurasidone	80	mg/d	118	38.6	36
					Lurasidone	120	mg/d	123	37.7	26
Nasser 2016	DSM-IV-TR	8	inpatient	Low	Placebo	0	mg/4w	112	42.8	28
					Risperidone_RBP-7000	90	mg/4w	111	40.5	16

Study name	Diagnostic criteria	Duration (weeks)	In/outpatient	Risk of Bias	Drug	Dose	Dose unit	Number of participants	Mean age (years)	Female participants (%)
					Risperidone_RBP-7000	120	mg/4w	114	40.4	26
Ogasa 2012	DSM-IV	6	inpatient	Low	Placebo	0	mg/d	49	38.1	16
					Lurasidone	40	mg/d	49	39.8	28
					Lurasidone	120	mg/d	47	41	27
Oosthuizen 2004	DSM-IV	6	Mixed	Low	Haloperidol	2	mg/d	20	26.8	30
					Haloperidol	8	mg/d	20	28.9	50
Pandina 2010	DSM-IV	13	inpatient	Low	Placebo	0	mg/4w	160	39	34
					Paliperidone_LAI1M	25	mg/4w	155	39	28
					Paliperidone_LAI1M	100	mg/4w	161	39	33
					Paliperidone_LAI1M	150	mg/4w	160	39	36
Patil 2007	DSM-IV-TR	4	inpatient	Unclear	Placebo	0	mg/d	62	41	22
					Olanzapine	15	mg/d	34	42.3	26
Peuskens 1995	DSM-III-R	8	inpatient	Low	Risperidone	1	mg/d	226	38.4	28
					Risperidone	4	mg/d	227	38.1	33
					Risperidone	8	mg/d	228	37.6	37
					Haloperidol	10	mg/d	223	38.1	34
					Risperidone	12	mg/d	225	37.9	37
					Risperidone	16	mg/d	223	38.5	38
Potkin 2003	DSM-IV	4	inpatient	Unclear	Placebo	0	mg/d	103	38.8	29
					Risperidone	6	mg/d	95	38.6	28
					Aripiprazole	20	mg/d	98	38.1	28
					Aripiprazole	30	mg/d	96	40.2	35

Study name	Diagnostic criteria	Duration (weeks)	In/outpatient	Risk of Bias	Drug	Dose	Dose unit	Number of participants	Mean age (years)	Female participants (%)
Potkin 2007	DSM-IV	6	inpatient	Low	Placebo	0	mg/d	60	42	21
					Risperidone	6	mg/d	56	43	40
					Asenapine	10	mg/d	58	38	23
Potkin 2008a	DSM-IV	6	outpatient	Low	Placebo	0	mg/d	117	39.3	29
					Iloperidone	4	mg/d	113	38.4	32
					Iloperidone	8	mg/d	114	37	25
					Iloperidone	12	mg/d	115	40.1	27
					Haloperidol	15	mg/d	114	39.1	31
Potkin 2008b	DSM-IV	6	inpatient	Low	Placebo	0	mg/d	152	38.8	33
					Iloperidone	8	mg/d	143	38.4	31
					Risperidone	8	mg/d	146	37.5	25
					Iloperidone	16	mg/d	149	39.3	29
Potkin 2008c	DSM-IV	6	inpatient	Low	Placebo	0	mg/d	152	39	41
					Risperidone	8	mg/d	148	39.8	39
					Iloperidone	16	mg/d	230	38.9	40
					Iloperidone	24	mg/d	141	37.3	32
Potkin 2015	DSM-IV	6	inpatient	Low	Placebo	0	mg/d	71	41	24
					Haloperidol	10	mg/d	72	40	19
					Lurasidone	20	mg/d	71	40.7	28
					Lurasidone	40	mg/d	65	42	31
					Lurasidone	80	mg/d	70	42.2	27
Potkin 2020	DSM-5	4	inpatient	Low	Placebo	0	mg/d	112	41.1	42
					Olanzapine	20	mg/d	120	41.5	39
					Olanzapine-Samidorphan	20	mg/d	124	40.8	37

Study name	Diagnostic criteria	Duration (weeks)	In/outpatient	Risk of Bias	Drug	Dose	Dose unit	Number of participants	Mean age (years)	Female participants (%)
Puech 1998	DSM-III-R	4	inpatient	Low	Haloperidol	16	mg/d	61	36.6	44
					Amisulpride	100	mg/d	58	36.4	31
					Amisulpride	400	mg/d	62	35.6	33
					Amisulpride	800	mg/d	63	36.7	52
					Amisulpride	1,200.00	mg/d	65	34.8	31
Rifkin 1991	RDC and DSM-III	6	inpatient	Low	Haloperidol	10	mg/d	23	36.9	38
					Haloperidol	30	mg/d	19	32.6	38
					Haloperidol	80	mg/d	22	33.3	38
Saito 2020	DSM-IV-TR	6	Mixed	Low	Placebo	0	mg/d	47	15.6	57
					Blonanserin	8	mg/d	51	15.3	59
					Blonanserin	16	mg/d	52	15.6	56
Sarin 2004	ICD-10	6	outpatient	High	Aripiprazole	10	mg/d	65	35	34
					Aripiprazole	15	mg/d	61	33	39
Schmidt 2014	DSM-IV	6	inpatient	Unclear	Placebo	0	mg/d	55	38	40
					Olanzapine	15	mg/d	81	38.6	47
Shen 2014	DSM-IV-TR	6	inpatient	High	Placebo	0	mg/d	71	39.6	27
					Olanzapine	15	mg/d	71	40.1	35
Simpson 1967	Clinical Diagnosis	14	inpatient	Unclear	Placebo	0	mg/d	8	36.5	0
					Haloperidol	6	mg/d	8	36.5	0
					Haloperidol	30	mg/d	8	36.5	0
Simpson 1999	DSM-III-R	16	inpatient	Low	Clozapine	100	mg/d	14	44.8	57
					Clozapine	300	mg/d	17	44.8	59
					Clozapine	600	mg/d	17	44.8	59
Singh 2011	DSM-IV	6	inpatient	Low	Placebo	0	mg/d	51	15.7	55

Study name	Diagnostic criteria	Duration (weeks)	In/outpatient	Risk of Bias	Drug	Dose	Dose unit	Number of participants	Mean age (years)	Female participants (%)
					Paliperidone	2	mg/d	54	15.1	44
					Paliperidone	5	mg/d	48	15.3	35
					Paliperidone	10	mg/d	47	15.5	30
Study 104	DSM-III-R	4	NA	Unclear	Placebo	0	mg/d	47	39.6	12
					Ziprasidone	10	mg/d	44	39.2	13
					Ziprasidone	40	mg/d	55	41.1	5
					Ziprasidone	80	mg/d	47	39.7	19
Study 115 2000	DSM-III-R	6	inpatient	Unclear	Placebo	0	mg/d	80	38.9	35
					Haloperidol	15	mg/d	82	38.8	29
					Ziprasidone	40	mg/d	86	40.3	39
					Ziprasidone	120	mg/d	76	40	29
					Ziprasidone	200	mg/d	82	38.2	36
Study 93202 2002	DSM-III-R	4	inpatient	Unclear	Placebo	0	mg/d	35	37.9	17
					Haloperidol	20	mg/d	33	38.8	12
					Aripiprazole	30	mg/d	33	33	6
Study 94202 2002	DSM-IV	4	inpatient	Low	Placebo	0	mg/d	57	38	17
					Aripiprazole	2	mg/d	58	39.8	20
					Aripiprazole	10	mg/d	57	37.8	18
					Haloperidol	10	mg/d	61	38.9	17
					Aripiprazole	30	mg/d	60	38.8	25
Study RIS-USA-72 1996	DSM-IV	4	inpatient	Low	Placebo	0	mg/d	79	37	22
					Risperidone	4	mg/d	82	38	21
					Risperidone	8	mg/d	75	38.5	18

Study name	Diagnostic criteria	Duration (weeks)	In/outpatient	Risk of Bias	Drug	Dose	Dose unit	Number of participants	Mean age (years)	Female participants (%)
Takahashi 2013	DSM-IV-TR	13	NA	Low	Placebo	0	mg/4w	164	44	49
					Paliperidone_LAI1M	75	mg/4w	158	46	36
Walling 2019	DSM-IV-TR	4	inpatient	Low	Placebo	0	mg/d	63	41.2	24
					Risperidone	6	mg/d	26	41.3	31
Zborowski 1995	DSM-III-R/DSM-IV	8	inpatient	Low	Placebo	0	mg/d	106	38	22
					Haloperidol	16	mg/d	113	39	25
					Sertindole	20	mg/d	111	38	24
					Sertindole	24	mg/d	108	37	25
Zimbroff 1997	DSM-III-R/DSM-IV	8	inpatient	Low	Placebo	0	mg/d	71	38.7	22
					Haloperidol	4	mg/d	68	38.1	17
					Haloperidol	8	mg/d	63	39.9	19
					Sertindole	12	mg/d	72	37.8	20
					Haloperidol	16	mg/d	68	39	24
					Sertindole	20	mg/d	65	40.4	24
					Sertindole	24	mg/d	70	39.5	31
van Kammen 1996	DSM-III-R	5.7	inpatient	Low	Placebo	0	mg/d	38	37.1	4
					Sertindole	8	mg/d	35	39.7	6
					Sertindole	12	mg/d	40	38.6	5
					Sertindole	20	mg/d	40	36.8	2

N= number of participants analysed, ICD 9/10 = International Classification of Diseases, 9th/10th Revision, DSM-III, -III-R, -IV, -IV-TR, -V = different versions of the Diagnostic and Statistical Manual of Mental Disorders, n.i./NA = not indicated/not available, IR= immediate release, XR= extended release, LAI= long-acting injectable, Some reports provided data for several studies.

References

1. Arvanitis LA, Miller BG, group Sts. Multiple fixed doses of "Seroquel" (quetiapine) in patients with acute exacerbation of schizophrenia: a comparison with haloperidol and placebo. BiolPsychiatry. 1997;42:233-46.
2. Barbato LM, Newcomer JW, Heisterberg J. Efficacy and metabolic profile of bifeprunox in patients with schizophrenia. Schizophr Bull. 2007;33(2):419.
3. Beasley CM, Sanger T, Satterlee W. Olanzapine versus placebo: results of a double-blind fixed dose olanzapine trial. Psychopharmacology. 1996;124:159-67.
4. Beasley CM, Tollefson GD, Tran P, Satterlee W, Sanger T, Hamilton S, et al. Olanzapine versus haloperidol and placebo. Acute phase results of the american double-blind olanzapine trial. Neuropsychopharmacology. 1996;14:111-23.
5. Beasley CM, Hamilton SH, Crawford AM, Dellva MA, Tollefson GD, Tran PV, et al. Olanzapine versus haloperidol: acute phase results of the international double-blind olanzapine trial. EurNeuropsychopharmacol. 1997;7:125-37.
6. Berger GE, Proffitt TM, McConchie M, Kerr M, Markulev C, Yuen HP, et al. Dosing quetiapine in drug-naive first-episode psychosis: a controlled, double-blind, randomized, single-center study investigating efficacy, tolerability, and safety of 200 mg/day vs. 400 mg/day of quetiapine fumarate in 141 patients aged 15 to 25 years. J Clin Psychiatry. 2008; 69(11):1702-14
7. Bugarski-Kirola D, Wang A, Abi-Saab D, Blättler T. A phase II/III trial of bitopertin monotherapy compared with placebo in patients with an acute exacerbation of schizophrenia–results from the CandleLyte study. European Neuropsychopharmacology. 2014;24(7):1024-36.
8. Cantillon M. Efficacy and safety of novel dopamine serotonin stabilizer rp 5063 in acute schizophrenia and schizoaffective disorder. Schizophrenia research. 2014(153):S22.
9. Canuso CM, Lindenmayer JP, Kosik-Gonzalez C, Turkoz I, Carothers J, Bossie CA, et al. A randomized, double-blind, placebo-controlled study of 2 dose ranges of paliperidone extended-release in the treatment of subjects with schizoaffective disorder. J Clin Psychiatry. 2010; 71(5):587-98
10. Casey DE, Sands EE, Heisterberg J, Yang H-M. Efficacy and safety of bifeprunox in patients with an acute exacerbation of schizophrenia: results from a randomized, double-blind, placebo-controlled, multicenter, dose-finding study. Psychopharmacology. 2008;200(3):317-31.
11. Chouinard G, Jones B, Remington G. Canadian placebo-controlled study of fixed doses of risperidone and haloperidol in the treatment of chronic schizophrenic patients. JClinPsychopharmacol. 1993;13:25-40.
12. Citrome L, Walling DP, Zeni CM, Starling BR, Terahara T, Kuriki M, et al. Efficacy and Safety of HP-3070, an Asenapine Transdermal System, in Patients With Schizophrenia: A Phase 3, Randomized, Placebo-Controlled Study. J Clin Psychiatry. 2020;82(1):20m13602.
13. Cooper S, Tweed J, Raniwalla J, Butler A, Welch C. A placebo-controlled comparison of zotepine versus chlorpromazine in patients with acute exacerbation of schizophrenia. Acta Psychiatrica Scandinavica. 2000;101(3):218-25.
14. Coppola D, Melkote R, Lannie C, Singh J, Nuamah I, Gopal S, et al. Efficacy and Safety of Paliperidone Extended Release 1.5 mg/day-A Double-blind, Placebo- and Active-Controlled, Study in the Treatment of Patients with Schizophrenia. Psychopharmacol Bull. 2011;44(2):54-72.
15. Correll CU, Skuban A, Ouyang J, Hobart M, Pfister S, McQuade RD, et al. Efficacy and Safety of Brexpiprazole for the Treatment of Acute Schizophrenia: A 6-Week

Randomized, Double-Blind, Placebo-Controlled Trial. Am J Psychiatry. 2015;172(9):870-80.
16. Correll CU, Davis RE, Weingart M, Saillard J, O'Gorman C, Kane JM, et al. Efficacy and Safety of Lumateperone for Treatment of Schizophrenia: A Randomized Clinical Trial. JAMA Psychiatry. 2020.
17. Correll CU, Litman RE, Filts Y, Llaudó J, Naber D, Torres F, et al. Efficacy and safety of once-monthly Risperidone ISM(®) in schizophrenic patients with an acute exacerbation. NPJ Schizophr. 2020;6(1):37.
18. Corrigan MH, Gallen CC, Bonura ML, Merchant KM; Sonepiprazole Study Group. Effectiveness of the selective D4 antagonist sonepiprazole in schizophrenia: a placebo-controlled trial. Biol Psychiatry. 2004;55(5):445-51.
19. Cutler AJ, Marcus RN, Hardy SA, O'Donnell A, Carson WH, McQuade RD. The efficacy and safety of lower doses of aripiprazole for the treatment of patients with acute exacerbation of schizophrenia. CNS Spectr. 2006;11(9):691-702.
20. Cutler AJ, Kalali AH, Weiden PJ, Hamilton J, Wolfgang CD. Four-week, double-blind, placebo-and ziprasidone-controlled trial of iloperidone in patients with acute exacerbations of schizophrenia. Journal of clinical psychopharmacology. 2008;28(2):S20-S8.
21. Cutler AJ, Tran-Johnson T, Kalali A, Astrom M, Brecher M, Meulien D. A failed 6-week,randomized, double-blind, placebo-controlled study of once-daily extended release quetiapine fumarate in patients with acute schizophrenia: lessons learned. Psychopharmacology bulletin. 2010;43(4):37-69.
22. Daniel DG, Zimbroff DL, Potkin SG, Reeves KR, Harrigan EP, Lakshminarayanan M. Ziprasidone 80 mg/day and 160 mg/day in the acute exacerbation of schizophrenia and schizoaffective disorder: A 6-week placebo-controlled trial. Neuropsychopharmacology. 1999;20(5):491-505.
23. Davidson M, Emsley R, Kramer M, Ford L, Pan G, Lim P, et al. Efficacy, safety and early response of paliperidone extended-release tablets (paliperidone ER): results of a 6-week, randomized, placebo-controlled study. Schizophrenia research. 2007;93(1-3):117-30.
24. Downing AM, Kinon BJ, Millen BA, Zhang L, Liu L, Morozova MA, et al. A double-blind, placebo-controlled comparator study of LY2140023 monohydrate in patients with schizophrenia. BMC psychiatry. 2014;14(1):351.
25. Durgam S, Starace A, Li D, Migliore R, Ruth A, Nemeth G, et al. An evaluation of the safety and efficacy of cariprazine in patients with acute exacerbation of schizophrenia: a phase II, randomized clinical trial. Schizophrenia research. 2014;152(2-3):450-7.
26. Durgam S, Cutler AJ, Lu K, Migliore R, Ruth A, Laszlovszky I, et al. Cariprazine in acute exacerbation of schizophrenia: a fixed-dose, phase 3, randomized, double-blind, placebo- and active-controlled trial. J Clin Psychiatry. 2015;76(12):e1574-82.
27. Durgam S, Litman RE, Papadakis K, Li D, Németh G, Laszlovszky I. Cariprazine in the treatment of schizophrenia: a proof-of-concept trial. Int Clin Psychopharmacol. 2016;31(2):61-8.
28. Egan MF, Zhao X, Smith A, Troyer MD, Uebele VN, Pidkorytov V, et al. Randomized controlled study of the T-type calcium channel antagonist MK-8998 for the treatment of acute psychosis in patients with schizophrenia. Hum Psychopharmacol. 2013;28(2):124-33.
29. Fabre LF Jr, Arvanitis L, Pultz J, Jones VM, Malick JB, Slotnick VB. ICI 204,636, a novel, atypical antipsychotic: early indication of safety and efficacy in patients with chronic and subchronic schizophrenia. Clin Ther. 1995;17(3):366-78.

30. Findling RL, Robb A, Nyilas M, Forbes RA, Jin N, Ivanova S, et al. A multiple-center, randomized, double-blind, placebo-controlled study of oral aripiprazole for treatment of adolescents with schizophrenia. Am J Psychiatry. 2008;165(11):1432-41.
31. Findling RL, McKenna K, Earley WR, Stankowski J, Pathak S. Efficacy and safety of quetiapine in adolescents with schizophrenia investigated in a 6-week, double-blind, placebo-controlled trial. J Child Adolesc Psychopharmacol. 2012;22(5):327-42.
32. Findling RL, Landbloom RP, Mackle M, Pallozzi W, Braat S, Hundt C, et al. Safety and Efficacy from an 8 Week Double-Blind Trial and a 26 Week Open-Label Extension of Asenapine in Adolescents with Schizophrenia. J Child Adolesc Psychopharmacol. 2015;25(5):384-96.
33. Garcia E, Robert M, Peris F, Nakamura H, Sato N, Terazawa Y. The efficacy and safety of blonanserin compared with haloperidol in acute-phase schizophrenia. CNS drugs. 2009;23(7):615-25.
34. Goff DC, Posever T, Herz L, Simmons J, Kletti N, Lapierre K, et al. An exploratory haloperidol-controlled dose-finding study of ziprasidone in hospitalized patients with schizophrenia or schizoaffective disorder. J Clin Psychopharmacol. 1998;18(4):296-304.
35. Goff DC, McEvoy JP, Citrome L, Mech AW, Bustillo JR, Gil R, et al. High-dose oral ziprasidone versus conventional dosing in schizophrenia patients with residual symptoms: the ZEBRAS study. J Clin Psychopharmacol. 2013;33(4):485-90.
36. Goldman R, Loebel A, Cucchiaro J, Deng L, Findling RL. Efficacy and Safety of Lurasidone in Adolescents with Schizophrenia: A 6-Week, Randomized Placebo-Controlled Study. J Child Adolesc Psychopharmacol. 2017;27(6):516-525.
37. Gopal S, Hough DW, Xu H, Lull JM, Gassmann-Mayer C, Remmerie BM, et al. Efficacy and safety of paliperidone palmitate in adult patients with acutely symptomatic schizophrenia: a randomized, double-blind, placebo-controlled, dose-response study. Int Clin Psychopharmacol. 2010;25(5):247-56.
38. Haas M, Unis AS, Armenteros J, Copenhaver MD, Quiroz JA, Kushner SF. A 6-week, randomized, double-blind, placebo-controlled study of the efficacy and safety of risperidone in adolescents with schizophrenia. J Child Adolesc Psychopharmacol. 2009;19(6):611-21.
39. Haas M, Eerdekens M, Kushner S, Singer J, Augustyns I, Quiroz J, et al. Efficacy, safety and tolerability of two dosing regimens in adolescent schizophrenia: double-blind study. Br J Psychiatry. 2009;194(2):158-64.
40. Hale A, Azorin JM, Kasper S, Maier W, Syvalahti E, van der Burght M, et al. Sertindole is associated with a low level of extrapyramidal symptoms in schizophrenic patients: Results of a phase III trial. Int J Psychiatry Clin Pract. 2000;4(1):47-54.
41. Heinrich K, Klieser E, Lehmann E, Kinzler E, Hruschka H. Risperidone versus clozapine in the treatment of schizophrenic patients with acute symptoms: a double blind, randomized trial. Prog Neuropsychopharmacol Biol Psychiatry. 1994;18(1):129-37.
42. Hera 041-021. NCT00156117. Efficacy and Safety of Asenapine With Placebo and Olanzapine (41021)(P05933). https://clinicaltrials.gov/study/NCT00156117
43. Higuchi T, Ishigooka J, Iyo M, Yeh CB, Ebenezer EG, Liang KY, et al. Lurasidone in the treatment of schizophrenia: Results of a double-blind, placebo-controlled trial in Asian patients. Asia Pac Psychiatry. 2019;11(2):e12352.
44. Higuchi T, Iyo M, Kwon JS, Chou YH, Chen HK, Chen JY, et al. Randomized, double-blind, placebo, and risperidone-controlled study of lurasidone in the

treatment of schizophrenia: Results of an inconclusive 6-week trial. Asia Pac Psychiatry. 2019;11(3):e12354.
45. Hirayasu Y, Tomioka M, Iizumi Misuzu, Kikuchi H. A double-blind, placebo-controlled, comparative study of paliperidone Extended-Release (ER) tablets in patients with schizophrenia. Jpn J Clin Psychopharmacol. 2010;13:2077-2103.
46. Honer WG, MacEwan GW, Gendron A, Stip E, Labelle A, Williams R, et al. A randomized, double-blind, placebo-controlled study of the safety and tolerability of high-dose quetiapine in patients with persistent symptoms of schizophrenia or schizoaffective disorder. The Journal of clinical psychiatry. 2012;73(1):13-20.
47. ILPB202 in US FDA "Iloperidone medical review" (2009). FDA Drug Approval Documents. Paper 6. http://digitalcommons.ohsu.edu/fdadrug/6
48. Ishigooka J, Iwashita S, Tadori Y. Efficacy and safety of brexpiprazole for the treatment of acute schizophrenia in Japan: A 6-week, randomized, double-blind, placebo-controlled study. Psychiatry Clin Neurosci. 2018;72(9):692-700.
49. Iwata N, Ishigooka J, Kim WH, Yoon BH, Lin SK, Sulaiman AH, et al. Efficacy and safety of blonanserin transdermal patch in patients with schizophrenia: A 6-week randomized, double-blind, placebo-controlled, multicenter study. Schizophr Res. 2020;215:408-415.
50. Iyo M, Ishigooka J, Nakamura M, Sakaguchi R, Okamoto K, Mao Y, et al. Efficacy and safety of lurasidone in acutely psychotic patients with schizophrenia: A 6-week, randomized, double-blind, placebo-controlled study. Psychiatry Clin Neurosci. 2021;75(7):227–35.
51. Kahn RS, Schulz SC, Palazov VD, Reyes EB, Brecher M, Svensson O, et al. Efficacy and tolerability of once-daily extended release quetiapine fumarate in acute schizophrenia: a randomized, double-blind, placebo-controlled study. JClinPsychiatry. 2007;68(6):832-42.
52. Kane JM, Carson WH, Saha AR, McQuade RD, Ingenito GG, Zimbroff DL, et al. Efficacy and safety of aripiprazole and haloperidol versus placebo in patients with schizophrenia and schizoaffective disorder. JClinPsychiatry. 2002;63(9):763-71.
53. Kane JM, Eerdekens M, Lindenmayer JP, Keith SJ, Lesem M, Karcher K. Long-acting injectable risperidone: efficacy and safety of the first long-acting atypical antipsychotic. Am J Psychiatry. 2003;160(6):1125-32.
54. Kane JM, Canas F, Kramer M, Ford L, Gassmann-Mayer C, Lim P, et al. Treatment of schizophrenia with paliperidone extended-release tablets: a 6-week placebo-controlled trial. Schizophr Res. 2007 Feb;90(1-3):147-61. 21.
55. Kane JM, Cohen M, Zhao J, Alphs L, Panagides J. Efficacy and safety of asenapine in a placebo- and haloperidol-controlled trial in patients with acute exacerbation of schizophrenia. JClinPsychopharmacol. 2010;30(2):106-15.
56. Kane JM, Peters-Strickland T, Baker RA, Hertel P, Eramo A, Jin N, et al. Aripiprazole once-monthly in the acute treatment of schizophrenia: findings from a 12-week, randomized, double-blind, placebo-controlled study. The Journal of clinical psychiatry. 2014;75(11):1254-60.
57. Kane JM, Skuban A, Ouyang J, Hobart M, Pfister S, McQuade RD, et al. A multicenter, randomized, double-blind, controlled phase 3 trial of fixed-dose brexpiprazole for the treatment of adults with acute schizophrenia. Schizophrenia research. 2015;164(1-3):127-35.
58. Kane JM, Skuban A, Ouyang J, Hobart M, Pfister S, McQuade RD, et al. A multicenter, randomized, double-blind, controlled phase 3 trial of fixed-dose brexpiprazole for the treatment of adults with acute schizophrenia. Schizophrenia research. 2015;164(1-3):127-35.

59. Kane JM, Zukin S, Wang Y, Lu K, Ruth A, Nagy K, et al. Efficacy and Safety of Cariprazine in Acute Exacerbation of Schizophrenia: Results From an International, Phase III Clinical Trial. J Clin Psychopharmacol. 2015;35(4):367-73.
60. Keck P Jr, Buffenstein A, Ferguson J, Feighner J, Jaffe W, Harrigan EP, et al. Ziprasidone 40 and 120 mg/day in the acute exacerbation of schizophrenia and schizoaffective disorder: a 4-week placebo-controlled trial. Psychopharmacology. 1998;140(2):173-84.
61. King DJ, Link CG, Kowalcyk B. A comparison of bd and tid dose regimens of quetiapine (seroquel) in the treatment of schizophrenia. Psychopharmacology. 1998. p.139-46.
62. Kinon BJ, Volavka J, Stauffer V, Edwards SE, Liu-Seifert H, Chen L, et al. Standard and higher dose of olanzapine in patients with schizophrenia or schizoaffective disorder: a randomized, double-blind, fixed-dose study. Journal of clinical psychopharmacology. 2008;28(4):392-400.
63. Kinon BJ, Zhang L, Millen BA, Osuntokun OO, Williams JE, Kollack-Walker S, et al. A multicenter, inpatient, phase 2, double-blind, placebo-controlled dose-ranging study of LY2140023 monohydrate in patients with DSM-IV schizophrenia. Journal of clinical psychopharmacology. 2011;31(3):349-55.
64. Kinoshita T, Bai YM, Kim JH, Miyake M, Oshima N. Efficacy and safety of asenapine in Asian patients with an acute exacerbation of schizophrenia: a multicentre, randomized, double-blind, 6-week, placebo-controlled study. Psychopharmacology (Berl). 2016 Jul;233(14):2663-74.
65. Klieser E, Lehmann E. Experimental examination of trazodone. Clin Neuropharmacol. 1989;12 Suppl 1:S18-24.
66. Kramer M, Litman R, Hough D, Lane R, Lim P, Liu Y, et al. Paliperidone palmitate, a potential long-acting treatment for patients with schizophrenia. Results of a randomized, double-blind, placebo-controlled efficacy and safety study. The international journal of neuropsychopharmacology. 2010;13(5):635-47.
67. Landbloom R, Mackle M, Wu X, Kelly L, Snow-Adami L, McIntyre RS, et al. Asenapine for the treatment of adults with an acute exacerbation of schizophrenia: results from a randomized, double-blind, fixed-dose, placebo-controlled trial with olanzapine as an active control. CNS spectrums. 2017;22(4):333-41.
68. Lane HY, Chang WH, Chiu CC, Huang MC, Lee SH, Chen JY. A pilot double-blind, dose-comparison study of risperidone in drug-naive, first-episode schizophrenia. J Clin Psychiatry. 2001;62(12):994-995. doi:10.4088/jcp.v62n1214c
69. Lauriello J, Lambert T, Andersen S, Lin D, Taylor CC, McDonnell D. An 8-week, double-blind, randomized, placebo-controlled study of olanzapine long-acting injection in acutely ill patients with schizophrenia. The Journal of clinical psychiatry. 2008;69(5):790-9.
70. Lieberman JA, Davis RE, Correll CU, Goff DC, Kane JM, Tamminga CA, et al. ITI-007 for the treatment of schizophrenia: a 4-week randomized, double-blind, controlled trial. Biological psychiatry. 2016;79(12):952-61.
71. Lindenmayer JP, Brown D, Liu S, Brecher M, Meulien D. The efficacy and tolerability of once-daily extended release quetiapine fumarate in hospitalized patients with acute schizophrenia: a 6-week randomized, double-blind, placebo-controlled study. Psychopharmacology bulletin. 2008;41(3):11-35.
72. Lindenmayer JP, Citrome L, Khan A, Kaushik S, Kaushik S. A randomized, double-blind, parallel-group, fixed-dose, clinical trial of quetiapine at 600 versus 1200 mg/d for patients with treatment-resistant schizophrenia or schizoaffective disorder. Journal of clinical psychopharmacology. 2011;31(2):160-8.

73. Litman RE, Smith MA, Doherty JJ, Cross A, Raines S, Gertsik L, et al. AZD8529, a positive allosteric modulator at the mGluR2 receptor, does not improve symptoms in schizophrenia: A proof of principle study. Schizophrenia research. 2016;172(1-3):152-7.
74. Litman RE, Smith MA, Desai DG, Simpson T, Sweitzer D, Kanes SJ. The selective neurokinin 3 antagonist AZD2624 does not improve symptoms or cognition in schizophrenia: a proof-of-principle study. Journal of clinical psychopharmacology. 2014;34(2):199-204.
75. Loebel A, Cucchiaro J, Sarma K, Xu L, Hsu C, Kalali AH, et al. Efficacy and safety of lurasidone 80 mg/day and 160 mg/day in the treatment of schizophrenia: a randomized, double-blind, placebo- and active-controlled trial. Schizophr Res. 2013;145(1-3):101-9.
76. Loebel A, Silva R, Goldman R, Watabe K, Cucchiaro J, Citrome L, et al. Lurasidone Dose Escalation in Early Nonresponding Patients With Schizophrenia: A Randomized, Placebo-Controlled Study. The Journal of clinical psychiatry. 2016.
77. Louza Neto MR, Müller-Spahn F, Rüther E, Scherer J. Haloperidol plasma level after a test dose as predictor for the clinical response to treatment in acute schizophrenic patients. Pharmacopsychiatry. 1988;21(5):226-31. 47.
78. Marder SR, Meibach RC. Risperidone in the treatment of schizophrenia. AmJPsychiatry. 1994;151:825-35.
79. Marder SR, Kramer M, Ford L, Eerdekens E, Lim P, Eerdekens M, et al. Efficacy and safety of paliperidone extended-release tablets: results of a 6-week, randomized, placebo-controlled study. Biological psychiatry. 2007;62(12):1363-70.
80. Matsumoto H, Ishigooka J, Ono H, Tadori Y. Safety and efficacy from a 6-week double-blind study and a 52-week open-label extension of aripiprazole in adolescents with schizophrenia in Japan. Psychiatry Clin Neurosci. 2018;72(9):701-712.
81. McEvoy JP, Hogarty GE, Steingard S. Optimal dose of neuroleptic in acute schizophrenia. A controlled study of the neuroleptic threshold and higher haloperidol dose. Arch Gen Psychiatry. 1991;48(8):739-745. doi:10.1001/archpsyc.1991.01810320063009
82. McEvoy JP, Daniel DG, Carson WH, Jr., McQuade RD, Marcus RN. A randomized, double-blind, placebo-controlled, study of the efficacy and safety of aripiprazole 10, 15 or 20 mg/day for the treatment of patients with acute exacerbations of schizophrenia. JPsychiatrRes. 2007;41(11):895-905.
83. Meltzer HY, Arvanitis L, Bauer D, Rein W, Group M-TS. Placebo-controlled evaluation of four novel compounds for the treatment of schizophrenia and schizoaffective disorder. American Journal of Psychiatry. 2004;161(6):975-84.
84. Meltzer H, Barbato L, Heisterberg J, Yeung P, Shapira N. A randomized, double-blind, placebo-controlled efficacy and safety study of bifeprunox as treatment for patients with acutely exacerbated schizophrenia. Schizophrenia bulletin. 2007.
85. Meltzer HY, Cucchiaro J, Silva R, Ogasa M, Phillips D, Xu J, et al. Lurasidone in the treatment of schizophrenia: a randomized, double-blind, placebo- and olanzapine-controlled study. Am J Psychiatry. 2011;168(9):957-67.
86. Meltzer HY, Elkis H, Vanover K, Weiner DM, van Kammen DP, Peters P, et al. Pimavanserin, a selective serotonin (5-HT) 2A-inverse agonist, enhances the efficacy and safety of risperidone, 2 mg/day, but does not enhance efficacy of haloperidol, 2 mg/day: comparison with reference dose risperidone, 6 mg/day. Schizophrenia research. 2012;141(2-3):144-52.
87. Meltzer H, Lindenmayer J-P, Kwentus J, Share D, Johnson R, Jayathilake K. A six month randomized controlled trial of long acting injectable risperidone 50 and 100

mg in treatment resistant schizophrenia. Schizophrenia research. 2014;154(1-3):14-22.
88. Meltzer HY, Risinger R, Nasrallah HA, Du Y, Zummo J, Corey L, et al. A randomized, double-blind, placebo-controlled trial of aripiprazole lauroxil in acute exacerbation of schizophrenia. The Journal of clinical psychiatry. 2015;76(8):1085-90.
89. Meltzer HY, Share DB, Jayathilake K, Salomon RM, Lee MA. Lurasidone Improves Psychopathology and Cognition in Treatment-Resistant Schizophrenia. Journal of clinical psychopharmacology. 2020;40(3):240.
90. Merlo MC, Hofer H, Gekle W, Berger G, Ventura J, Panhuber I, et al. Risperidone, 2 mg/day vs. 4 mg/day, in first-episode, acutely psychotic patients: treatment efficacy and effects on fine motor functioning. J Clin Psychiatry. 2002;63(10):885-91.
91. NCT00563706. Study Evaluating Vabicaserin in Subjects With Schizophrenia. https://ClinicalTrials.gov/show/NCT00563706.
92. NCT00905307. Study to Evaluate the Efficacy, Safety, and Tolerability of Oral OPC-34712 and Aripiprazole for Treatment of Acute Schizophrenia (STEP 203). https://clinicaltrials.gov/study/NCT00905307
93. NCT01625000. Safety and Efficacy of MP-214 in Patients With Schizophrenia. https://clinicaltrials.gov/study/NCT01625000
94. NCT02469155. A Trial to Assess the Antipsychotic Efficacy of ITI-007 Over 6 Weeks of Treatment. https://clinicaltrials.gov/study/NCT02469155
95. NCT04512066. A Trial of the Efficacy and the Safety of RO6889450 (Ralmitaront) vs Placebo in Patients With an Acute Exacerbation of Schizophrenia or Schizoaffective Disorder. https://clinicaltrials.gov/study/NCT04512066
96. Nakamura M, Ogasa M, Guarino J, Phillips D, Severs J, Cucchiaro J, et al. Lurasidone in the treatment of acute schizophrenia: a double-blind, placebo-controlled trial. The Journal of clinical psychiatry. 2009;70(6):829-36.
97. Nakamura T, Kubota T, Iwakaji A, Imada M, Kapás M, Morio Y. Clinical pharmacology study of cariprazine (MP-214) in patients with schizophrenia (12-week treatment). Drug Des Devel Ther. 2016;10:327-38.
98. Nasrallah HA, Gopal S, Gassmann-Mayer C, Quiroz JA, Lim P, Eerdekens M, et al. A controlled, evidence-based trial of paliperidone palmitate, a long-acting injectable antipsychotic, in schizophrenia. Neuropsychopharmacology. 2010;35(10):2072-82.
99. Nasrallah HA, Silva R, Phillips D, Cucchiaro J, Hsu J, Xu J, et al. Lurasidone for the treatment of acutely psychotic patients with schizophrenia: a 6-week, randomized, placebo-controlled study. J Psychiatr Res. 2013;47(5):670-7.
100. Nasser AF, Henderson DC, Fava M, Fudala PJ, Twumasi-Ankrah P, Kouassi A, et al. Efficacy, Safety, and Tolerability of RBP-7000 Once-Monthly Risperidone for the Treatment of Acute Schizophrenia: An 8-Week, Randomized, Double-Blind, Placebo-Controlled, Multicenter Phase 3 Study. J Clin Psychopharmacol. 2016;36(2):130-40.
101. Ogasa M, Kimura T, Nakamura M, Guarino J. Lurasidone in the treatment of schizophrenia: a 6-week, placebo-controlled study. Psychopharmacology. 2013;225(3):519-30.
102. Oosthuizen P, Emsley R, Jadri Turner H, Keyter N. A randomized, controlled comparison of the efficacy and tolerability of low and high doses of haloperidol in the treatment of first-episode psychosis. Int J Neuropsychopharmacol. 2004;7(2):125-31.

103. Pandina GJ, Lindenmayer JP, Lull J, Lim P, Gopal S, Herben V, et al. A randomized, placebo-controlled study to assess the efficacy and safety of 3 doses of paliperidone palmitate in adults with acutely exacerbated schizophrenia. Journal of clinical psychopharmacology. 2010;30(3):235-44.
104. Patil ST, Zhang L, Martenyi F, Lowe SL, Jackson KA, Andreev BV, et al. Activation of mGlu2/3 receptors as a new approach to treat schizophrenia: a randomized Phase 2 clinical trial. Nature medicine. 2007;13(9):1102-7.
105. Peuskens J, Group. RS. Risperidone in the treatment of patients with chronic schizophrenia: a multi-national, multi-centre, double-blind, parallel-group study versus haloperidol. BrJPsychiatry. 1995;166(6):712-26.
106. Potkin SG, Saha AR, Kujawa MJ, Carson WH, Ali M, Stock E, et al. Aripiprazole, an antipsychotic with a novel mechanism of action, and risperidone vs placebo in patients with schizophrenia and schizoaffective disorder. ArchGenPsychiatry. 2003;60(7):681-90.
107. Potkin SG, Cohen M, Panagides J. Efficacy and tolerability of asenapine in acute schizophrenia: a placebo-and risperidone-controlled trial. The Journal of clinical psychiatry. 2007;68(10):1492-500.
108. Simpson GM, Angus JW, Edwards JG. A controlled study of haloperidol in chronic schizophrenia. Curr Ther Res Clin Exp. 1967;9(8):407-412.
109. Study 1 in Potkin SG, Litman RE, Torres R, Wolfgang CD. Efficacy of iloperidone in the treatment of schizophrenia: initial phase 3 studies. J Clin Psychopharmacol. 2008 Apr;28(2 Suppl 1):S4-11. 49.
110. Study 2 in Potkin SG, Litman RE, Torres R, Wolfgang CD. Efficacy of iloperidone in the treatment of schizophrenia: initial phase 3 studies. J Clin Psychopharmacol. 2008 Apr;28(2 Suppl 1):S4-11. 49.
111. Study 3 in Potkin SG, Litman RE, Torres R, Wolfgang CD. Efficacy of iloperidone in the treatment of schizophrenia: initial phase 3 studies. J Clin Psychopharmacol. 2008 Apr;28(2 Suppl 1):S4-11. 49.
112. Potkin SG, Kimura T, Guarino J. A 6-week, double-blind, placebo- and haloperidol-controlled, phase II study of lurasidone in patients with acute schizophrenia. Ther Adv Psychopharmacol. 2015 Dec;5(6):322-31. doi: 10.1177/2045125315606027. Erratum in: Ther Adv Psychopharmacol. 2015 Dec;5(6):369.
113. Potkin SG, Kunovac J, Silverman BL, Simmons A, Jiang Y, DiPetrillo L, McDonnell D. Efficacy and Safety of a Combination of Olanzapine and Samidorphan in Adult Patients With an Acute Exacerbation of Schizophrenia: Outcomes From the Randomized, Phase 3 ENLIGHTEN-1 Study. J Clin Psychiatry. 2020;81(2):19m12769.
114. Puech A, Fleurot O, Rein W. Amisulpride, an atypical antipsychotic, in the treatment of acute episodes of schizophrenia: a dose-ranging study vs. haloperidol. Acta PsychiatrScand. 1998;98(1):65-72.
115. Rifkin A, Doddi S, Karajgi B, Borenstein M, Wachspress M. Dosage of haloperidol for schizophrenia. Arch Gen Psychiatry. 1991;48(2):166-70.
116. Saito T, Sugimoto S, Sakaguchi R, Nakamura H, Ishigooka J. Efficacy and Safety of Blonanserin Oral Tablet in Adolescents with Schizophrenia: A 6-Week, Randomized Placebo-Controlled Study. J Child Adolesc Psychopharmacol. 2022;32(1):12-23.
117. Sarin A, Nagpal J, Bohra NK, Jiloha RC, Rao GP, Sharma SK, et al. Open labeled, randomized, switch-over study of two fixed doses (10/15mg) of aripiprazole : to evaluate its safety and efficacy in the treatment of Indian patients of schizophrenia. Indian J Psychiatry. 2004;46(1):64-71.

118. Schmidt ME, Kent JM, Daly E, Janssens L, Van Osselaer N, Hüsken G, et al. A double-blind, randomized, placebo-controlled study with JNJ-37822681, a novel, highly selective, fast dissociating D2 receptor antagonist in the treatment of acute exacerbation of schizophrenia. European Neuropsychopharmacology. 2012;22(10):721-33.
119. Shen JH, Zhao Y, Rosenzweig-Lipson S, Popp D, Williams JB, Giller E, et al. A 6-week randomized, double-blind, placebo-controlled, comparator referenced trial of vabicaserin in acute schizophrenia. Journal of psychiatric research. 2014;53:14-22.
120. Simpson GM, Josiassen RC, Stanilla JK, De Leon J, Nair C, Abraham G, et al. Double-blind study of clozapine dose response in chronic schizophrenia. AmJPsychiatry. 1999;156(11):1744-50.
121. Singh J, Robb A, Vijapurkar U, Nuamah I, Hough D. A randomized, double-blind study of paliperidone extended-release in treatment of acute schizophrenia in adolescents. Biol Psychiatry. 2011;70(12):1179-87.
122. Study 104. In US FDA Ziprasidone Clinical Review.
123. Study 115 2000. In US FDA Ziprasidone Clinical Review.
124. Study 93202 2002. In US FDA Aripiprazole Clinical Review.
125. Study 94202 2002. In US FDA Aripiprazole Clinical Review.
126. Study RIS-USA-72 1996. In US FDA Risperidone Clinical Review.
127. Takahashi N, Takahashi M, Saito T, Iizumi M, Saito Y, Shimizu H, et al. Randomized, placebo-controlled, double-blind study assessing the efficacy and safety of paliperidone palmitate in Asian patients with schizophrenia. Neuropsychiatric disease and treatment. 2013;9:1889.
128. Van Kammen DP, McEvoy JP, Targum SD, Kardatzke D, Sebree TB. A randomized, controlled, dose-ranging trial of sertindole in patients with schizophrenia. Psychopharmacology. 1996;124(1-2):168-75.
129. Walling DP, Banerjee A, Dawra V, Boyer S, Schmidt CJ, DeMartinis N. Phosphodiesterase 10A Inhibitor Monotherapy Is Not an Effective Treatment of Acute Schizophrenia. J Clin Psychopharmacol. 2019;39(6):575-582.
130. Zborowski J, Schmitz P, Staser J, O'Neil J, Giles K, Wallin B, et al. Efficacy and safety of sertindole in a trial of schizophrenic patients. BiolPsychiatry. 1995;37:661-2.
131. Zimbroff DL, Kane JM, Tamminga CA, Daniel DG, Mack RJ, Wozniak PJ, et al. Controlled, dose response study of sertindole and haloperidol in the treatment of schizophrenia. AmJPsychiatry. 1997;154(6):782-91.

eAppendix 7: Risk of bias assessment

Study	D1	D2	D3	D4	D5	D6	D7	Overall
Arvanitis 1997	Low	Unclear	Low	Low	Low	Low	Low	Low
Barbato 2007a	Unclear	Unclear	Unclear	Unclear	Unclear	Unclear	Unclear	Unclear
Barbato 2007b	Unclear	Unclear	Unclear	Unclear	Unclear	Unclear	Unclear	Unclear
Beasley 1996a	Low	Low	Low	Low	Low	Low	Low	Low
Beasley 1996b	Low	Low	Low	Low	Low	Low	Low	Low
Beasley 1997	Low	Low	Low	Low	Low	Low	Low	Low
Berger 2008	Low	Low	Low	Unclear	Low	Low	Low	Low
Bugarski-Kirola 2014	Unclear	Unclear	Unclear	Unclear	Low	Low	Low	Unclear
Cantillon 2014	Unclear	Unclear	Unclear	Unclear	Unclear	Unclear	Low	Unclear
Canuso 2010	Low	Low	Low	Low	Low	Low	Low	Low
Casey 2008	Unclear	Unclear	Unclear	Unclear	Low	High	Low	Unclear
Chouinard 1993	Low	Low	Low	Low	Low	Low	Low	Low
Citrome 2021	Low	Low	Low	Low	Low	High	Low	Unclear
Cooper 2000	Low	Low	Low	Low	Low	Low	Low	Low
Coppola 2011	Low	Low	Low	Low	Low	Low	Low	Low
Correll 2015	Low	Unclear	Unclear	Unclear	Low	Low	Low	Unclear
Correll 2020a	Low	Low	Low	Low	Low	Low	Low	Low
Correll 2020b	Low	Low	Low	Low	Low	Low	Low	Low
Corrigan 2004	Low	Low	Low	Low	Low	High	Low	Unclear
Cutler 2006	Unclear	Unclear	Low	Low	Low	High	Low	Unclear
Cutler 2008a	Low	Low	Low	Low	High	Low	Low	Unclear
Cutler 2008b	Unclear	Unclear	Low	Low	Low	Low	Low	Low
Daniel 1999	Low	Unclear	Unclear	Unclear	Low	Low	Low	Unclear
Davidson 2007	Low	Low	Low	Low	Low	Low	Low	Low
Downing 2014	Low	Low	Low	Low	Low	Low	Low	Low
Durgam 2014	Unclear	Unclear	Unclear	Unclear	Low	Low	Low	Unclear
Durgam 2015	Unclear	Unclear	Unclear	Unclear	Low	Low	Low	Unclear

Study	D1	D2	D3	D4	D5	D6	D7	Overall
Durgam 2016	Unclear	Unclear	Unclear	Unclear	Low	Unclear	Low	Unclear
Egan 2013	Low	Low	Low	Low	Low	Low	Low	Low
Fabre 1995	Unclear	Unclear	Unclear	Unclear	Low	Low	Low	Unclear
Findling 2008	Low	Low	Low	Low	Low	High	Low	Unclear
Findling 2012	Low	Unclear	Low	Low	Low	Low	Low	Low
Findling 2015	Low	Low	Low	Low	Low	Low	Low	Low
Garcia 2009	Low	Unclear	Low	Low	Low	High	Low	Unclear
Goff 1998	Unclear	Unclear	Unclear	Unclear	Low	Low	Low	Unclear
Goff 2013	Unclear	Unclear	Low	Low	Unclear	Low	Low	Unclear
Goldman 2017	Low	Low	Low	Low	Low	Low	Low	Low
Gopal 2010	Low	Low	Low	Low	Low	Low	Low	Low
Haas 2009a	Low	Unclear	Low	Low	Low	Low	Low	Low
Haas 2009b	Low	Unclear	Unclear	Unclear	Low	Low	Low	Unclear
Hale 2000	Low	Low	Low	Low	Low	Low	Low	Low
Heinrich 1994	Unclear	Unclear	Unclear	Unclear	Low	Low	Low	Unclear
Hera 041-021	Unclear	Unclear	Low	Unclear	High	High	Unclear	High
Higuchi 2019a	Low	Unclear	Low	Low	Low	Low	Low	Low
Higuchi 2019b	Unclear	Unclear	High	High	Low	Low	Low	High
Hirayasu 2010	Unclear	Unclear	Low	Low	Unclear	Unclear	Low	Unclear
Honer 2010	Low	Low	Unclear	Unclear	Low	Low	Low	Low
ILPB202	Unclear	Unclear	Unclear	Unclear	Unclear	Unclear	Unclear	Unclear
Ishigooka 2018	Low	Low	Unclear	Unclear	Low	Low	Low	Low
Iwata 2019	Low	Low	Low	Low	Low	Low	Low	Low
Iyo 2021	Low	Low	Low	Low	Low	Low	Low	Low
Kahn 2007	Unclear	Unclear	Low	Low	Low	Low	Low	Low
Kane 2002	Unclear	Unclear	Unclear	Unclear	Low	Low	Low	Unclear
Kane 2003	Low	Low	Low	Low	Low	Low	Low	Low
Kane 2007	Low	Low	Low	Low	Low	Low	Low	Low

Study	D1	D2	D3	D4	D5	D6	D7	Overall
Kane 2010	Unclear	Unclear	Low	Low	Unclear	Low	Low	Unclear
Kane 2014	Low	Low	Low	Low	Low	Low	Low	Low
Kane 2015a	Low	Low	Low	Low	Low	Low	Low	Low
Kane 2015b	Unclear	Unclear	Unclear	Unclear	Low	Low	Low	Unclear
Keck 1998	Low	Unclear	Unclear	Unclear	Low	Low	Low	Unclear
King 1998	Low	Low	Unclear	Unclear	Low	Low	Low	Low
Kinon 2006	Low	Low	Low	Low	Low	Low	Low	Low
Kinon 2011	Low	Low	Low	Low	High	Low	Low	Unclear
Kinoshita 2016	Low	Low	Low	Low	Low	Low	Low	Low
Klieser 1989	Low	Unclear	Unclear	Unclear	Unclear	Low	Low	Unclear
Kramer 2010	Low	Low	High	High	Low	Low	Low	High
Landbloom 2016	Low	Unclear	Low	Low	Low	Low	Low	Low
Lane 2001	Unclear	Unclear	Unclear	Unclear	Low	Unclear	Unclear	Unclear
Lauriello 2008	Low	Low	Unclear	Unclear	Low	Low	Low	Low
Liebermann 2015	Low	Low	Low	Low	Low	Low	Low	Low
Lindenmayer 2008	Low	Low	Low	Low	Low	Low	Low	Low
Lindenmayer 2011	Unclear	Unclear	Low	Low	Low	High	Low	Unclear
Litman 2016	Low	Unclear	Low	Low	Low	Low	Low	Low
Litman 2014	Low	Low	Low	Low	Low	Low	Low	Low
Loebel 2013	Low	Low	Low	Low	Low	Low	Low	Low
Loebel 2015	Low	Low	Low	Low	Low	Low	Low	Low
Louza 1988	Unclear	Unclear	Low	Low	High	Low	High	High
Marder 1994	Low	Low	Low	Low	Low	Low	Low	Low
Marder 2007c	Low	Low	Low	Low	Low	Low	Low	Low
Matsumoto 2018	Low	Low	Unclear	Unclear	Low	Low	Low	Low
McEvoy 1991	Low	Low	Low	Low	Low	Unclear	Unclear	Unclear
McEvoy 2007	Low	Low	Low	Low	Low	Low	Low	Low
Meltzer 2004	Low	Unclear	Unclear	Unclear	Low	Low	Low	Unclear

Study	D1	D2	D3	D4	D5	D6	D7	Overall
Meltzer 2007	Unclear	Unclear	Unclear	Unclear	Low	Unclear	Unclear	Unclear
Meltzer 2011	Low	Low	Low	Low	Low	Low	Low	Low
Meltzer 2012	Unclear	Unclear	Unclear	Unclear	Low	Low	Low	Unclear
Meltzer 2014	Unclear	Unclear	Unclear	Unclear	Unclear	Unclear	Low	Unclear
Meltzer 2015	Unclear	Unclear	Low	Low	Low	Low	Low	Low
Meltzer 2020	Unclear	Unclear	Low	Low	Low	Low	Low	Low
Merlo 2000	Unclear	Unclear	Unclear	Unclear	Unclear	Unclear	Low	Unclear
Nakamura 2009	Low	Low	Low	Low	Low	Low	Low	Low
Nakamura 2016	Unclear	Unclear	High	High	Low	Low	Low	High
Nasrallah 2010	Low	Low	Low	Low	Low	Low	Low	Low
Nasrallah 2013	Low	Low	Low	Low	Low	Low	Low	Low
Nasser 2016	Low	Low	Low	Low	Low	Low	Low	Low
NCT00563706	Unclear	Unclear	Low	Low	Low	Low	Low	Low
NCT00905307	Low	Low	Low	Low	Low	Low	Low	Low
NCT01625000	Unclear	Unclear	Unclear	Unclear	High	Unclear	Low	Unclear
NCT02469155	Unclear	Unclear	Low	Low	Low	Unclear	Unclear	Unclear
NCT04512066	Unclear	Unclear	Unclear	Unclear	Unclear	Unclear	Unclear	Unclear
Ogasa 2012	Low	Low	Low	Low	Low	Low	Low	Low
Oosthuizen 2004	Unclear	Unclear	Low	Low	Low	Low	Low	Low
Pandina 2010	Low	Low	Low	Low	Unclear	Unclear	Low	Low
Patil 2007	Unclear	Unclear	Unclear	Unclear	Low	High	Low	Unclear
Peuskens 1995	Low	Low	Low	Low	Low	Low	Low	Low
Potkin 2003	Unclear	Unclear	Unclear	Unclear	Low	Low	Low	Unclear
Potkin 2007	Unclear	Unclear	Low	Low	Low	Low	Low	Low
Potkin 2008a	Low	Low	Low	Low	Low	Low	Low	Low
Potkin 2008b	Low	Low	Low	Low	Low	Low	Low	Low
Potkin 2008c	Low	Low	Low	Low	Low	Low	Low	Low
Potkin 2015	Low	Low	Low	Low	Low	Low	Low	Low

Study	D1	D2	D3	D4	D5	D6	D7	Overall
Potkin 2020	Unclear	Unclear	Low	Low	Low	Low	Low	Low
Puech 1998	Low	Low	Low	Low	Low	Low	Low	Low
Rifkin 1991	Unclear	Unclear	Low	Low	Low	Low	Low	Low
Saito 2020	Low	Low	Low	Low	Low	Low	Low	Low
Sarin 2004	Unclear	Unclear	High	High	High	Low	Low	High
Schmidt 2014	Low	Low	Low	Low	Low	High	Low	Unclear
Shen 2014	Low	Low	Unclear	Unclear	High	High	Low	High
Simpson 1967	Low	Low	Low	Low	Low	High	Low	Unclear
Simpson 1999	Unclear	Unclear	Low	Low	Low	Low	Low	Low
Singh 2011	Low	Low	Low	Low	Unclear	Low	Low	Low
Study 104	Unclear	Unclear	Unclear	Unclear	Low	Unclear	Unclear	Unclear
Study 115 2000	Low	Unclear	Unclear	Unclear	Low	Unclear	Low	Unclear
Study 93202 2002	Unclear	Unclear	Unclear	Unclear	Low	Low	Low	Unclear
Study 94202 2002	Unclear	Unclear	Low	Low	Low	Low	Low	Low
Study RIS-USA-72 1996	Unclear	Unclear	Low	Low	Low	Low	Low	Low
Takahashi 2013	Unclear	Unclear	Low	Low	Low	Low	Low	Low
van Kammen 1996	Low	Low	Unclear	Unclear	Low	Low	Low	Low
Walling 2019	Low	Low	Low	Low	Low	Low	Low	Low
Zborowski 1995	Low	Low	Low	Low	Low	Low	Low	Low
Zimbroff 1997	Low	Low	Low	Low	Low	Low	Low	Low

D1 = random sequence generation; D2 = allocation concealment; D3 = blinding of participants and personnel; D4 = blinding of outcome assessment; D5 = incomplete outcome data; D6 = selective reporting; D7 = other sources of bias

Summary plot for risk of bias

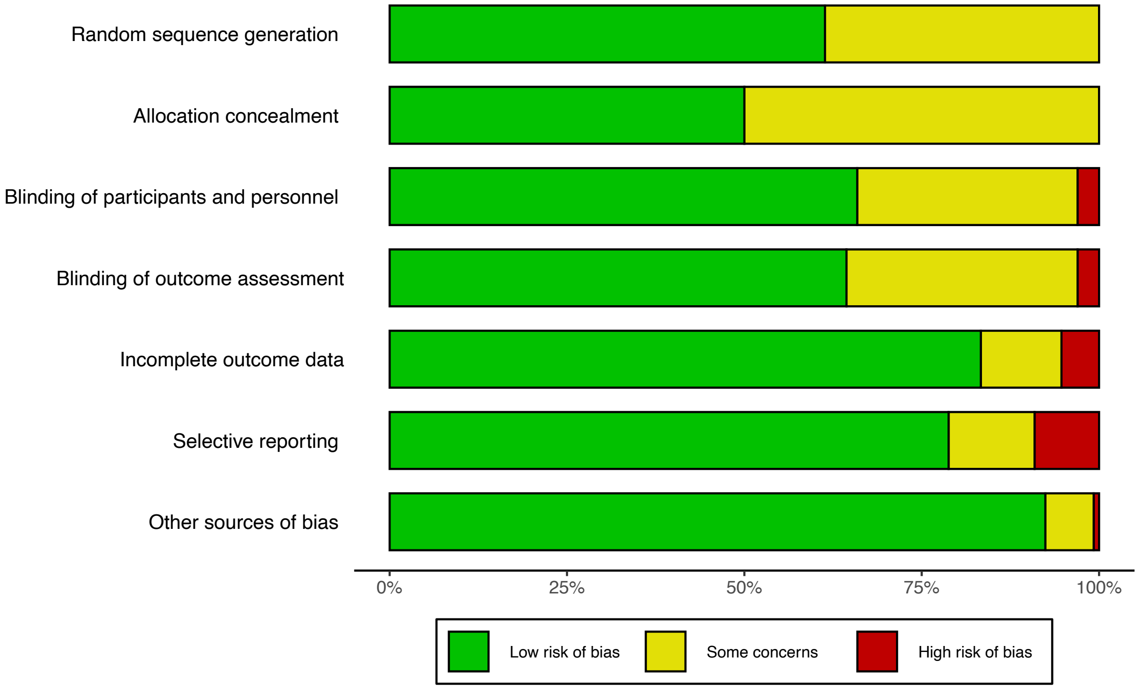

eAppendix 8: Summary of statistical results

Adults

Drug	Number of studies	Number of arms	Number of participants	coeff_1	coeff_1_pval	coeff_2	coeff_2_pval	ed50	ed50_es	ed95	ed95_es	wald_test	walt_test_pval	vpc_summary
Amisulpride	1	4	248	0.001	0.01	-0.001	0.01	264.7	0.32	536.7	0.61	7.831	0.02	not estimable
Aripiprazole	12	33	3,228	0.057	0.00	-0.029	0.00	4.3	0.23	10.2	0.44	85.436	0.00	median=23.3% IQR[10.5%, 32.7%]
Asenapine	6	17	2,206	0.048	0.00	-0.040	0.00	4.5	0.21	10.6	0.39	37.696	0.00	median=36.4% IQR[26.1%, 54.5%]
Blonanserin	2	7	817	0.082	0.13	-0.042	0.37	4.2	0.33	14.5	0.62	23.155	0.00	median=71.3% IQR[68.7%, 74.3%]
Brexpiprazole	4	17	2,131	0.211	0.00	-0.202	0.01	0.7	0.15	3.5	0.29	31.712	0.00	median=0% IQR[0%, 0%]
Cariprazine	6	20	2,146	0.101	0.00	-0.060	0.00	2.0	0.20	6.2	0.37	44.990	0.00	median=3.6% IQR[0.3%, 12.1%]
Clozapine	1	3	48	0.002	0.33	0.000	0.92	279.8	0.55	567.2	1.04	5.867	0.05	not estimable
Haloperidol	22	48	2,740	0.063	0.00	-0.039	0.00	4.2	0.25	12.4	0.48	129.567	0.00	median=0% IQR[0%, 0%]
Iloperidone	5	15	1,940	0.019	0.25	0.005	0.83	13.1	0.31	22.9	0.58	15.372	0.00	median=32.6% IQR[14.6%, 82%]

Drug	Number of studies	Number of arms	Number of participants	coeff_1	coeff_1_pval	coeff_2	coeff_2_pval	ed50	ed50_es	ed95	ed95_es	wald_test	walt_test_pval	vpc_summary
Lumateperone	3	9	1,168	0.007	0.16	-0.004	0.20	14.9	0.10	34.8	0.18	2.145	0.34	median=59.7% IQR[50.3%, 66.1%]
Lurasidone	11	32	3,593	0.008	0.00	-0.005	0.00	32.0	0.24	207.5	0.45	34.275	0.00	median=43.3% IQR[37.2%, 57.3%]
Olanzapine	22	52	4,807	0.067	0.00	-0.033	0.00	4.7	0.30	15.7	0.57	118.295	0.00	median=51.1% IQR[37.9%, 60.6%]
Paliperidone	11	36	4,103	0.103	0.00	-0.048	0.00	3.0	0.29	12.7	0.55	69.206	0.00	median=55.4% IQR[40.4%, 59.6%]
Quetiapine	9	32	2,978	0.001	0.00	0.000	0.03	260.8	0.25	1,021.5	0.47	14.776	0.00	median=70.4% IQR[49.6%, 75.7%]
Risperidone	28	70	6,531	0.169	0.00	-0.089	0.00	1.7	0.27	4.0	0.52	189.497	0.00	median=30.7% IQR[13.9%, 39.6%]
Sertindole	4	15	1,228	0.030	0.00	-0.012	0.17	8.2	0.25	19.4	0.47	36.111	0.00	median=0% IQR[0%, 0%]
Ziprasidone	7	22	1,345	0.003	0.03	-0.003	0.36	104.9	0.27	298.1	0.51	25.166	0.00	median=0.9% IQR[0.5%, 2.5%]

ed50 = effective dose 50%; ed50_es = effect size at ed50; ed95 = effective dose 95%; ed95_es = effect size at ed95; vpc_summary = summary of the variance partition coefficient

Children/adolescents

Drug	Number of studies	Number of arms	Number of participants	coeff_1	coeff_1_pval	coeff_2	coeff_2_pval	ed50	ed50_es	ed95	ed95_es	wald_test	walt_test_pval	vpc_summary
Aripiprazole	2	6	399	0.02	0.142	-0.017	0.449	8.3	0.16478073	25.3	0.31308339	6.847	0.033	median=0% IQR[0%, 0%]
Asenapine	1	3	228	0.071	0.062	-0.037	0.42	3.1	0.21613276	8.6	0.41065225	7.77	0.021	not estimable
Blonanserin	1	3	150	0.03	0.301	0.003	0.94	8.4	0.25697144	15.2	0.48824574	6.4	0.041	not estimable
Lurasidone	1	3	326	0.013	0.001	-0.01	0.033	19.1	0.25678437	40.2	0.4878903	16.359	0	not estimable
Paliperidone	1	4	200	0.169	0.012	-0.162	0.04	1.6	0.27778394	3.7	0.52778949	8.262	0.016	not estimable
Quetiapine	2	5	311	0.001	0.021	-0.001	0.186	188.2	0.2268909	440.7	0.4310927	9.65	0.008	median=0.2% IQR[0.1%, 1.4%]
Risperidone	2	5	413	0.295	0	-0.278	0.02	1.4	0.41111673	3.3	0.78112179	37.107	0	median=0% IQR[0%, 0%]

ed50 = effective dose 50%; ed50_es = effect size at ed50; ed95 = effective dose 95%; ed95_es = effect size at ed95; vpc_summary = summary of the variance partition coefficient

eAppendix 9: Heterogeneity assessments

Heterogeneity assessments with the variance-partition-coefficient (VPC) for the primary analyses.

Amisulpride

<u>Adults:</u>

Only one study was included, thus no heterogeneity was assessed.

<u>Children/adolescents:</u>

No study was available.

Aripiprazole

<u>Adults:</u>

Median VPC was under 50%, indicating low level of heterogeneity.

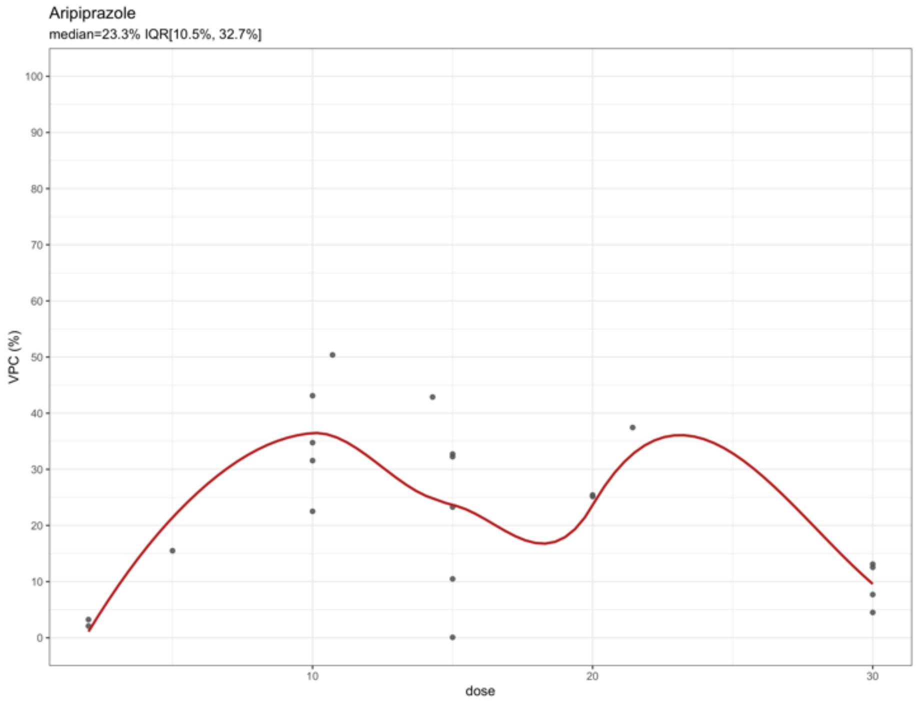

Children/adolescents:

Median VPC was 0%, indicating low level of heterogeneity.

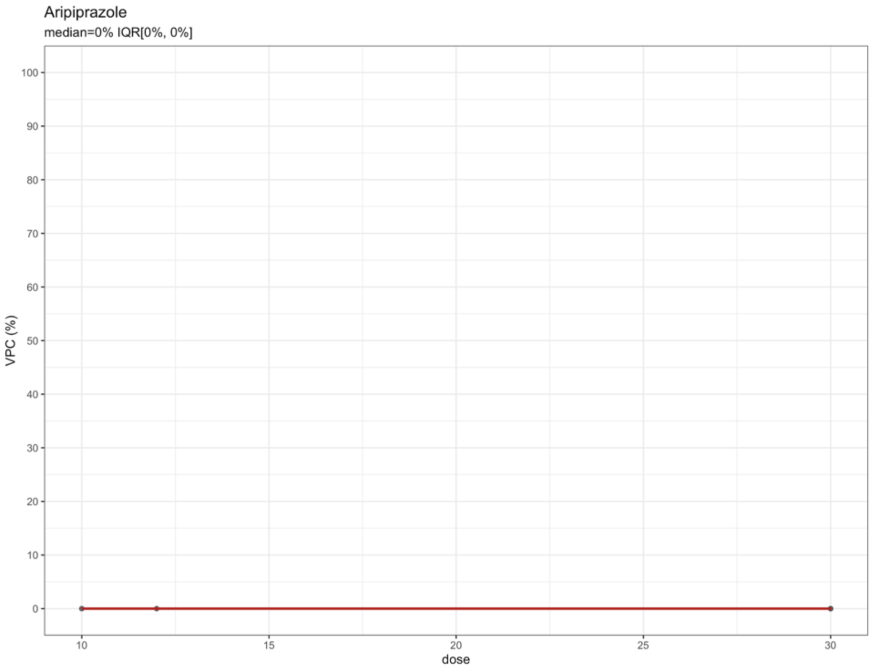

Asenapine

Adults:

Median VPC was under 50%, indicating low level of heterogeneity.

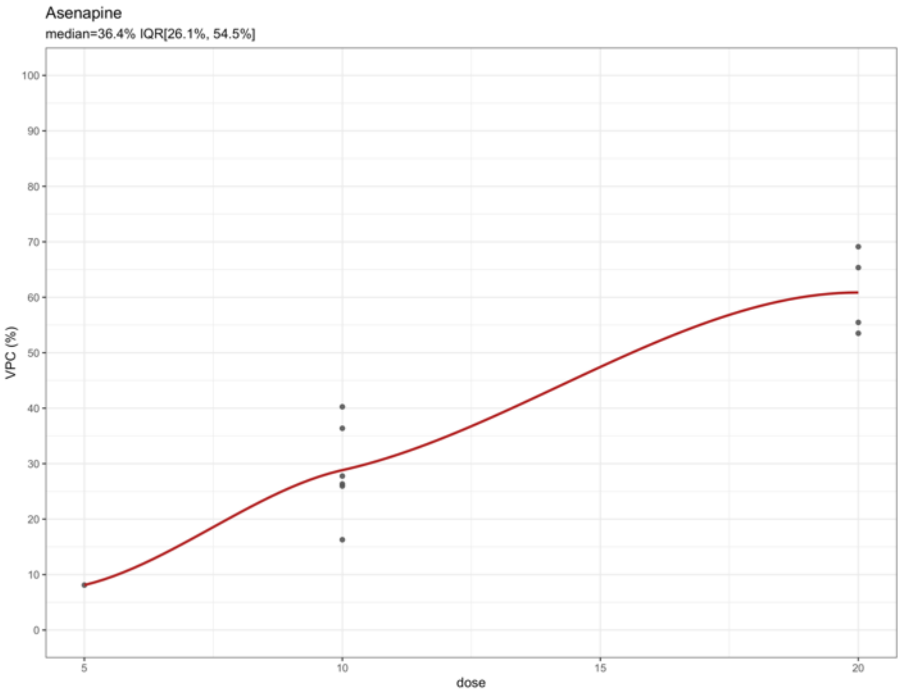

Children/adolescents:

1 study was available, and heterogeneity was not assessed.

Blonanserin

Adults:

Median VPC was 71.3%, indicating moderate level of heterogeneity.

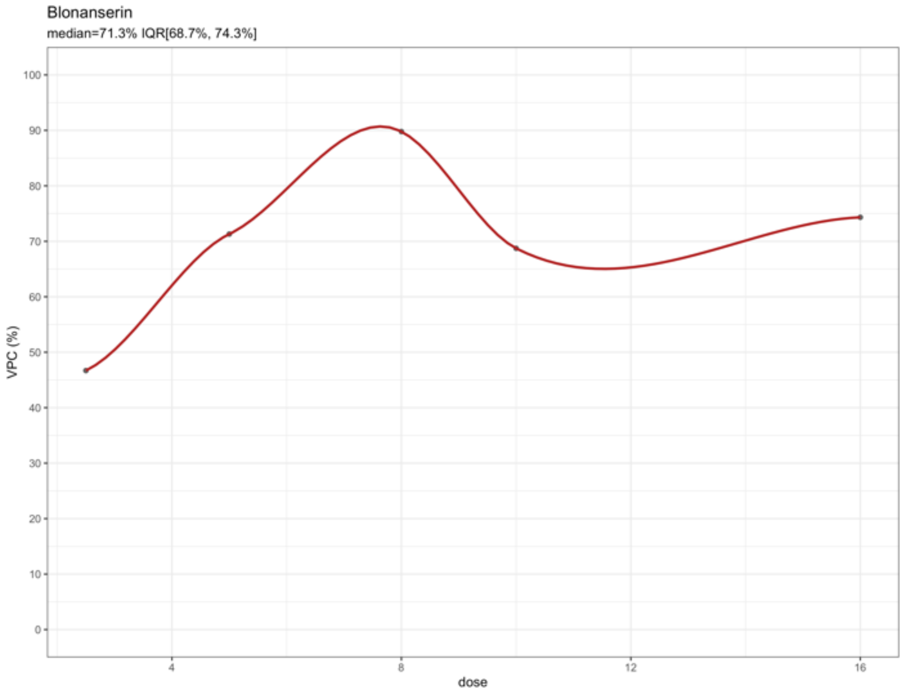

Children/adolescents:

1 study was available, and heterogeneity was not assessed.

Brexpiprazole

Adults:

Median VPC was 0%, indicating no heterogeneity.

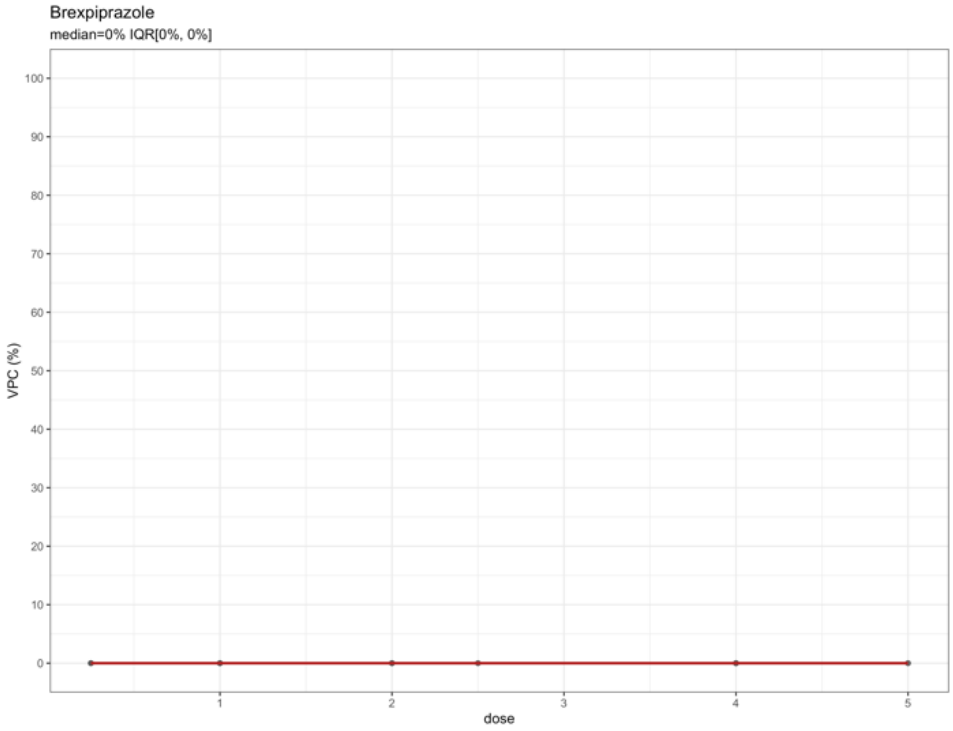

Children/adolescents:

No study was available.

Cariprazine

Adults:

Median VPC was under 50%, indicating low level of heterogeneity.

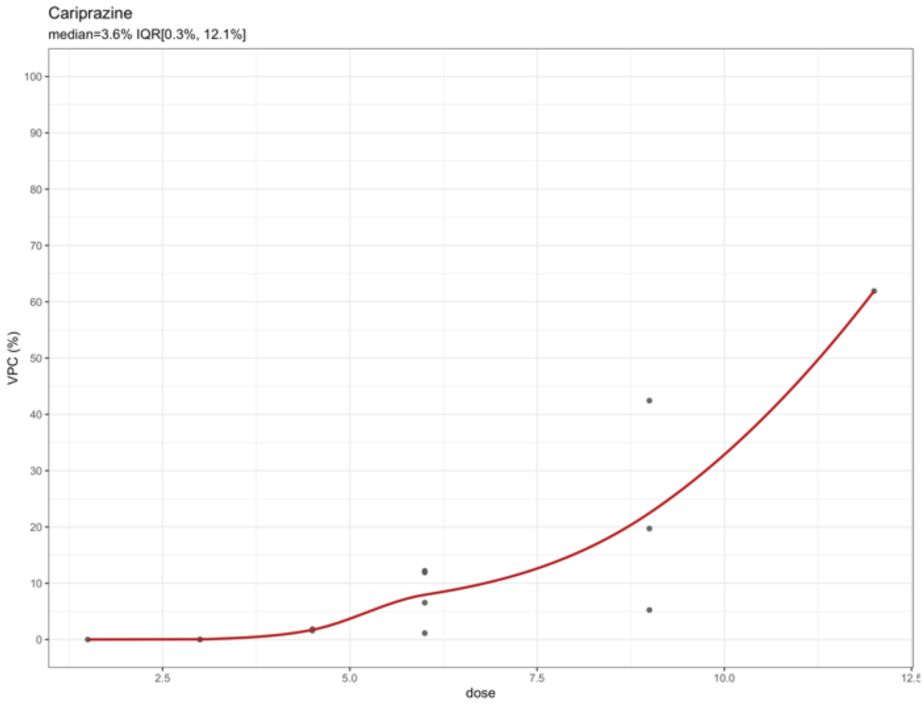

Children/adolescents:

No study was available.

Clozapine

Adults:

Only one study was available, and heterogeneity was not assessed.

Children/adolescents:

No study was available.

Haloperidol

<u>Adults:</u>

Median VPC was 0%, indicating no heterogeneity.

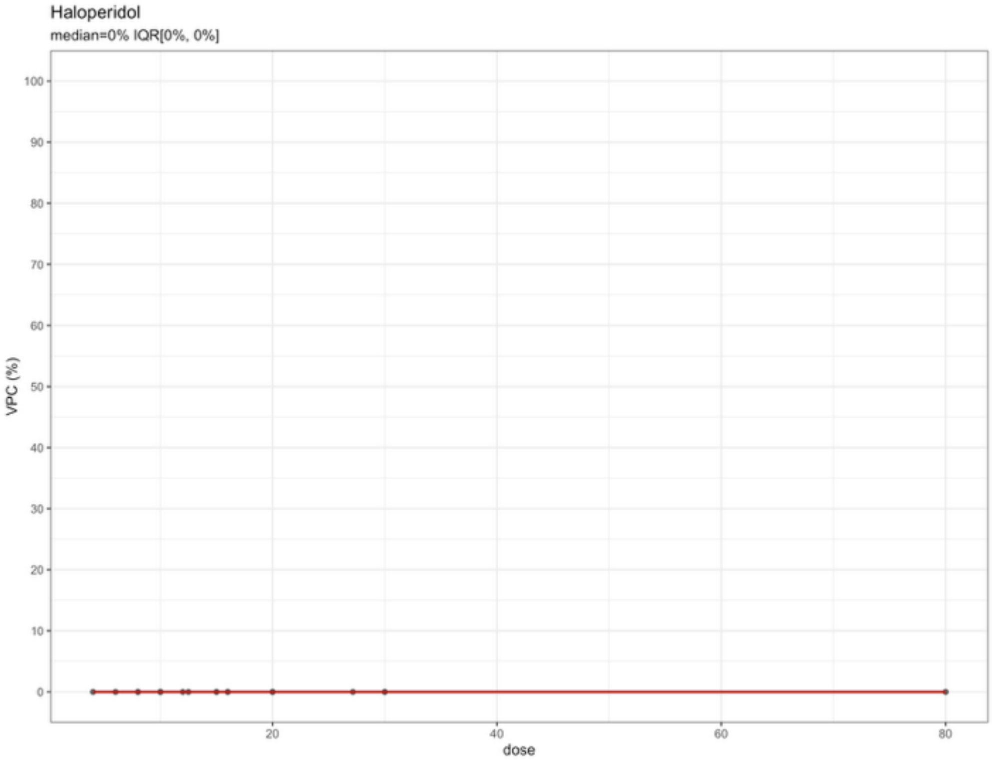

<u>Children/adolescents:</u>

No study was available.

Iloperidone

Adults:

Median VPC was under 50%, indicating low level of heterogeneity.

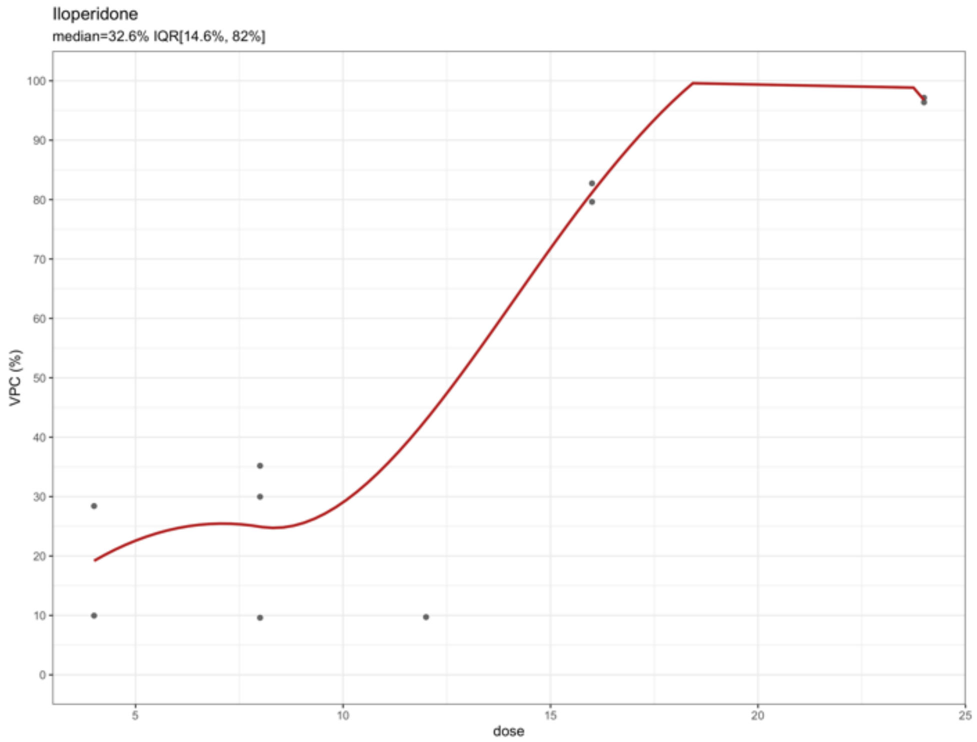

Children/adolescents:

No study was available.

Lumateperone

Adults:

Median VPC was 59.7%, indicating moderate level of heterogeneity.

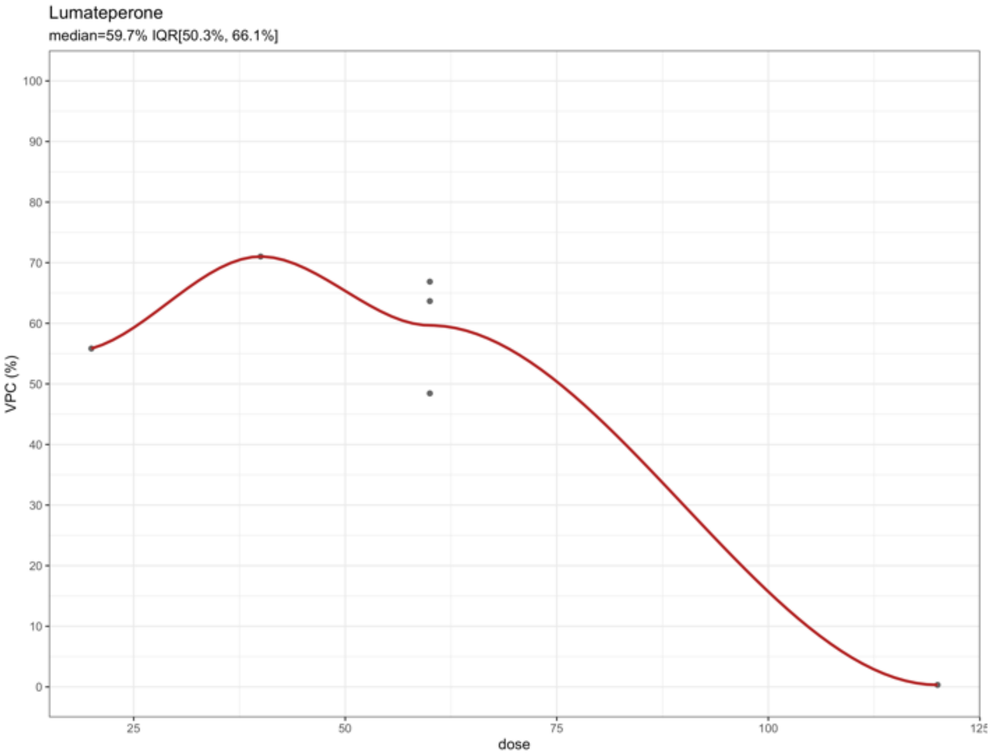

Children/adolescents:

No study was available.

Lurasidone

<u>Adults:</u>

Median VPC was under 50%, indicating low level of heterogeneity.

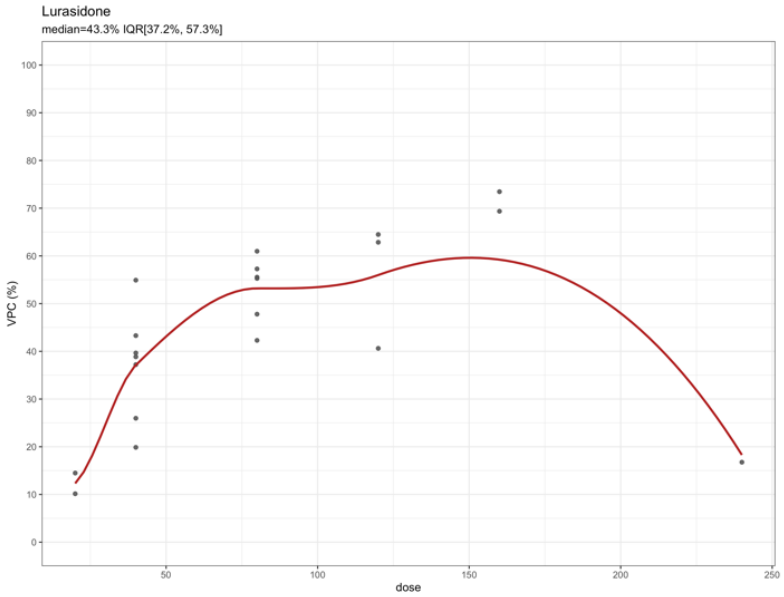

<u>Children/adolescents:</u>

1 study was available, and heterogeneity was not assessed.

Olanzapine

<u>Adults:</u>

Median VPC was 51.1%, indicating moderate level of heterogeneity.

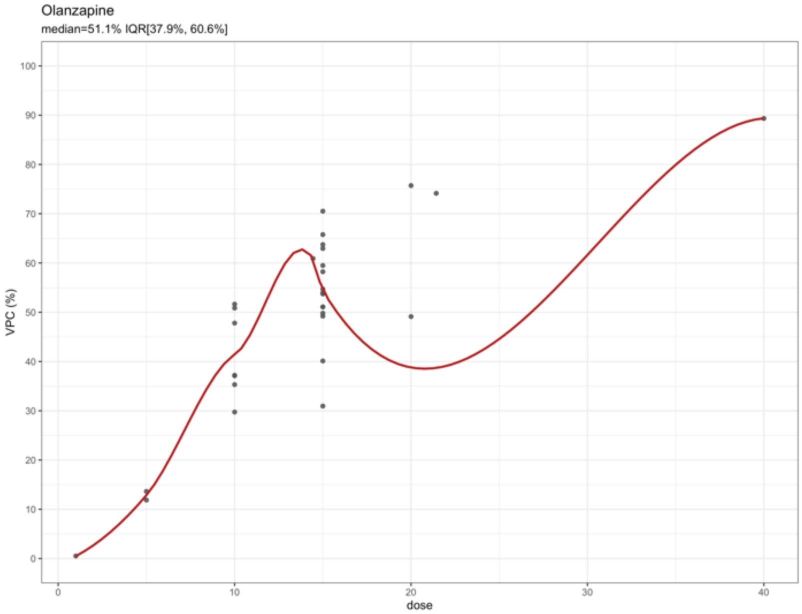

<u>Children/adolescents:</u>

No study was available.

Paliperidone

Adults:

Median VPC was 55.4%, indicating moderate level of heterogeneity.

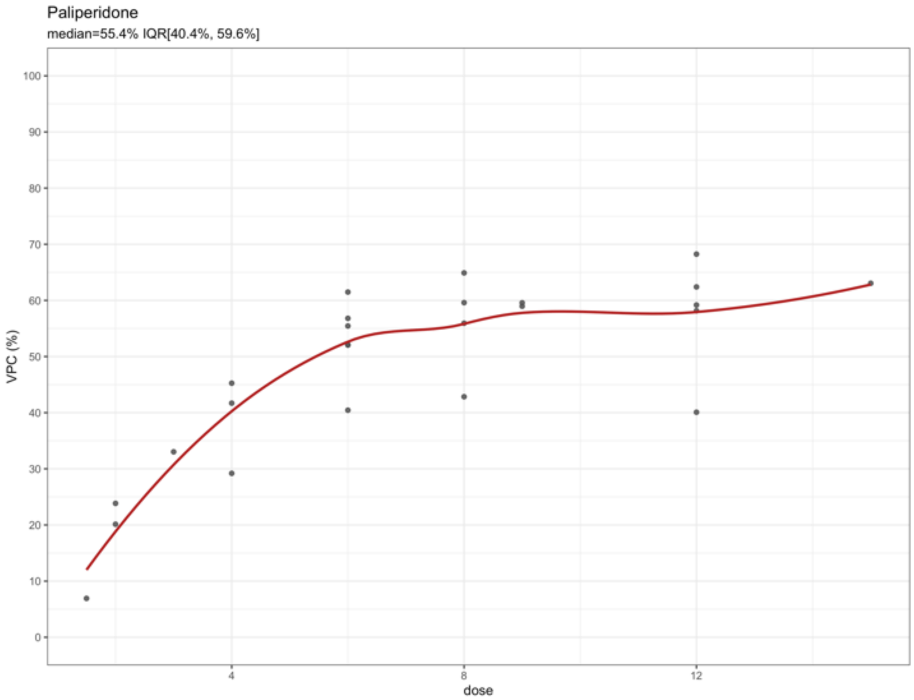

Children/adolescents:

1 study was available, and heterogeneity was not assessed.

Quetiapine

Adults:

Median VPC was 70.4%, indicating moderate level of heterogeneity.

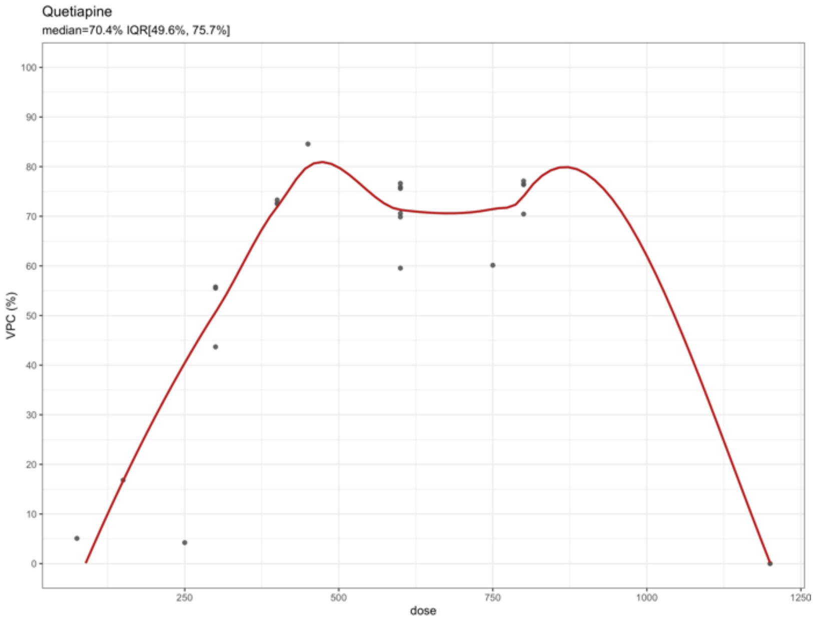

Children/adolescents:

Median VPC was 0.2%, indicating low level of heterogeneity.

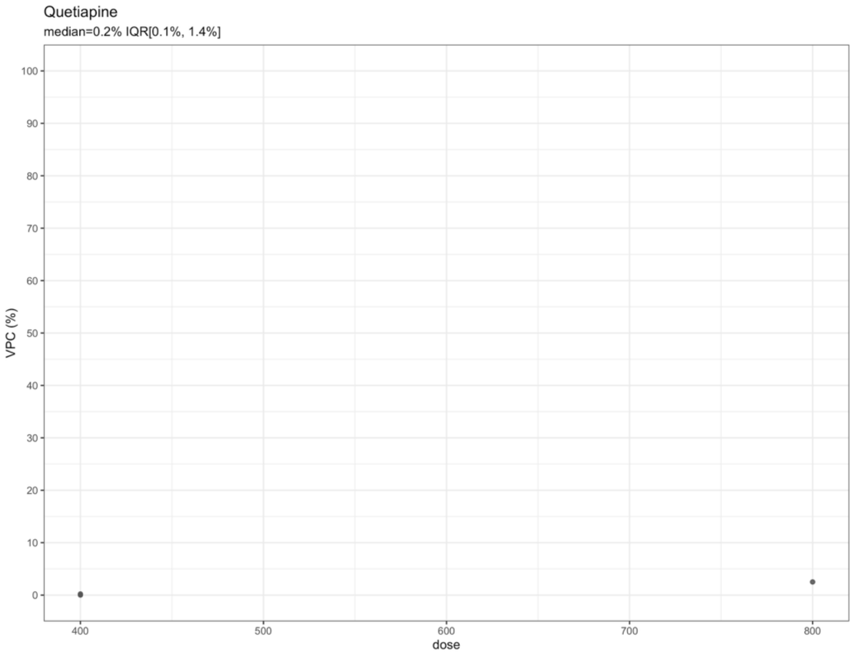

Risperidone

<u>Adults:</u>

Median VPC was under 50%, indicating low level of heterogeneity.

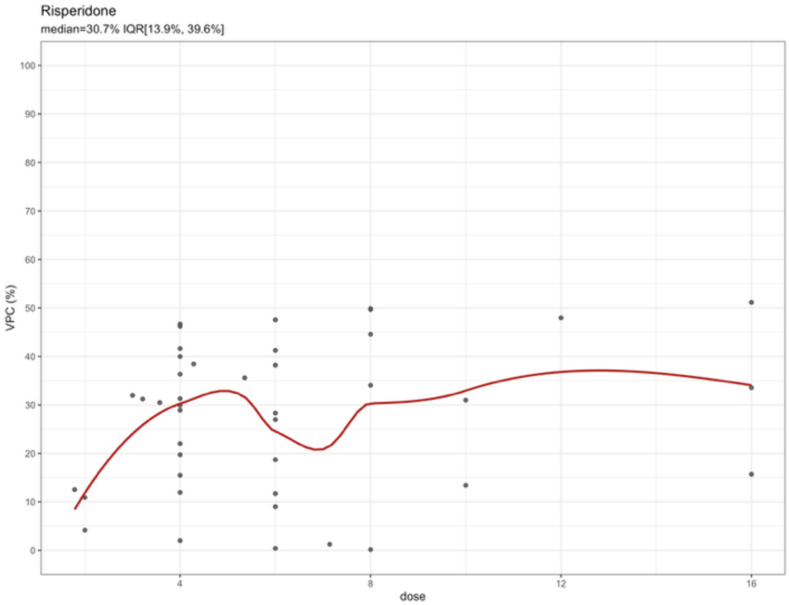

<u>Children/adolescents:</u>

Median VPC was 0%, indicating low level of heterogeneity.

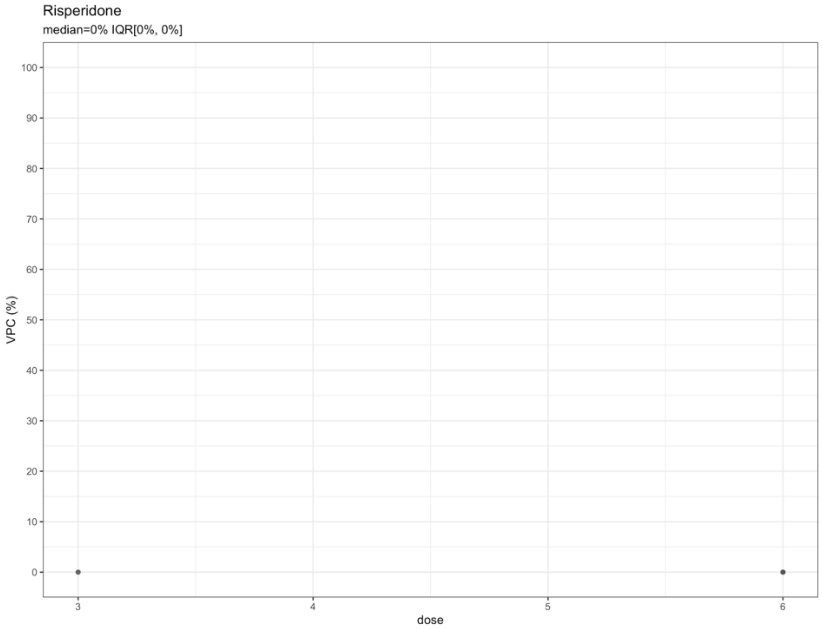

Sertindole

Adults:

Median VPC was 0%, indicating no heterogeneity.

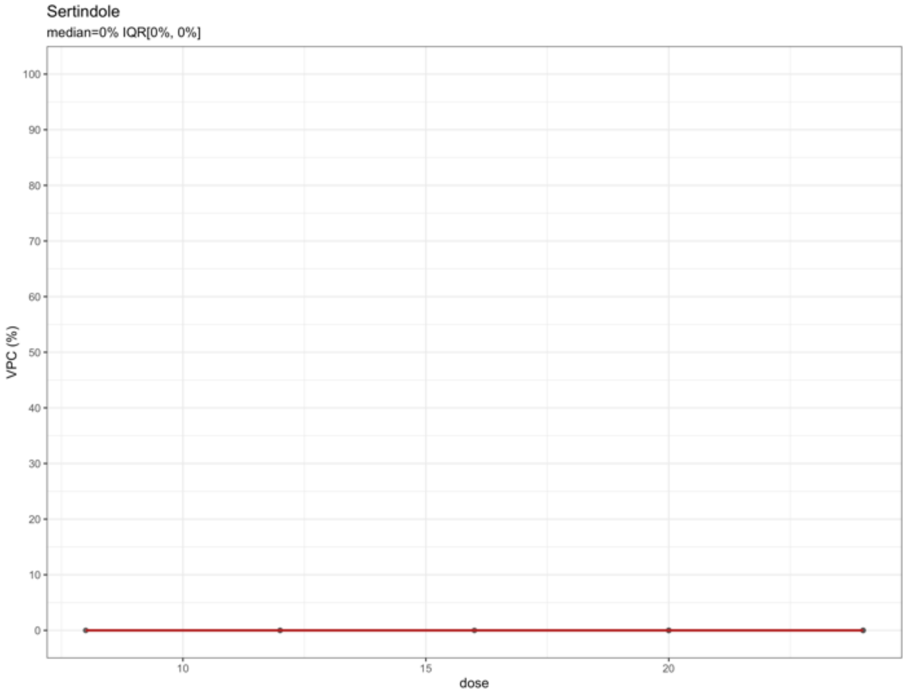

Children/adolescents:

No study was available.

Ziprasidone

Adults:

Median VPC was under 50%, indicating low level of heterogeneity.

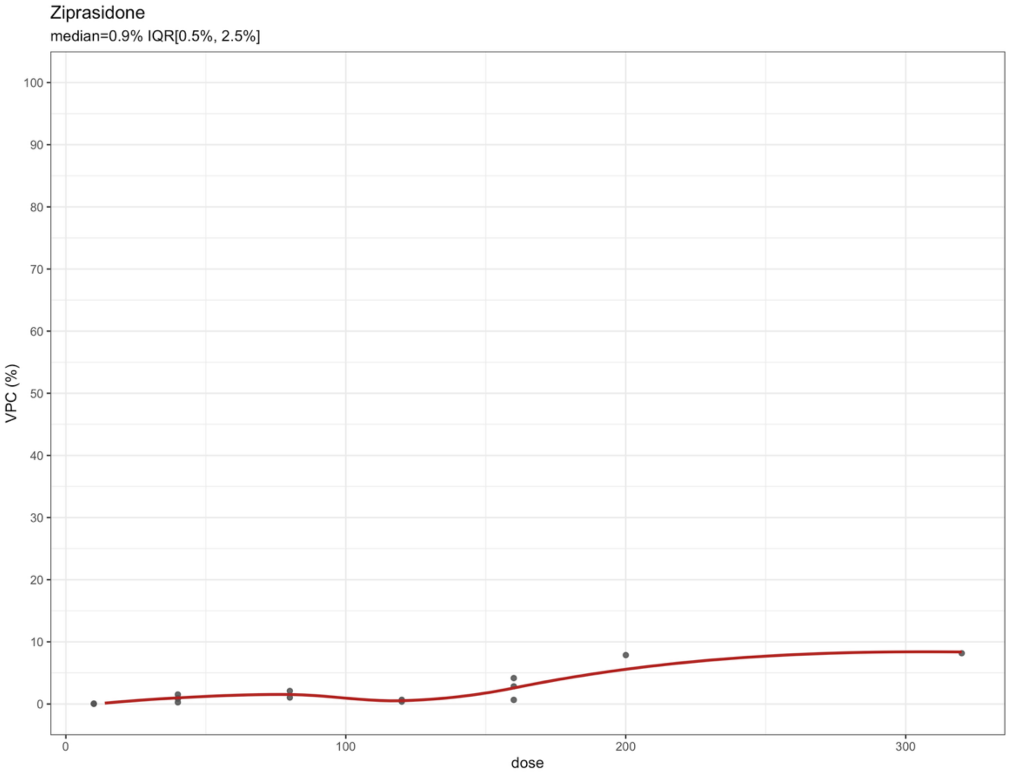

Children/adolescents:

Not study was available.

Antipsychotic pooled using risperidone dose equivalents

The VPS in the dose-response meta-analysis, using risperidone dose equivalents based on the adult ED95 values, are presented below.

<u>Adults:</u>

Median VPS was under 50%, indicating low level of heterogeneity.

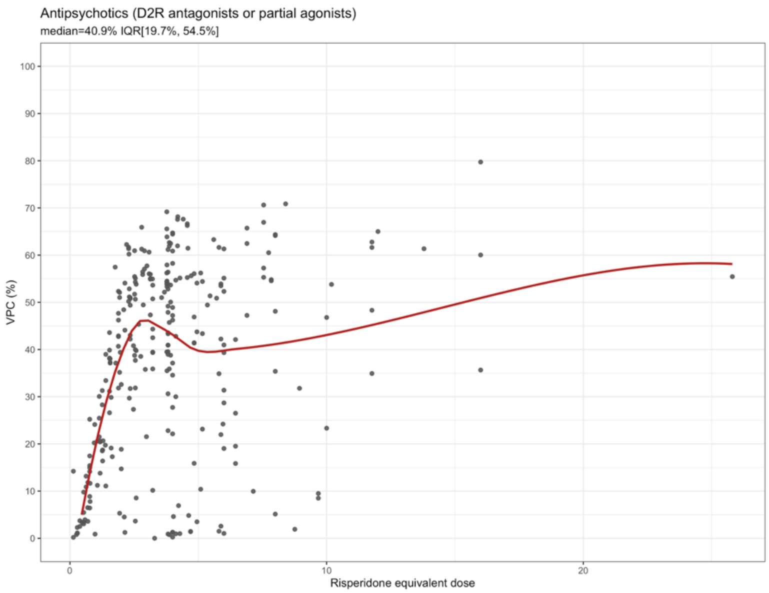

<u>Children/adolescents:</u>

Median VPS was under 50%, indicating low level of heterogeneity.

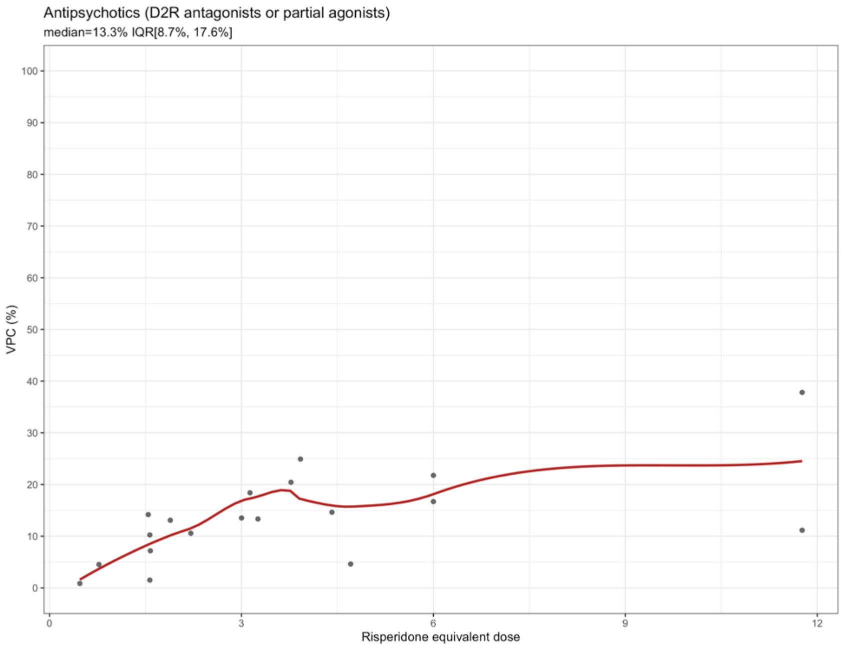

eAppendix 10: Small-study effects and publication bias

We present below the funnel plots and Egger's test for the comparison of antipsychotics versus placebo, as well as the results of the dose-response meta-regression for small-study effects in adult participant, when there were more than 10 studies. There were fewer than 10 studies available for other antipsychotics and for all antipsychotics in children/adolescents.

Aripiprazole

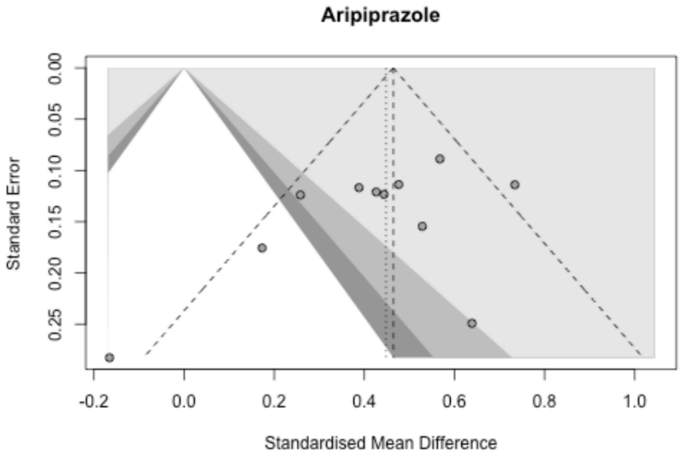

No clear asymmetry in the funnel plot is observed.

Egger's test: t = -1.64, df = 9, p-value = 0.136.

Dose-response meta-regression using the squared root of the sample size: X2 = 1.3, df = 2, P(> X2) = 0.51.

Haloperidol

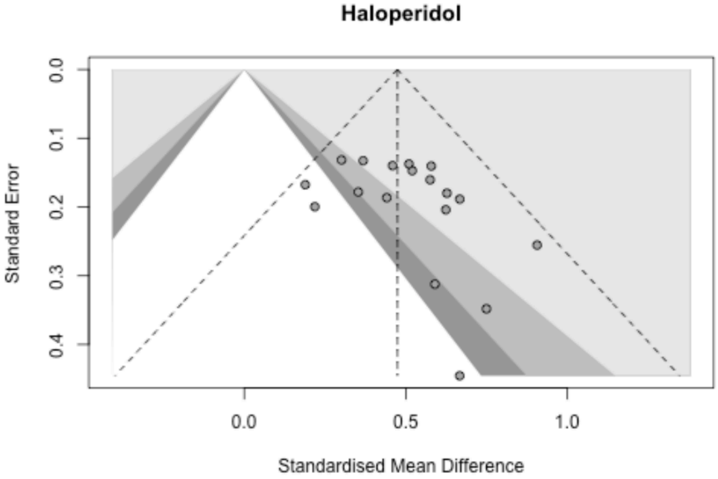

Some asymmetry can be observed in the funnel plot, although the dose-response meta-regression did not indicate clear small-study effects.

Egger's test: t = 1.75, df = 16, p-value = 0.0997

Dose-response meta-regression using the squared root of the sample size: X2 = 1.3, df = 2, P(> X2) = 0.53

Lurasidone

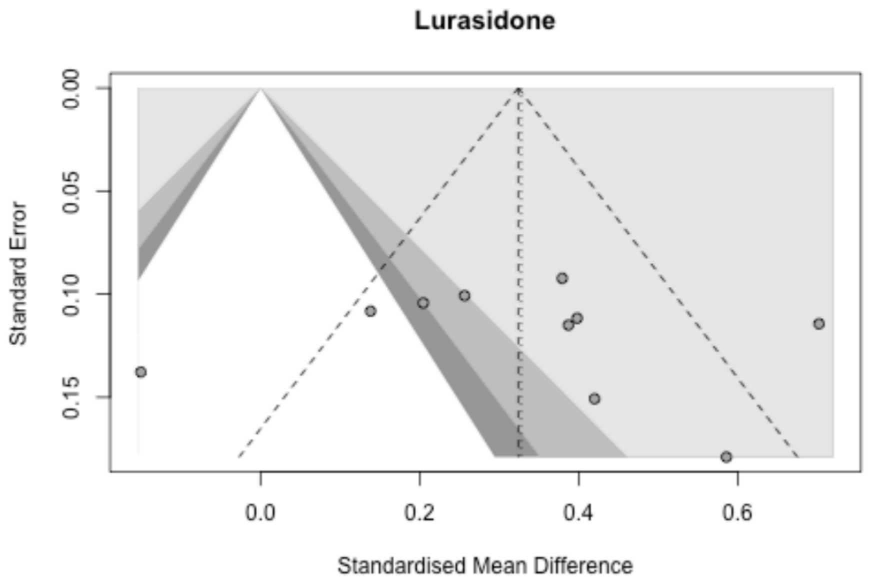

No clear asymmetry in the funnel plot is observed.

Egger's test: t = 0.22, df = 8, p-value = 0.831.

Dose-response meta-regression using the squared root of the sample size: X2 = 0.35, df = 2, P(> X2) = 0.84.

Olanzapine

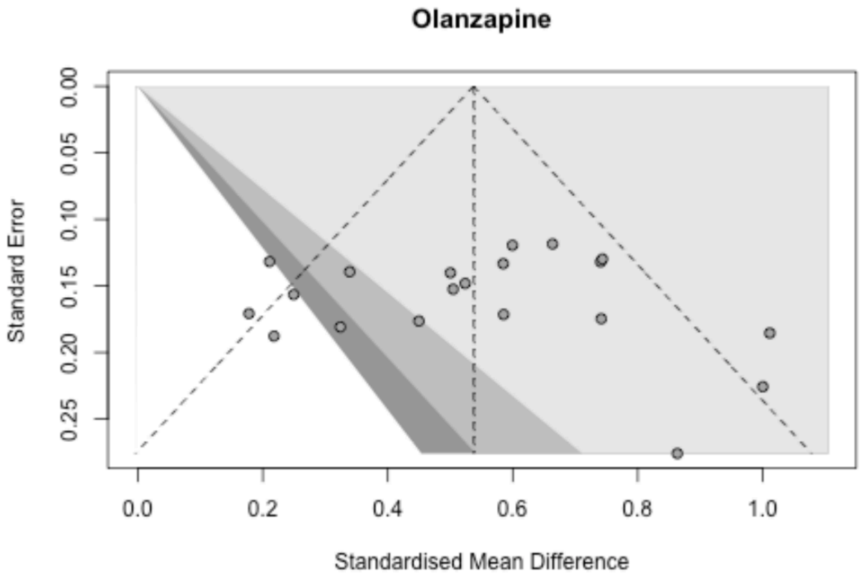

No clear asymmetry in the funnel plot is observed.

Egger's test: t = 0.40, df = 18, p-value = 0.690.

Dose-response meta-regression using the squared root of the sample size: X2 = 0.42, df = 2, P(> X2) = 0.81.

Paliperidone

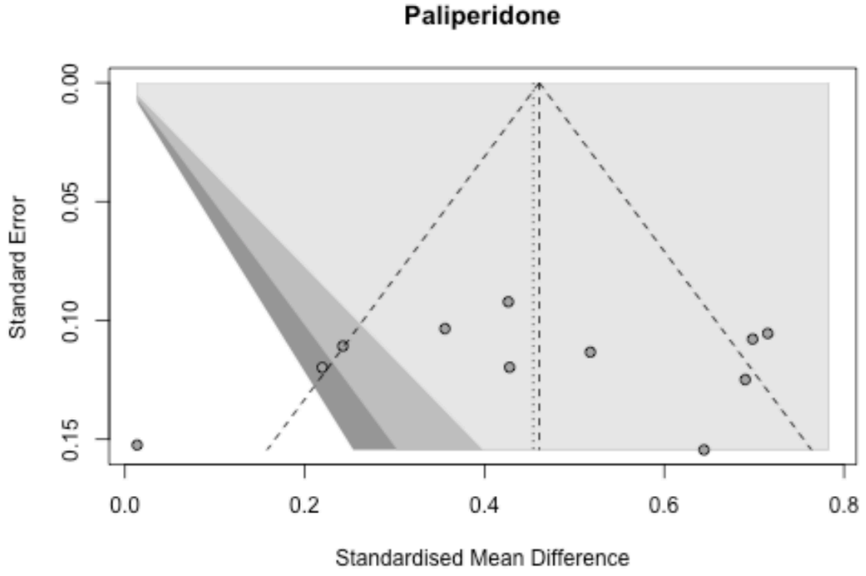

No clear asymmetry in the funnel plot is observed.

Egger's test: t = -0.51, df = 9, p-value = 0.625.

Dose-response meta-regression using the squared root of the sample size: X2 = 1.5, df = 2, P(> X2) = 0.48.

Risperidone

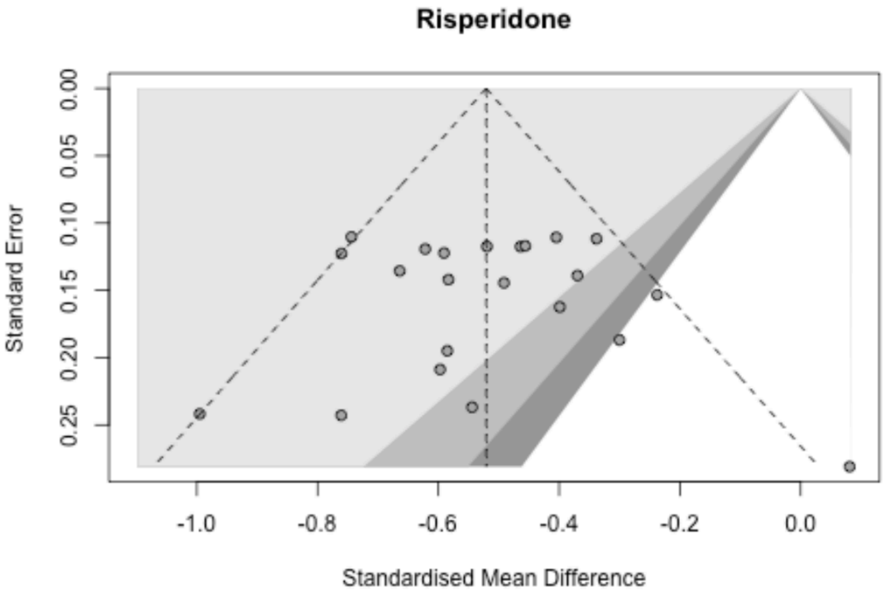

No clear asymmetry in the funnel plot is observed.

Egger's test: t = 0.21, df = 20, p-value = 0.833.

Dose-response meta-regression using the squared root of the sample size: X2 = 1.9, df = 2, P(> X2) = 0.39

eAppendix 11: Sensitivity analyses

Different formulations

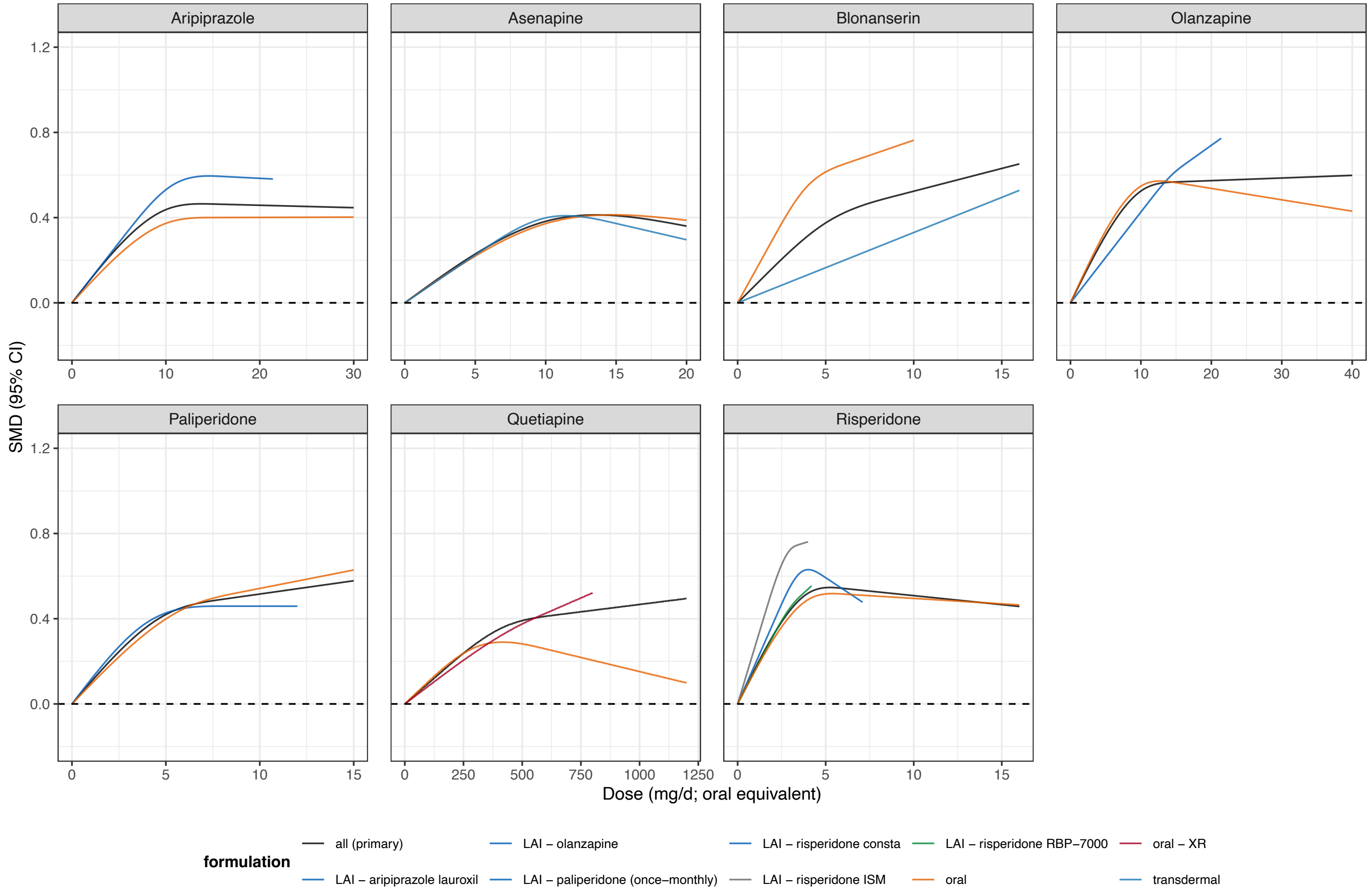

Different knot locations

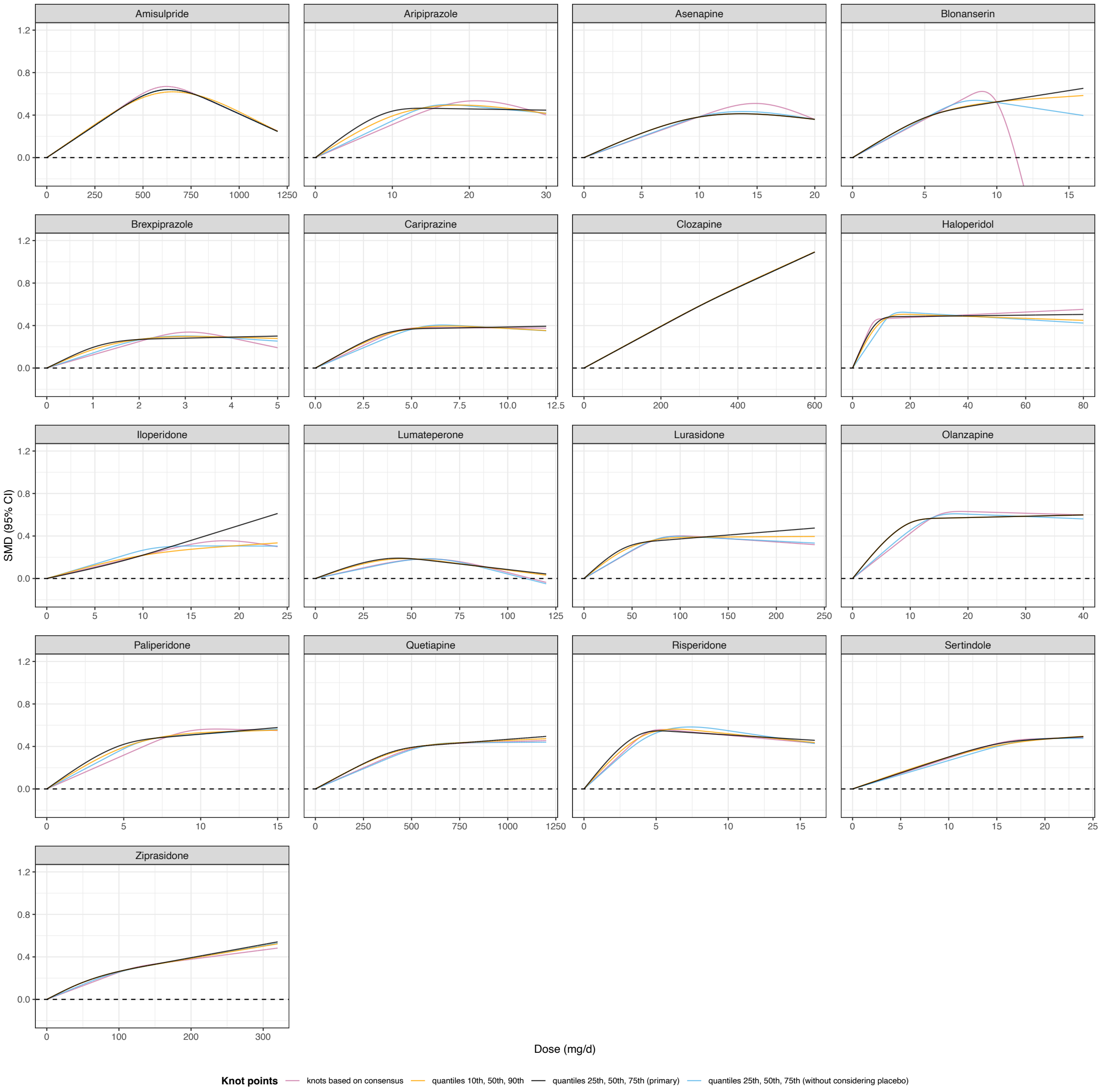

Different subgroups

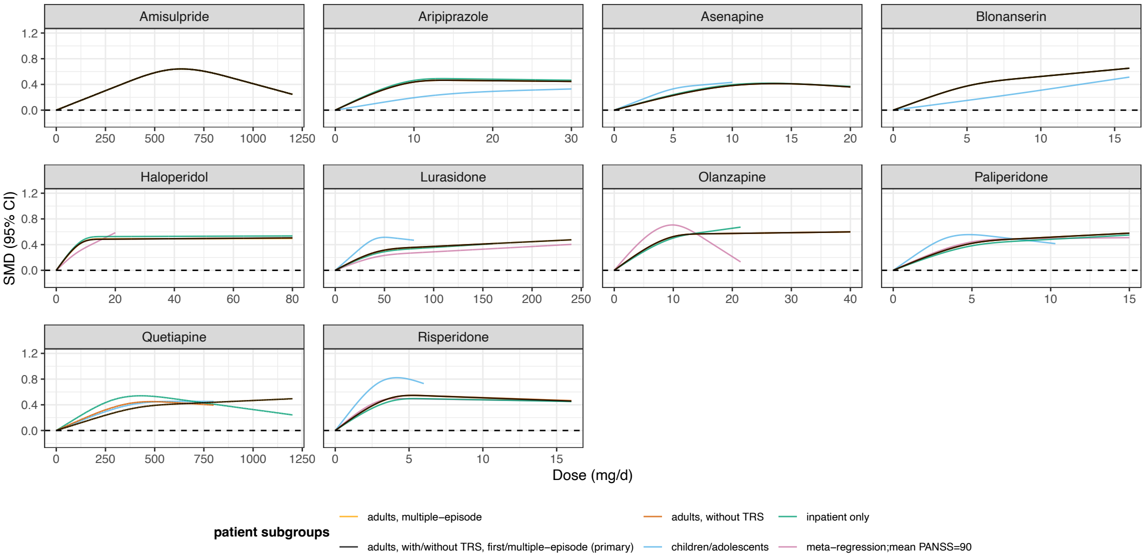

Other sensitivity analyses

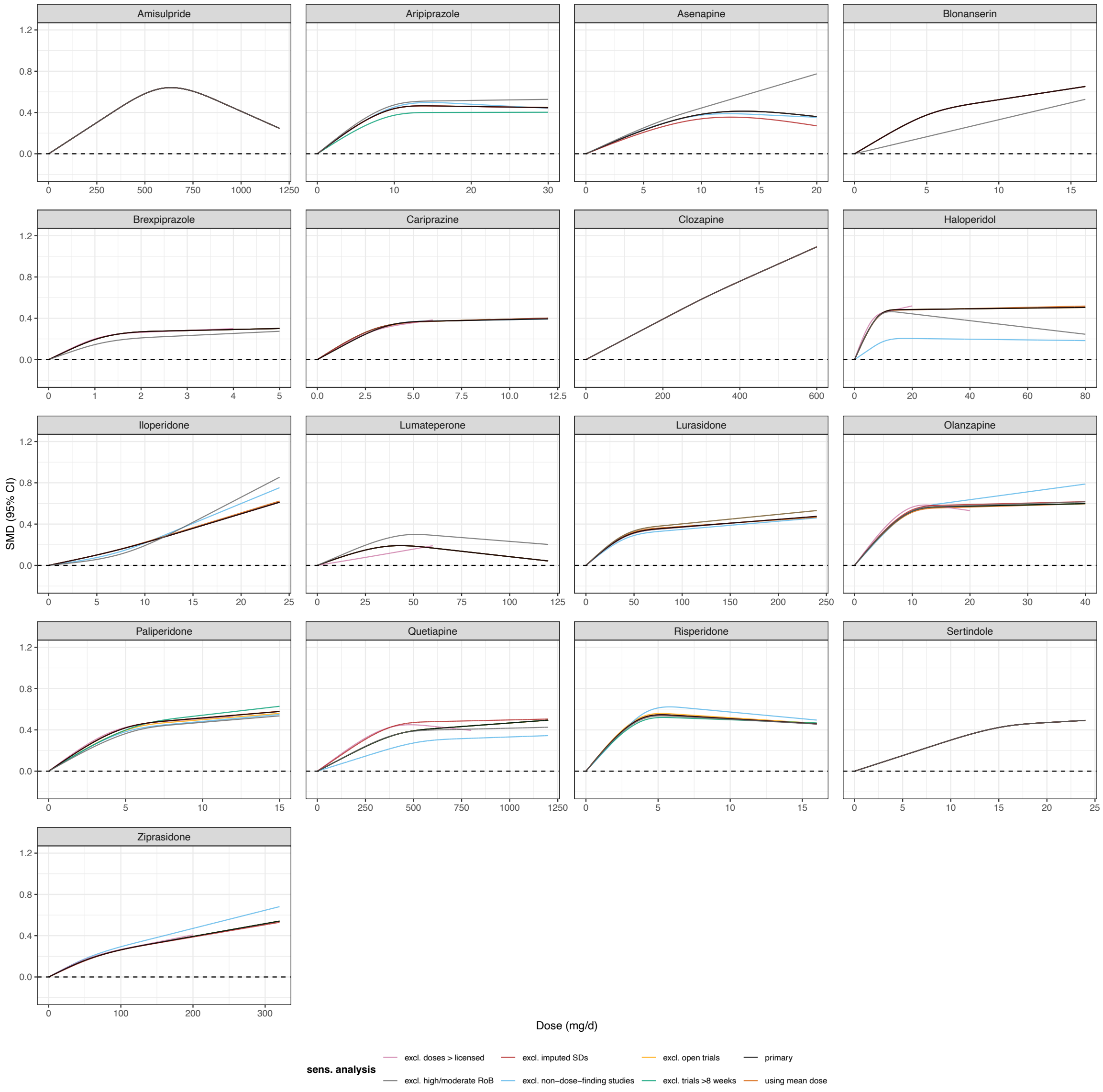

Using different pooling methods

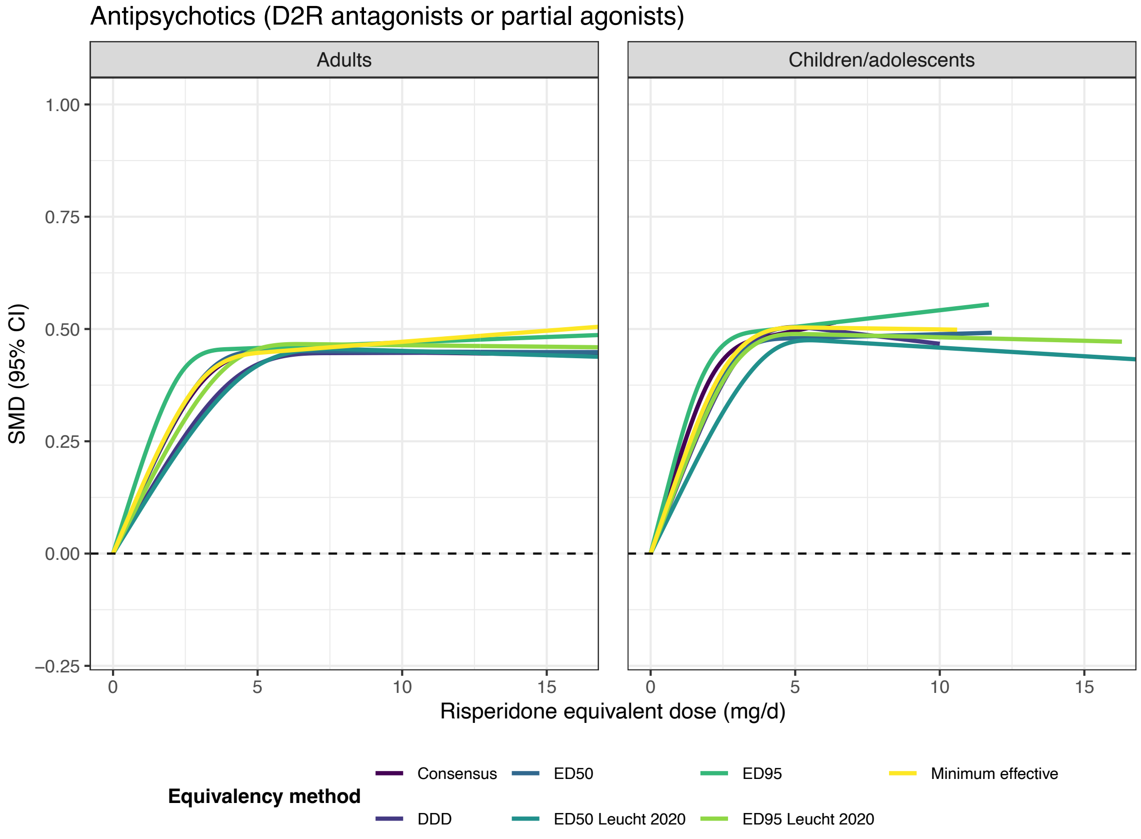

When pooling different antipsychotics, we also conducted sensitivity analyses using alternative dose equivalency methods, including 50% effective doses, minimum effective doses (Leucht et al., 2014), defined daily doses (DDD) (Leucht et al., 2016), and consensus-based antipsychotic dosing recommendations (Gardner et al., 2010; McAdam et al., 2023). The dose-response curves were also not materially changed in a sensitivity analysis by conducting a dose-response meta-regression with baseline severity.

eAppendix 12: GRADE

Approach

We used an adapted version of the GRADE approach (1) to evaluate confidence in the evidence of the dose-response curves considering the domains of risk of bias, reporting bias, indirectness, inconsistency, and imprecision. A similar adaption was used in a previous analysis for extrapyramidal side-effects associated with antipsychotics to evaluate the confidence in the evidence of a dose-response curve across all doses. We used this approach inspired by our previous dose-response meta-analyses (2).

The confidence in the evidence could be rated as very low, low, moderate or high. The confidence in the evidence for each dose-response curve started from a high level and its confidence was downrated by one or two levels due to concerns in the above-mentioned domains and according to the following criteria.

Risk of bias

We evaluated the overall risk of bias of each study as low, unclear or high using the Cochrane risk of bias tool version 1 and an algorithm of a previous review to evaluate the overall risk of bias (3). Then, we calculated the percentage of studies with moderate or high overall risk of bias that contributed to a dose-response analysis. We downrated by one level, when the percentage was between 50-75% and by two levels when it was >75%.

Reporting bias

We did not downrate the confidence in the evidence due to reporting bias as there was no clear indication of small-study effects from the funnel plots and the dose-response meta-regressions using sample size as covariate (see eAppendix-9).

Indirectness

We are especially interested in the dose-response relationship among the recommended dose range. We therefore downgraded the indirectness when there was little information among the recommended dose range (see "Cumulative number of participants per dose for each drug").

Inconsistency

Heterogeneity was quantified using the Variance Partition Coefficient (VPC), which is analogous to I-squared in pairwise meta-analysis. Confidence in the evidence was downrated by one level when the median VPC was 50-75%, and two levels when it was >75%. If the VPC could not be estimated, such as when only one study was available, confidence in the evidence was downgraded by one level, as inconsistency could not be assessed.

Imprecision

We assessed imprecision across the dose-response curve. We considered the average width of the 95%CIs, and when the average width was wide (more than twice a small effect size), we downrated by one level (e.g., when SMD upper-boundary – SMD lower-boundary>0.4) or two levels (when SMD upper-boundary – SMD lower-boundary >0.8).

When there was no imprecision according to this approach, but the total number of participants was <800, then imprecision was downrated by one level.

Cumulative number of participants per dose for each drug

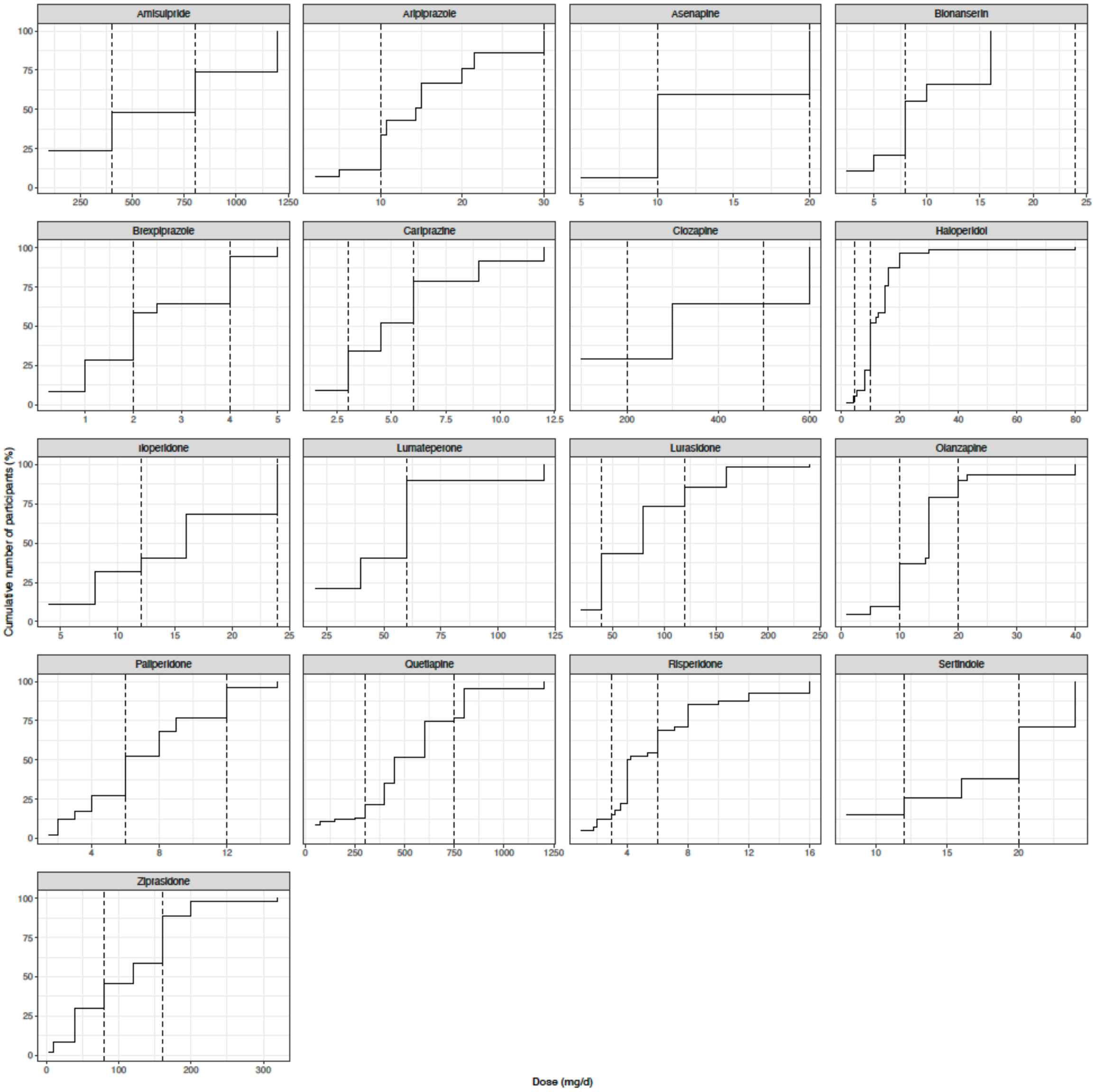

Reference

1. Guyatt GH, Oxman AD, Vist GE, Kunz R, Falck-Ytter Y, Alonso-Coello P, et al. (2008): GRADE: an emerging consensus on rating quality of evidence and strength of recommendations. *Bmj*. 336:924-926.

2. Salanti G, Peter N, Tonia T, Holloway A, White IR, Darwish L, et al. (2022): The Impact of the COVID-19 Pandemic and Associated Control Measures on the Mental Health of the General Population : A Systematic Review and Dose-Response Meta-analysis. *Ann Intern Med*.

3. Furukawa TA, Salanti G, Atkinson LZ, Leucht S, Ruhe HG, Turner EH, et al. (2016): Comparative efficacy and acceptability of first-generation and second-generation antidepressants in the acute treatment of major depression: protocol for a network meta-analysis. *BMJ open*. 6:e010919.

Confidence in the evidence for adults

Drug	Number of studies	Number of arms	Number of participants	Risk of bias	Reporting bias	Indirectness	Imprecision	Inconsistency	Confidence in the evidence
Amisulpride	1	4	248	No concerns	No concerns	No concerns	Some concerns	Some concerns	Low
Aripiprazole	12	33	3,228	Some concerns	No concerns	No concerns	No concerns	No concerns	Moderate
Asenapine	6	17	2,206	Some concerns	No concerns	No concerns	No concerns	No concerns	Moderate
Blonanserin	2	7	817	Some concerns	No concerns	Some concerns	Some concerns	Some concerns	Very low
Brexpiprazole	4	17	2,131	No concerns	No concerns	No concerns	No concerns	No concerns	High
Cariprazine	6	20	2,146	Major concerns	No concerns	No concerns	No concerns	No concerns	Low
Clozapine	1	3	48	No concerns	No concerns	No concerns	Major concerns	Some concerns	Very Low
Haloperidol	19	41	2,581	No concerns	No concerns	Major concerns	Some concerns	No concerns	Very low
Iloperidone	5	15	1,940	No concerns	No concerns	No concerns	Some concerns	No concerns	Moderate
Lumateperone	3	9	1,168	No concerns	No concerns	No concerns	Major concerns	Some concerns	Very low

Drug	Number of studies	Number of arms	Number of participants	Risk of bias	Reporting bias	Indirectness	Imprecision	Inconsistency	Confidence in the evidence
Lurasidone	11	32	3,593	No concerns	No concerns	No concerns	No concerns	No concerns	High
Olanzapine	22	52	4,807	No concerns	No concerns	No concerns	No concerns	Some concerns	Moderate
Paliperidone	11	36	4,103	No concerns	No concerns	No concerns	No concerns	Some concerns	Moderate
Quetiapine	9	32	2,978	No concerns	No concerns	No concerns	No concerns	Some concerns	Moderate
Risperidone	26	66	6,459	No concerns	No concerns	No concerns	No concerns	No concerns	High
Sertindole	4	15	1,228	No concerns	No concerns	No concerns	No concerns	No concerns	High
Ziprasidone	7	22	1,345	Major concerns	No concerns	No concerns	No concerns	No concerns	Low

Confidence in the evidence for children/adolescents

Drug	Number of studies	Number of arms	Number of participants	Risk of bias	Reporting bias	Indirectness	Imprecision	Inconsistency	Confidence in the evidence
Aripiprazole	2	6	399	Some concerns	No concerns	No concerns	Some concerns	No concerns	Low
Asenapine	1	3	228	No concerns	No concerns	No concerns	Some concerns	Some concerns	Low
Blonanserin	1	3	150	No concerns	No concerns	No concerns	Some concerns	Some concerns	Low
Lurasidone	1	3	326	No concerns	No concerns	No concerns	Major concerns	Some concerns	Very Low
Paliperidone	1	4	200	No concerns	No concerns	No concerns	Some concerns	Some concerns	Low
Quetiapine	2	5	311	No concerns	No concerns	No concerns	Some concerns	No concerns	Moderate
Risperidone	2	5	413	Some concerns	No concerns	No concerns	Some concerns	No concerns	Low

eAppendix 13: Distribution plots to examine heterogeneity

These analyses were conducted only in adult studies, given the sparser data for children/adolescents.

Mean age

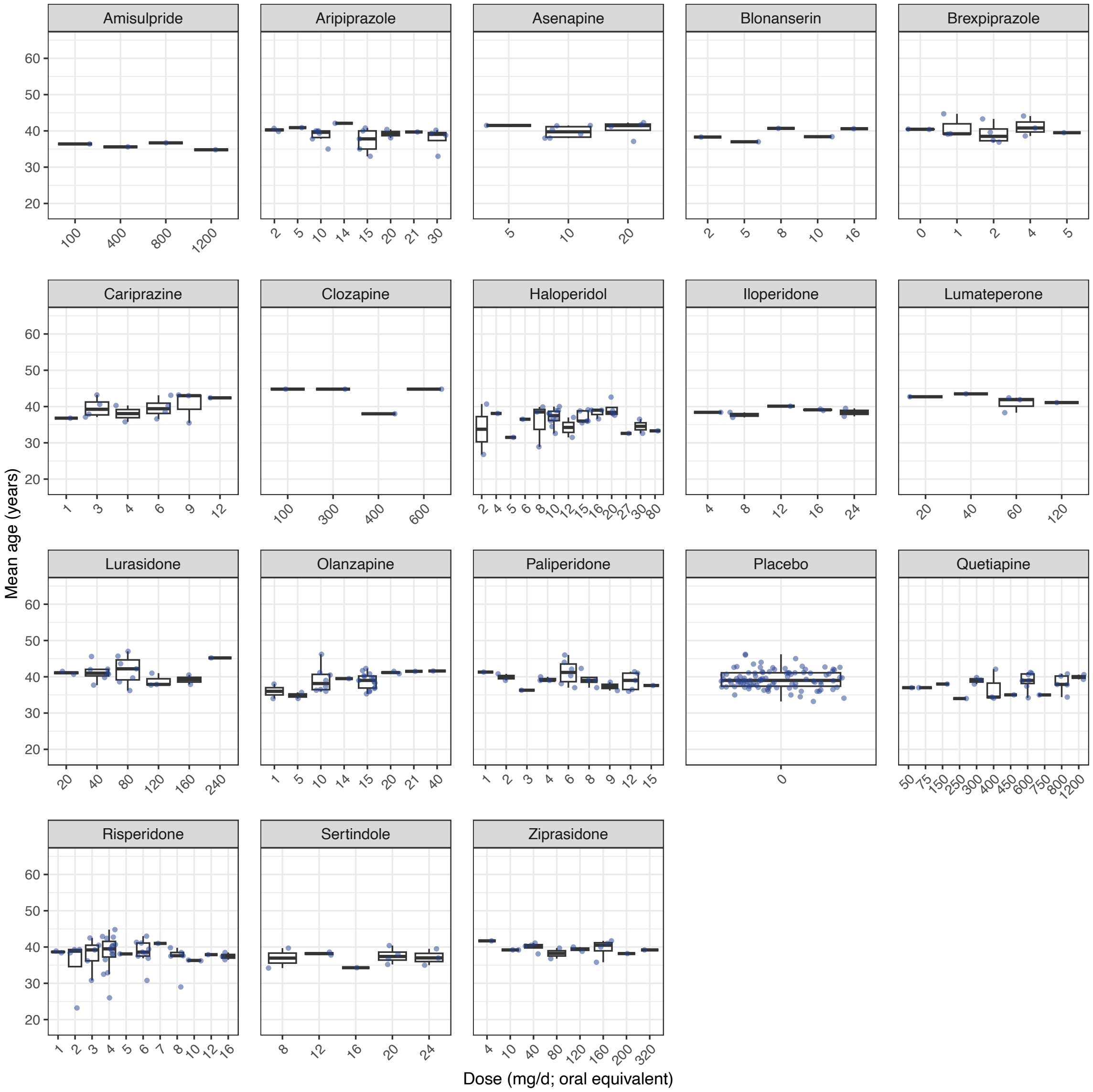

Trial duration

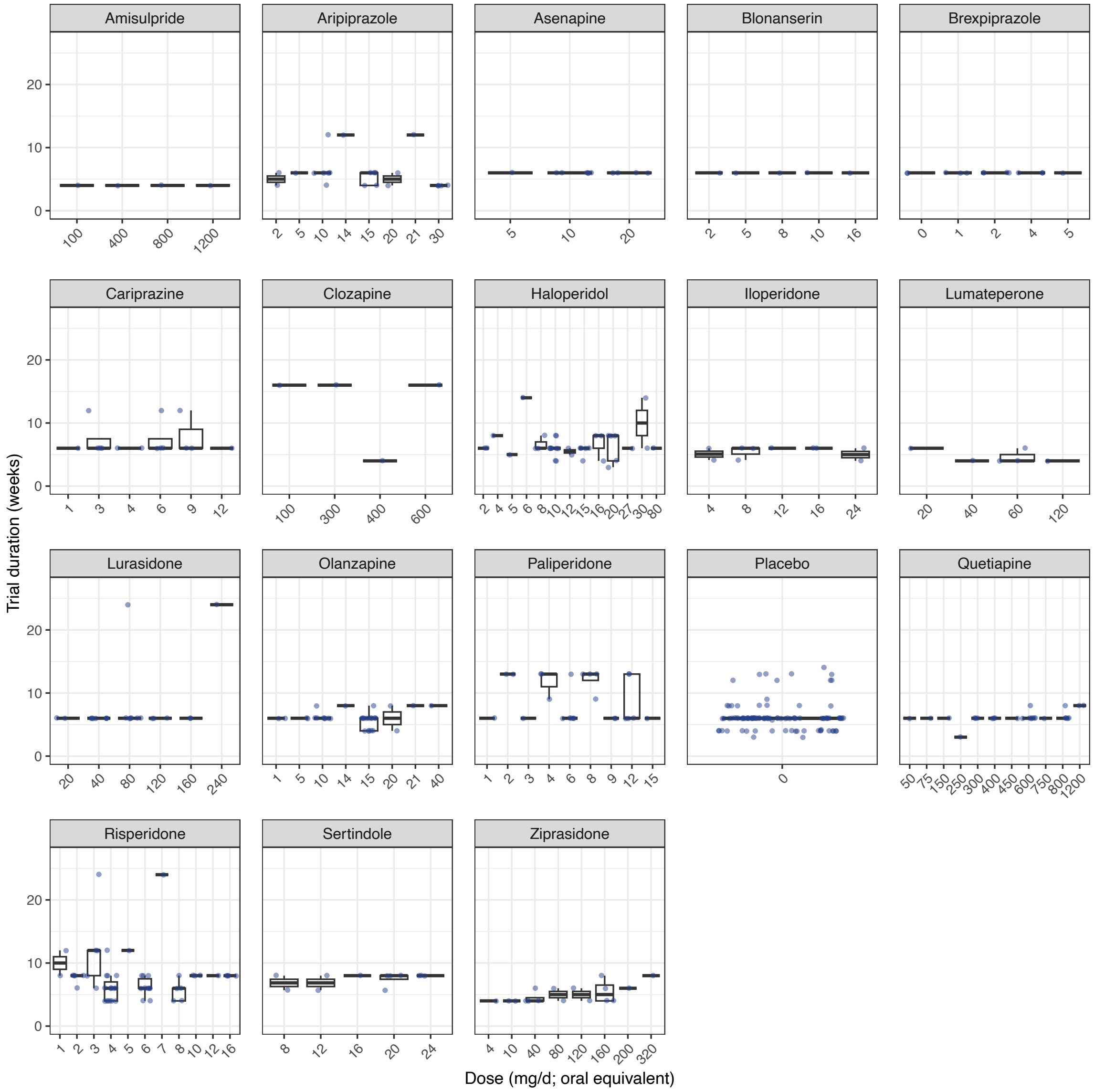

Proportion of males

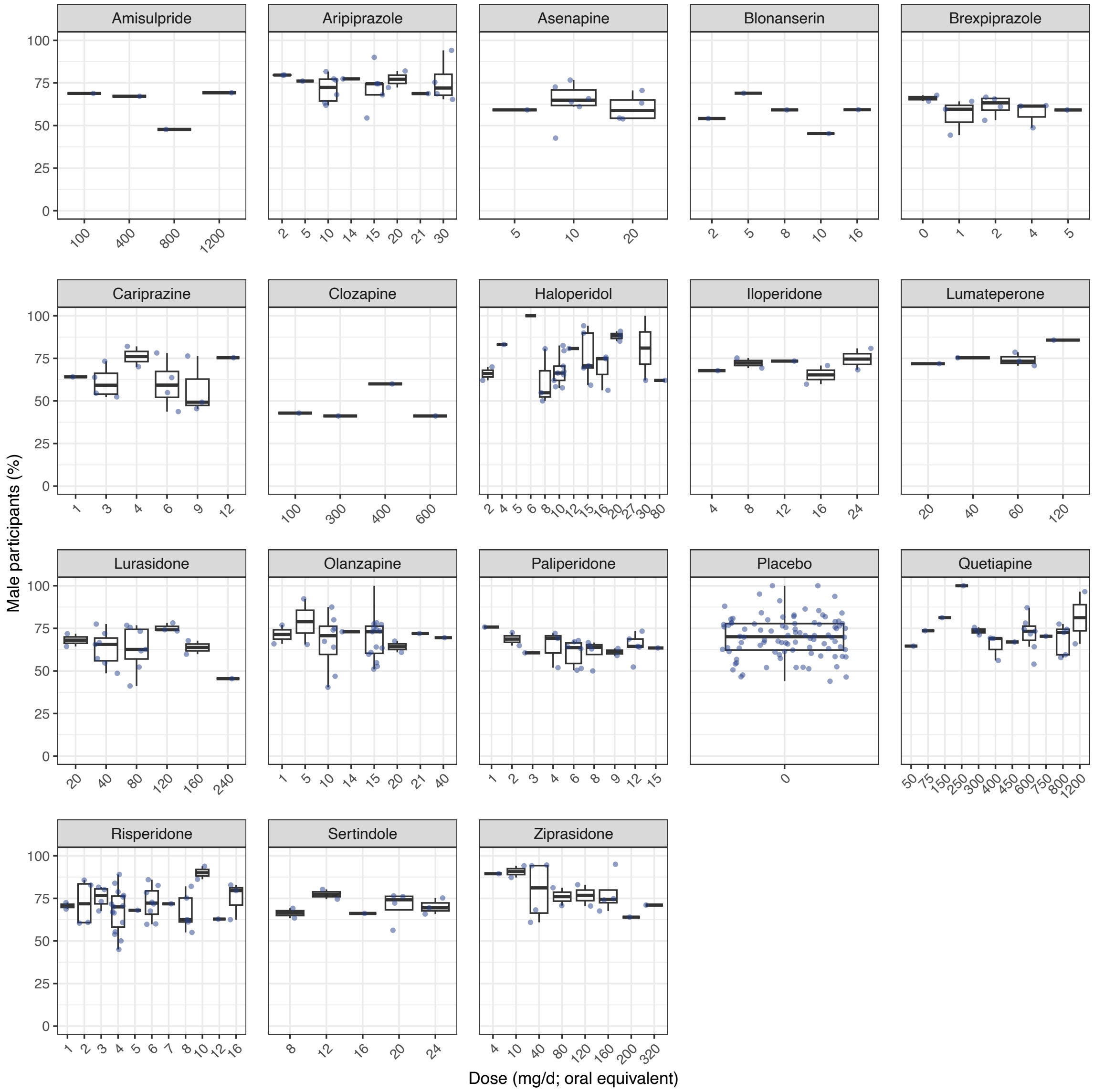

Mean baseline PANSS score

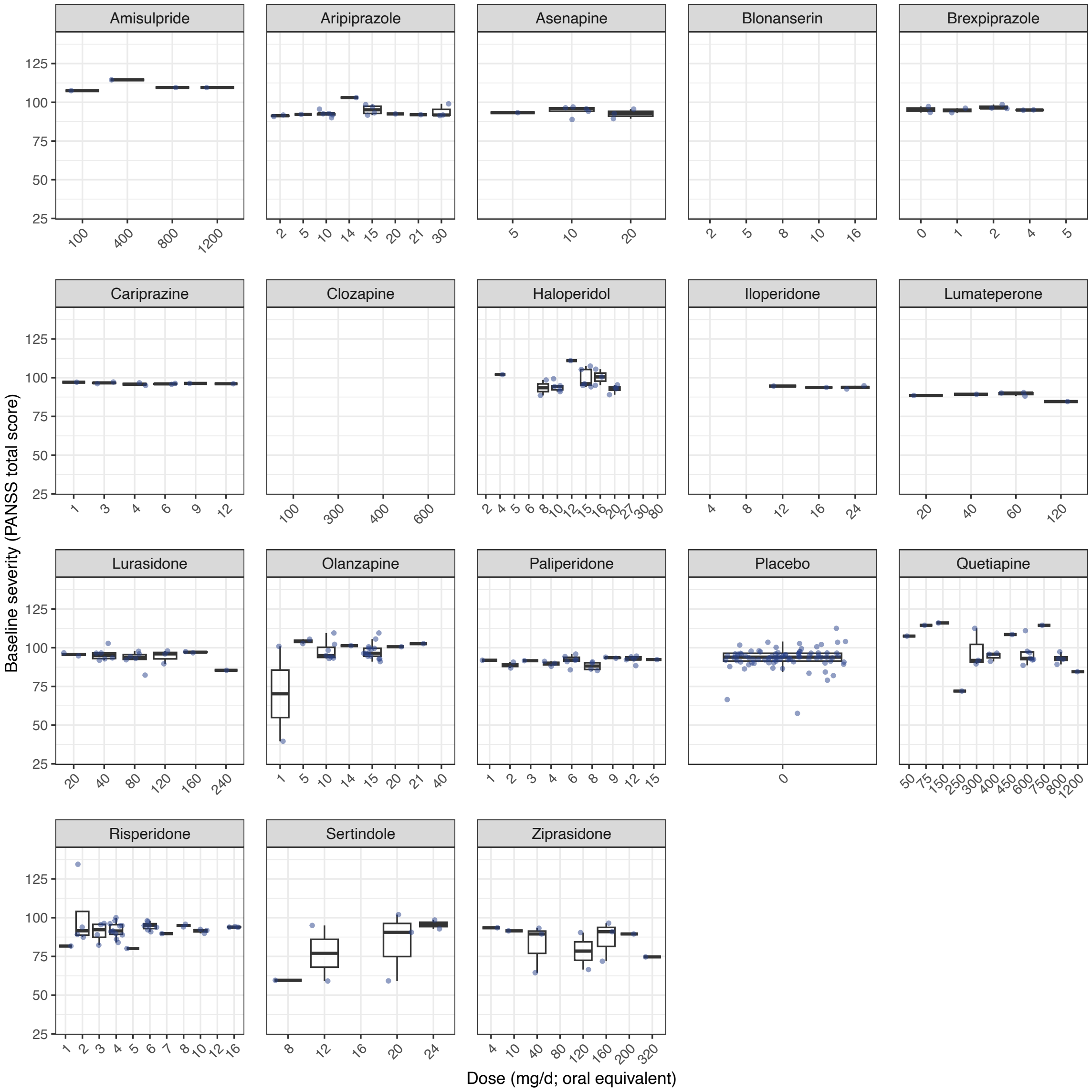

Risk of Bias

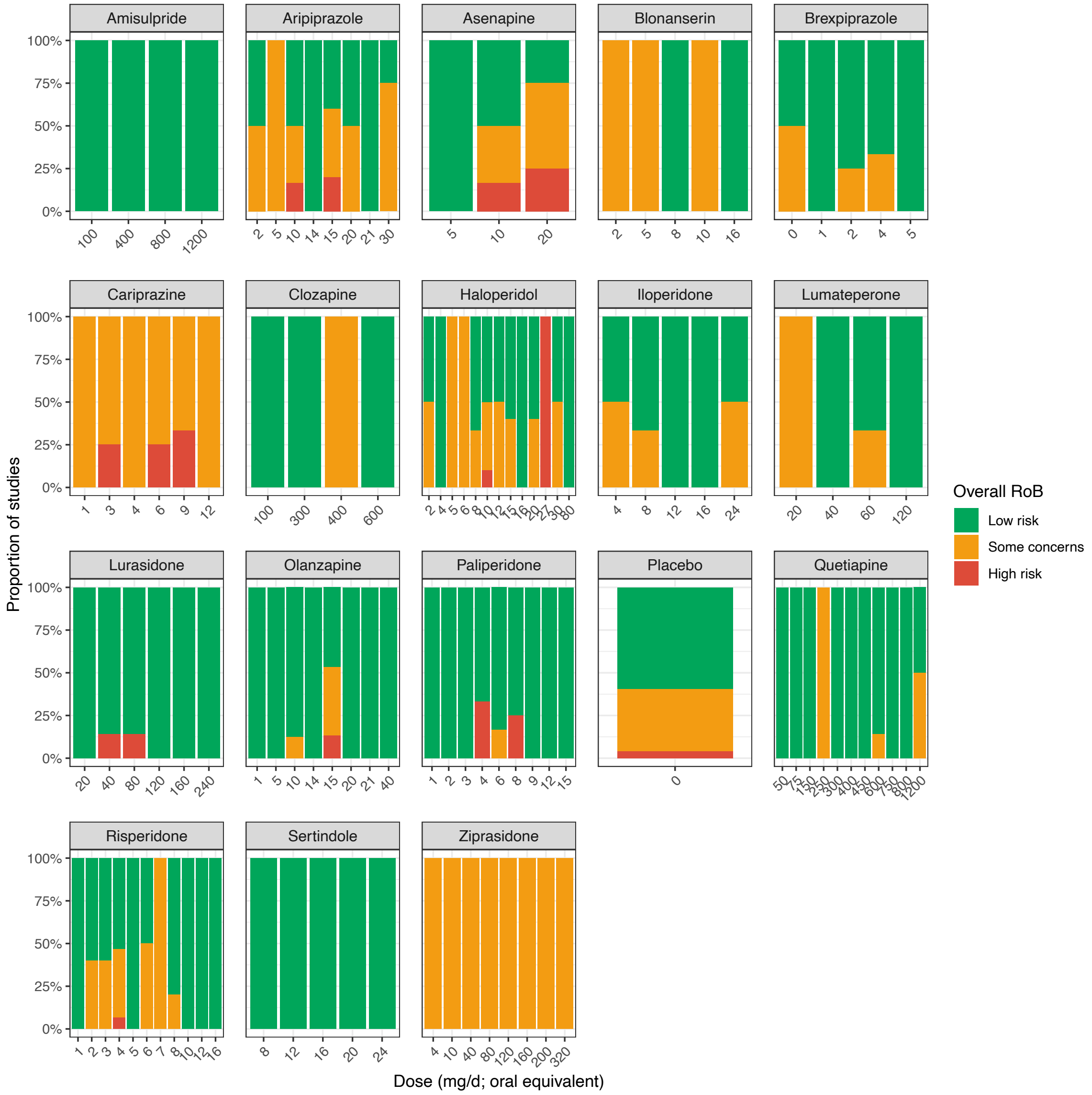
Amisulpride
Aripiprazole
Asenapine
Blonanserin
Brexpiprazole
Cariprazine
Clozapine
Haloperidol
Iloperidone
Lumateperone
Lurasidone
Olanzapine
Paliperidone
Placebo
Quetiapine
Risperidone
Sertindole
Ziprasidone
Proportion of studies
Dose (mg/d; oral equivalent)
Overall RoB
Low risk
Some concerns
High risk
